# A network approach reveals driver genes associated with survival of patients with triple-negative breast cancer

**DOI:** 10.1016/j.isci.2021.102451

**Published:** 2021-04-19

**Authors:** Courtney D. Dill, Eric B. Dammer, Ti'ara L. Griffen, Nicholas T. Seyfried, James W. Lillard

**Affiliations:** 1 Department of Microbiology, Biochemistry, and Immunology, Morehouse School of Medicine, 720 Westview Dr SW, HG 341B, Atlanta, GA 30310, USA; 2 Center for Neurodegenerative Disease, Emory University School of Medicine, Atlanta, GA 30322, USA; 3 Department of Biochemistry, Emory University School of Medicine, Atlanta, GA 30322, USA; 4 Department of Neurology, Emory University School of Medicine, Atlanta, GA 30322, USA

**Keywords:** Bioinformatics, Systems biology, Cancer systems biology

## Abstract

We aimed to identify triple-negative breast cancer (TNBC) drivers that regulate survival time as predictive signatures that improve TNBC prognostication. Breast cancer (BrCa) transcriptomic tumor biopsies were analyzed, identifying network communities enriched with TNBC-specific differentially expressed genes (DEGs) and correlated strongly to TNBC status. Two anticorrelated modules correlated strongly to TNBC subtype and survival. Querying module-specific hubs and DEGs revealed transcriptional changes associated with high survival. Transcripts were nominated as biomarkers and tested as combinatoric ratios using receiver operator characteristic (ROC) analysis to assess survival prediction. ROC test rounds integrated genes with established interactions to hubs and DEGs of key modules, improving prediction. Finally, we tested whether integration of literature-derived genes for implicated hallmark cancer processes could improve prediction of survival. Complementary coexpression, differential expression, genetic interaction, and survival stratification integrated by ROC optimization uncovered a panel of “linchpin survival genes” predictive of patient survival, representing gene interactions in hallmark cancer processes.

## Highlights

• WGCNA identifies coexpression modules predicted to drive TNBC patient survival
• Module hubs and DEGs reveal transcriptional changes associated with high survival
• Nine genes act synergistically to influence TNBC progression, relapse, and survival
• These genes' levels represent reversible changes in TNBC hallmark cancer processes

## Graphical abstract

**Figure:**
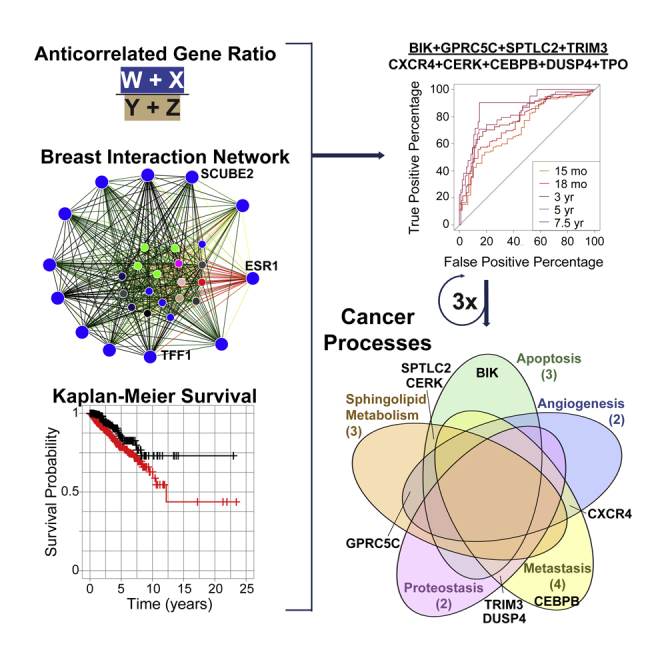

## Introduction

Triple-negative breast cancer (TNBC) accounts for 10 to 20% of all invasive breast cancer (BrCa) cases and lacks estrogen receptor, progesterone receptor, and human epidermal growth factor receptor 2 (HER2) responsiveness. TNBC is an aggressive cancer associated with poor prognosis relative to non-TNBC, that is, higher and earlier risk of relapse and recurrence, as well as poor survival. The 5-year overall and disease-free survival rates are 62.1% and 57.5% for TNBC versus 80.8% and 75.3% for non-TNBC, respectively (p < 0.001) (Goncalves et al., 2018).

Owing to loss of receptor signaling, TNBC does not respond to hormone receptor or HER2-directed therapies. Instead, standard therapy consists of a combination of chemotherapeutic drugs demonstrating marginal efficacy. The ineffective nature of current TNBC therapies suggests that the genetic and molecular patterns associated with TNBC progression are complex with interconnected dysregulation. Thus, there is a need to understand the molecules and mechanisms associated with survival of patients with TNBC at a systems level, which will allow for better prediction of gene targets in TNBC and improve TNBC prognostication.

In line with known poor TNBC prognosis, this subtype exhibits enhanced evasion of apoptosis, angiogenesis, and metastasis, among hallmark cancer processes as defined by Hanahan and Weinberg (Hanahan and Weinberg, 2011). Although there have been advances in our understanding of the biology of primary breast tumors relating to these and other hallmark processes, our knowledge of how and why patients with TNBC experience poor prognoses compared with patients without TNBC is limited and warrants a circumspect unbiased analysis.

Using an integrative systems biology approach, we performed a cross-platform meta-analysis of large numbers of BrCa transcriptomes curated in The Cancer Genome Atlas (TCGA; N = 777) and GEO DataSets (N = 1,234) to assess the correlation between gene transcript expression in coexpressed transcript modules to sample traits relevant to TNBC. The network structure was leveraged to nominate genes whose transcript levels in combination are tied to survival in either generalized BrCa or specifically TNBC, nominating genes to predict patient survival. Genes were then combined in a ratio with choice of numerator or denominator set by membership in anticorrelated modules of interest. The best of the nominated gene combinations we ranked by receiver operator characteristic (ROC) analysis implicate both known and potentially unappreciated genes in hallmark dysregulated cancer processes.

Overall, this study successfully leveraged the coexpression network of the various BrCa subtypes in large transcriptomic cohorts including patients with TNBC, overlapped this structure with differential expression, selected candidate transcripts influencing survival overall in BrCa, and then homed-in on TNBC-specific linchpin survival genes. Selection of genes implicated in the gene interaction network of breast tissue also contributed to the optimized survival gene list. Mining of the prognosis indicators nominated here for testing in future work is warranted to discover the complex molecular and functional interactions among hallmark cancer process genes driving mortality in patients with TNBC, with TNBC subtype-specific treatment outcomes.

## Results

### Overview of systems-biology-leveraged survival gene discovery workflow

**Figure 1:**
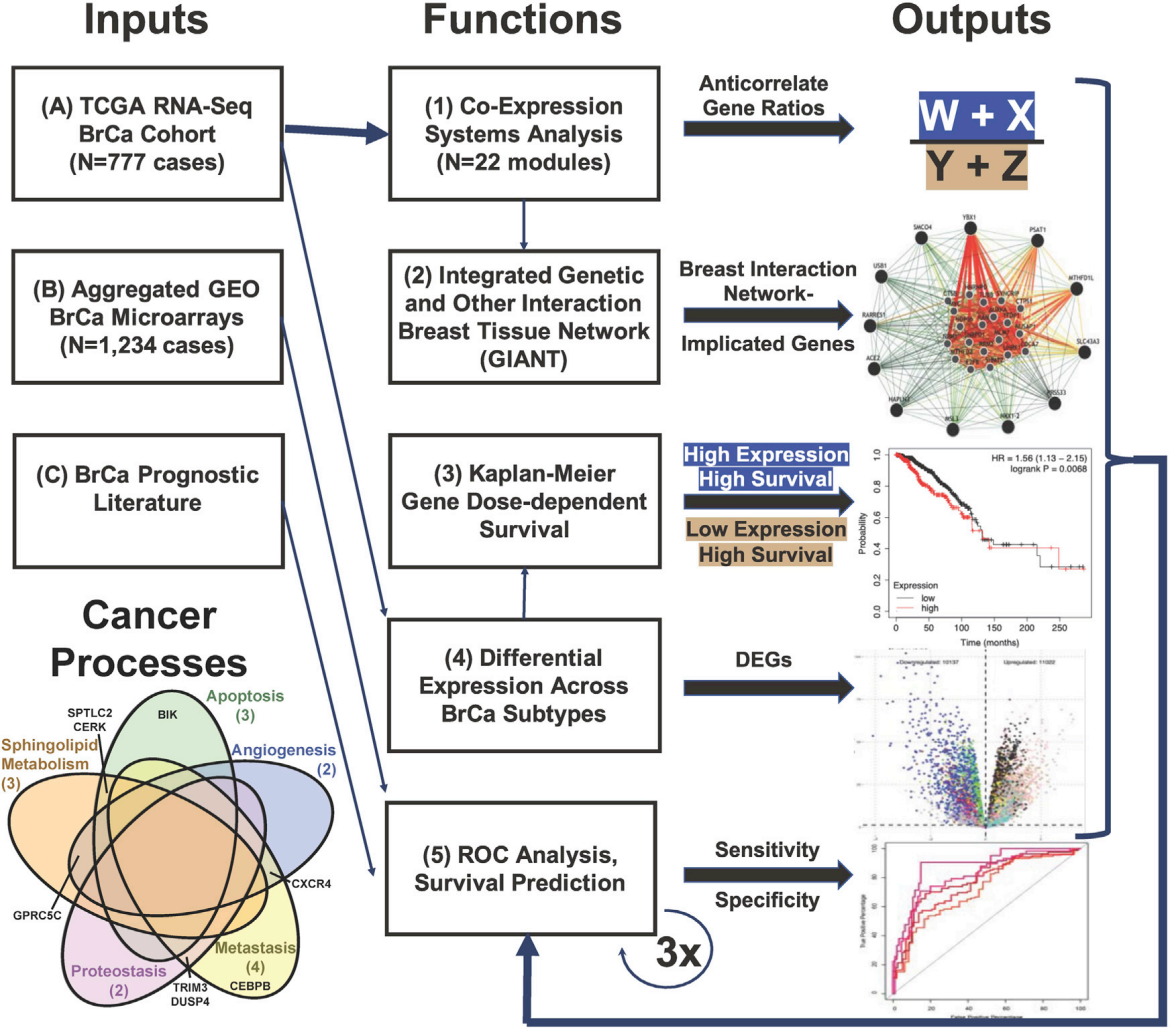
Overview of analysis workflow for this study As described in results section 1. See also Figures S4 and Table S1. Patient characteristics of TCGA BRCA population, related to Figure 1, Table S2. Breast cancer staging breakdown of cases in TCGA RNA-seq, related to Figure 1, Table S3. RNA-seq case traits used for coexpression analysis (N = 773), related to Figure 1, Table S10. Microarray case traits used for ROC testing (N = 1,234), related to Figure 8, Table S11. Round 1 ROC survival predictor gene expression ratio using Affy Arrays (N=1,234), related to Figure 8 and Table 3, Table S12. Round 2 ROC survival predictor gene expression ratio using Affy arrays (N=1,234), related to Figure 8 and Table 3.

The phenotype of less severe BrCa subtypes, such as Luminal A, Luminal B, and HER2-enriched types are well characterized, which has led to the discovery of drug therapies that have increased survival time for these patient groups. However, the molecules and mechanisms responsible for the more lethal phenotype of TNBC involve more complex interactions and presently, a targeted therapy for TNBC does not exist. This unmet need fostered our development of a nontraditional pipeline to define and survey the systems biology landscape of BrCa and TNBC for identifying TNBC targets (Figure 1). The literature for BrCa of the past >20 years is strewn with reports of prognostic indicators which validate but are not part of an integrated framework of understanding. Here, we leverage differences in transcript levels unique to TNBC in the context of systems biology: **(Function 1)** a coexpression network encompassing all BrCa subtypes' RNA quantitation (N = 777; TCGA RNA-seq cohort—input **(input A)**; a breakdown of clinical traits and tumor staging for the TCGA BRCA cohort is provided in Table S1. Patient characteristics of TCGA BRCA population, related to Figure 1, Table S2. Breast cancer staging breakdown of cases in TCGA RNA-seq, related to Figure 1, Table S3. RNA-seq case traits used for coexpression analysis (N = 773), related to Figure 1, Table S10. Microarray case traits used for ROC testing (N = 1,234), related to Figure 8, Table S11. Round 1 ROC survival predictor gene expression ratio using Affy Arrays (N=1,234), related to Figure 8 and Table 3, Table S12. Round 2 ROC survival predictor gene expression ratio using Affy arrays (N=1,234), related to Figure 8 and Table 3**. (Function 2)** Genetic interactions specific to breast tissue from the genome-scale integrated analysis of gene networks in tissues (GIANT) analysis framework (Wong et al., 2018) also were integrated into the nomination of genes that can predict survival and therefore are most likely to affect it. The more traditional **(function 3)** Kaplan-Meier (KM) survival analysis focused on coexpression network hubs of key TNBC-associated modules intersecting with **(function 4)** differentially expressed genes in BrCa subtypes. The KM significant genes were nominated transcripts for **(function 5)** ROC survival prediction that were moderately predictive in both BrCa in general and TNBC specifically as assessed by predictor area under the curve (AUC) in an independent meta-analysis of 1,234 array-measured BrCa transcriptomes—**(input B)**. More than 100,000 combinations of genes encompassing a small tract of the total network landscape of relevant differentially expressed gene combinations were tested by an additional round of ROC to improve survival prediction specifically in TNBC, where the genes ranked best by AUC shifted when focusing only on the 180 TNBC cases in the array metanalysis. The resulting top ranked prognostic indicator specific to TNBC was finally improved once more by identification of the hallmark cancer processes implicated by the genes already found though our unbiased nomination process, and a final network-informed addition of known gene products from literature **(input C)** regarding those hallmark cancer processes highly relevant to BrCa (Figure 1, Venn diagram at bottom left). We then validated the array data (**input B**) ROC test result using ROC analysis of the same genes found in TCGA input data **(input A)**. A total of 111,385 combinations of genes were evaluated using area under the ROC curve (AUC) analysis. The final ratio of gene abundances significantly outperformed prediction of survival of patients with TNBC compared with patients without TNBC, identified as those best improving survival prediction of patients with TNBC. An extensive literature search demonstrated final gene indicators are associated with hallmark cancer processes. Our final indicator identified sphingosine/ceramide balance, balance of proapoptotic and antiapoptotic signaling, proteostasis, angiogenesis, and metastasis as processes regulated by our indicator genes. Importantly, while our results are informed by known gene signatures, 7 of 9 of the survival-linked gene products, which we nominate, were arrived at via unbiased exploration of the network landscape.

### Identification of coexpressed BrCa biopsy transcript communities and initial selection of a subset of modules associated with TNBC.

Coexpression simplifies biomarker panel selection and enables gene equivalence determinations for consolidation of such panels, including ones for molecular and other BrCa subtypes (Wirapati et al., 2008). After cleanup, batch correction with normalization, and outlier removal, the BrCa transcriptome containing 31,338 gene products across 773 nonoutlier BrCa (including 92 TNBC) tumor samples was clustered into coexpressed communities (modules) of gene transcripts using WGCNA with a 1-minus topological overlap matrix metric based on pairwise gene transcript correlations in an adjacency matrix calculated with biweight midcorrelation (bicor) for robust correlation, as described in methods. Bicor is a built-in correlation feature of WGCNA based on median provided as an alternative to Pearson correlation which is based on mean. Bicor was used, as opposed to Pearson correlation, to provide robust correlations with less weight given to outlier measures (Oldham et al., 2008; Langfelder and Horvath, 2012). A total of 22 network coexpression modules and corresponding quantitative module eigengenes (MEs) (for modules numbered by their size rank from largest to smallest) were identified: M1 to M22. The log_2_ relative FPKM/central tendency weighted first principal component equivalent to each of the 22 MEs is provided in Table S4. Table S5 provides the complete list of gene transcripts referenced by module membership and Pearson correlation to each of the 22 MEs (kME), where these correlations correspond to their weighted contribution to the ME in which they fall.

**Figure 2:**
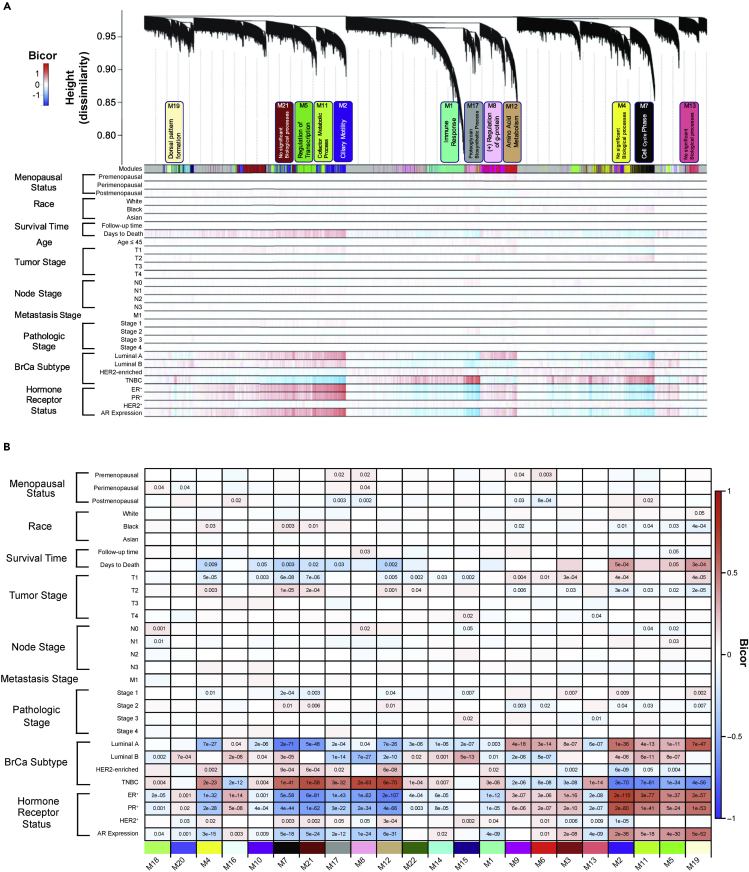
Coexpression network analysis of BrCa transcripts identified modules enriched with markers of biological processes and module-trait correlations reveal module communities relevant to BrCa subtype (A) In the first row below the dendrogram, each colored vertical streak represents a gene with membership in the module of that color, which contains a group of highly coexpressed transcripts. A total of 22 modules were identified. A Kruskal-Wallis test among the four subtype-specific case groups (Luminal A, Luminal B, HER2-enriched, and TNBC) with significance set to 0.05, and/or TNBC binary trait bicor revealed 12 modules (M12, M21, M8, M17, M7, M2, M11, M5, M19, M4, M13, and M1) were significantly associated with the TNBC subtype. (B) Heatmap of module trait relationships. Overlaid numbers in panel B are Student's p values for bicor significance of trait correlation to the module eigengenes. Module-Trait bicor color scale (−1, blue; 0, white; +1 red) indicates modules with significant Student's p cluster together, in particular into M2-like and M12-like clusters. See also Data S1 and S2. See also Tables S4 and S5.

**Figure 3:**
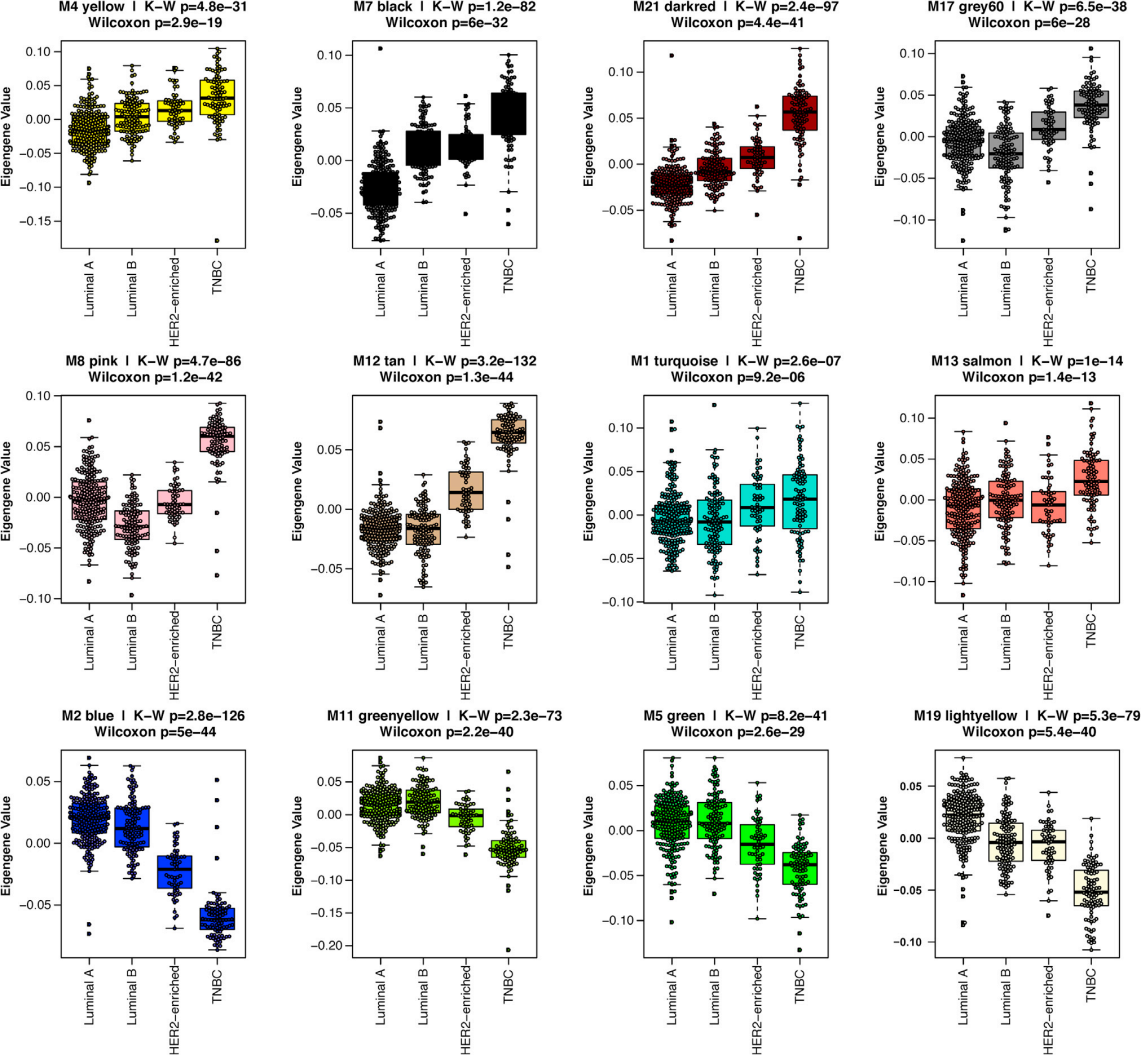
Differences in eigengene values for M2-and M12 -like modules across 4 BrCa subtypes Box plots displaying module eigengene values among the four BrCa subtypes are displayed with ANOVA Kruskal-Wallis values p < 0.05. A Wilcoxon rank-sum test assessed significant difference between TNBC and non-TNBC (Luminal A, Luminal B, and HER2-enriched) cases. Significance was also set to p < 0.05. For box plots, N = 493. See also Data S1 and S2.

The communities clustered and indicated with module colors below the dendrogram also displayed consistency among individual transcripts that correlate within each module positively (red in gene-level heatmap) or negatively (blue) with factors influencing BrCa diagnosis (Figure 2A). However, modules are considered as a weighted average of gene members in the ME calculation, and each ME (or simply, the module each summarizes) can be correlated to available traits to select modules with significant trait correlations of interest, alleviating multiple testing greatly. We chose to focus on modules with significant correlation to the TNBC subtype binary trait to identify gene communities relevant to TNBC biology. First, to identify subtype-associated modules, Kruskal-Wallis ANOVA p < 0.05 indicating significant overall separation among the four BrCa subtypes (Luminal A, Luminal B, HER2-enriched, and TNBC) revealed 12 modules (M4, M7, M21, M17, M8, M12, M1, M13, M2, M11, M5, M19) (Figure 2B). The box plots for these modules display the relative expression within each module. With the exception of M1, M4, and M17, the eigengene values for TNBC were qualitatively different from the other receptor-positive subtypes (Figure 3). In addition, a Wilcoxon rank-sum test indicated significant difference between TNBC and non-TNBC (Luminal A, Luminal B, and HER2-enriched) cases. Importantly, the intersection of this twelve-module list with the list of MEs significantly correlated to the TNBC subtype binary trait was complete, with significance of correlation ranging from 3.0x10^−6^ (M1) to 3.0x10^−70^ (M2) (Figure 2B). These modules were biologically coherent, over-representing the ontologies listed in Figure 2A. Gene ontology enrichment analysis was performed on the aforementioned modules correlated to the TNBC subtype trait via GO-Elite (Zambon et al., 2012), identifying coherent biology of coexpressed transcript modules. A list of the top five biological processes with false discovery rate (FDR)-adjusted p values for each of these modules is in Table 1.

**Table 1:** Ontology enrichment of modules with correlation to TNBC versus receptor positive BrCa status

Module and *subtype ANOVA p*	Top biological processes	GO-Elite FET FDR	
Yellow (M4) p = 4.8E-31	No significant biological processes associated	NA	
Black (M7) p = 1.2E-82	Cell cycle phase Cell cycle DNA metabolic process Cell division Regulation of cell cycle process	7.80E-87 2.84E-93 1.04E-49 7.12E-41 5.99E-34	M12-like
Darkred (M21) p = 2.4E-97	No significant biological processes associated	NA	
Grey60 (M17) p = 6.5E-38	Proteoglycan biosynthetic process Actin filament-based process Cell junction organization Skeletal system development Cellular component movement	5.53E-02 6.01E-04 4.17E-02 4.42E-02 1.88E-02	
Pink (M8) p = 4.7E-86	Positive regulation of g-protein Hemidesmosome assembly Tissue development Positive regulation of neuroblast proliferation Positive regulation of endothelial cell migration	1.98E-04 7.24E-04 1.35E-07 7.74E-03 2.92E-03	
Tan (M12) p = 3.2E-132	Cellular modified amino acid metabolic process	2.96E-02	
Turquoise (M1) p = 2.6E-07	Immune Response Regulation of immune response Positive regulation of immune response Defense response Response of lymphocyte activation	3.22E-161 6.95E-91 5.12E-73 2.67E-73 4.93E-53	
Salmon (M13) p = 1.0E-14	No significant biological processes associated	NA	
Blue (M2) p = 2.8E-126	Ciliary or flagellar motility GPI anchor metabolic process Phototransduction Positive regulation of glucose import Branched chain family amino acid metabolic process	3.24E-02 3.76E-01 7.77E-01 8.41E-01 9.33E-01	M2-like
Greenyellow (M11) p = 2.3E-73	Cofactor metabolic process Oxidation-reduction process Lipid metabolic process Thioester metabolic process Cofactor metabolic process	1.95E-02 4.67E-02 9.73E-02 7.81E-01 1.95E-02	
Green (M5) p = 8.2E-41	Regulation of transcription, DNA-dependent Androgen receptor signaling pathway Protein modification by small protein conjugation or removal Organelle organization Nuclear-transcribed mRNA poly(A) tail shortening	4.48E-16 3.10E-03 1.55E-04 3.96E-04 3.03E-02	
Lightyellow (M19) p = 5.3E-79	Dorsal/ventral pattern formation Forebrain development Epithelial tube morphogenesis Specification of symmetry Regulation of embryonic development	1.69E-03 1.53E-01 3.21E-01 3.21E-01 4.00E-01	

A one-tailed Fisher’s exact test with FDR correction detected significant overlap between GO lists of gene symbols and members of each module.

Box plots for the top positively correlated module to TNBC (M12, bicor = 0.69 and p = 6.0x10^−70^; amino acid metabolism) and the top negatively correlated module (M2, bicor = −0.69 and p = 3.0x10^−70^; ciliary motility) in Figure 3 show starkly higher and lower expression in TNBC relative to receptor-positive subtypes in these two opposing modules, respectively. These two modules, as represented by their top hub kME values for each other, indicate that M2 and M12 are also the most strongly anticorrelated to each other (Table S5, red versus green scale for positive versus negative Pearson correlations of hubs in M2, positive for kME_blue_ and negative for kME_tan_, and in M12, *vice versa*).

Finally, module trait relationships were assessed for confounding variables by regressing out variables, for example, age and race, before recalculation of the eigengenes (Data S2). Significant correlation remained between the 12 modules and TNBC after regression of selected traits. In previous BrCa racial disparity studies, major gene expression differences have been observed between Black and white patients, but after adjusting for proportional differences in molecular subtypes, such differences were significantly reduced or nullified (Liu et al., 2018). There is indeed a lack of race, menopause (which we did not regress), and age correlation of any significance in Data S2, page 3 and particularly page 4. This supports a conclusion that subtype differences are not confounded by differences in these traits. Analysis was performed on the unregressed data set because these traits were particularly not well correlated before regression, Figures 2B and S2, page 4. Notably, we chose to focus on BrCa subtypes independent of stage because subtype was a major driver of the network structure, as indicated by the large proportion of modules with significant correlation to the binary trait (TNBC/non-TNBC). Late-stage TNBC was underrepresented in our cohort, which reduced the statistical power to determine correlation.

### Module relatedness, correlation to molecular BrCa traits, and known indicators of TNBC

**Figure 4:**
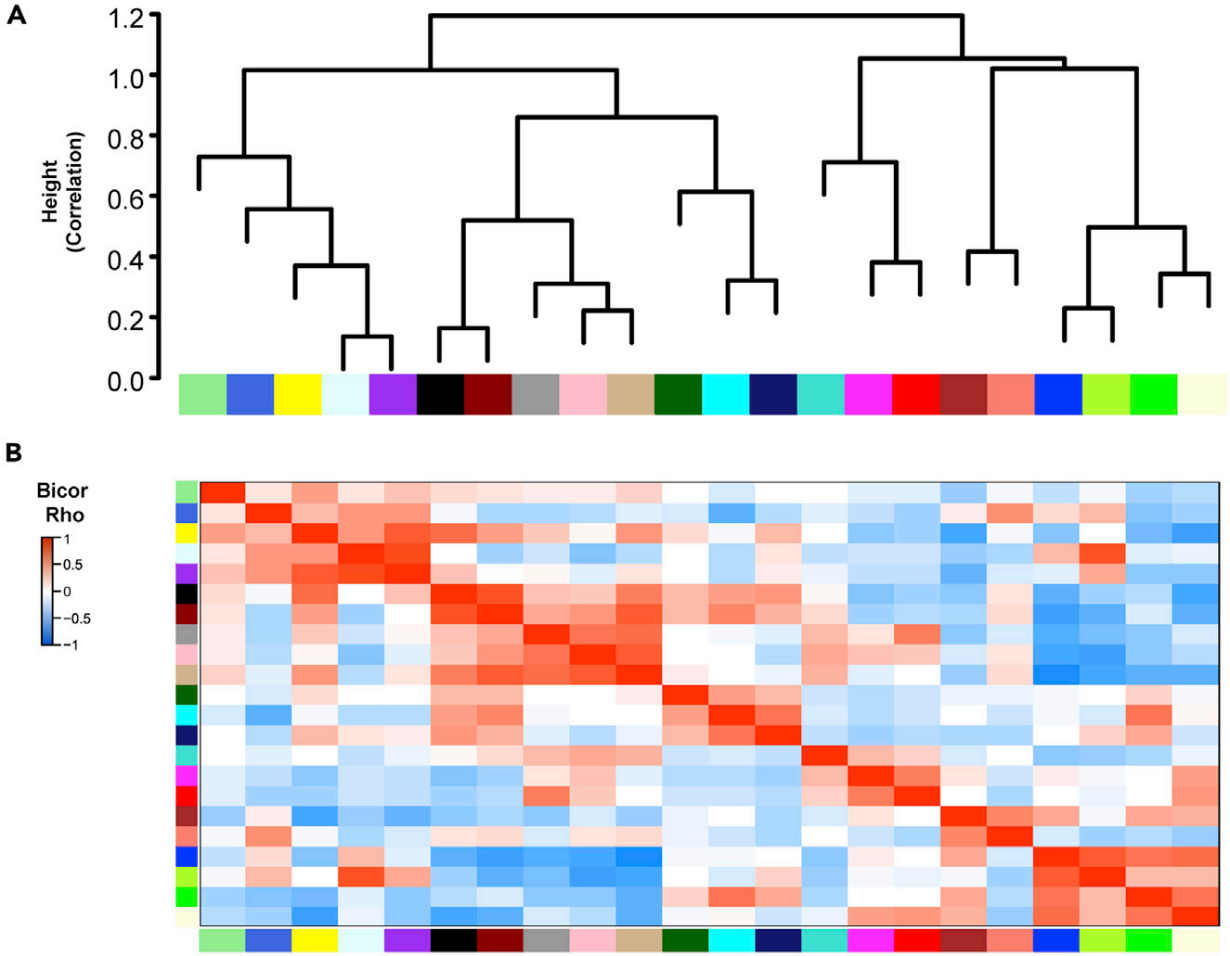
Module relatedness clustering (A) Clustering dendrogram based on ME correlation. (B) Heatmap of 22 module communities organized by module related order.

Module relatedness was determined via clustering based on correlation distance metric (Figure 4A). Three clusters were identified. The first cluster at the left contained five modules: M18, M20, M4, M16, and M10. The second cluster included five modules: M7, M21, M17, M8, and M12, and the third cluster contained nine modules: M1, M9, M6, M3, M13, M2, M11, M5, and M19; of which, the last 4 were highly similar in their trait correlations and relatedness to each other. The heatmap in Figure 4B displays the total 22 module communities in module-related order. All available quantitative and binary-coded clinical traits were correlated to MEs pairwise for samples which had the traits specified. This greatly reduces the multiple testing problem from 31,338 individual genes to 22 modules and presents modules that represent transcript network structure, with some modules strongly correlated to traits of the BrCa cases, making them candidates for harboring key drivers of molecular causality, either upstream or downstream of the highly correlated traits of interest such as subtype (Luminal A, Luminal B, HER2-enriched, TNBC), hormone-receptor-reported status (ER^+^, PR^+^, HER2-enriched, AR levels), and survival time (days to death for individuals succumbing to BrCa) (Figure 2B and Data S1, page 4).

We used module correlation (bicor) to a TNBC binary sample trait, to assess subtype differences. Bicor ranging from −1 to +1 represents eigengene (composite weighted within-module gene) expression correlation independently to each of the traits, but separate correlations are made to molecular traits, such as PR^+^ and ER^+^ expression, on a sample-by-sample basis. The heatmap in Data S1, page 4 displays p value significance for bicor between the 22 modules and the final BrCa factors that had strong correlation to days to death, BrCa subtype, and hormone receptor status. Our goal was to identify modules that both positively and negatively correlate to TNBC binary status and patient survival so we could in turn identify the relative expression pattern or phenotype of TNBC and non-TNBC. These anticorrelated genes were used to predict survival of patients during later analyses. The block of 5 modules on the left (M7, M21, M17, M8, M1) significantly and positively correlated to TNBC binary status and they are positively correlated to each other as related patterns across all cases in the network. This block of 5 modules also negatively correlated to survival time suggesting there are likely key drivers in the module that could be influencing TNBC survival. The block of 4 modules on the right (M2, M11, M5, M19) negatively correlated to TNBC and positively correlated to days to death suggesting these genes are also likely key drivers in the module that could be influencing TNBC survival.

Ultimately, we chose to focus on the M12 and M2 modules because they were prototypic, (anti-) correlated modules among seven other module communities (M7, M21, M17, M8, M11, M5, M19) that significantly correlated to TNBC binary status and patient survival time.

Four non-TNBC modules (M2, M11, M5, and M19) negatively correlated with the TNBC subtype trait with significance of correlation p < 1x10^−25^, and five TNBC modules (M7, M21, M17, M8, and M12) were just as significantly positively correlated. Three modules (M1, M4, and M13) were not closely related and were not included for further analysis. Secondary analysis could focus on these three unrelated modules. Notably, M7, M21, M17, M8, and M12 (higher expression specific to TNBC) and M2, M11, M5, and M19 (lower in TNBC) moderate-to-high significance of correlation was consistent across all four subtype-specific binary traits, and correlation itself was along a negative-to-positive (or *vice versa*) continuum across the four subtypes ordered by increasing severe/poor prognosis (Data S1, page 4). The trait for days to death also significantly correlated in opposing directions to M12 and M2, respectively (Data S1, pages 16 and 25). Hormone and HER2 receptor status across all BrCa subtypes also were the strongest and most significantly correlated to M2 and M12, in opposing directions (Data S1, page 4). A similar heatmap of all 22 modules correlated to the full set of available quantitative or binary traits can be found in Figure 2B.

Overall, these module correlations to known molecular determinants of BrCa severity and survival implicate these twelve modules in mechanisms of BrCa that may not be fully appreciated. Fully consistent with quantitation of the key BrCa receptors correlating positively to M2, notable members of M2 include its hub, one of two classic nuclear hormone receptors for estrogen (ESR1 gene for ERα, kME_blue_ = 0.83) and the nuclear hormone receptor for progesterone (kME_blue_ = 0.70). HER2 receptor (ERBB2) was not assigned to any module, with no absolute value of kME greater than 0.30, though it was weakly correlated best with a kME to M2 of 0.27, and the related receptor ERBB4 has blue (M2) membership with kME 0.70. Therefore, M2 and its strongest anticorrelate M12 are the top module candidates harboring genes with potential as baseline-measured prognostic if not also mechanistic indicators for TNBC. Thus, we termed M2, M11, M5, and M19, M2-like and M12, M7, M21, M17, and M8, M12-like.

### Survival analysis using M2- and M12-like differentially expressed genes

**Figure 5:**
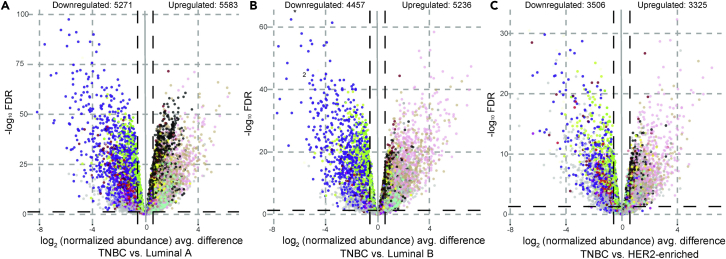
Volcano plots of differentially expressed genes (DEGs) in TNBC TNBC case group was compared with receptor-positive case groups Luminal A (A), Luminal B (B), and HER2-enriched BrCa (C). Log_2_ (fold change) for each comparison versus Benjamini-Hochberg FDR is plotted, and gene transcripts are colored by module membership (no module: light gray). DEGs were counted for >50% change (vertical cutoff lines at x = ±0.58) and FDR<5% in each individual comparison. See also Table S6. Full TNBC versus receptor-positive subtypes DEG list, related to Figure 5, Table S7. Top 20 differentially expressed genes in TNBC versus Luminal A BrCa for M2-like and M12-like modules, related to Figure 5, Table S8. Top 20 differentially expressed genes in TNBC versus HER2-enriched BrCa for M2-like and M12-like modules, related to Figure 5.

An unpaired two-tailed t test followed by Benjamini-Hochberg FDR estimation was conducted to compare differential expression among TNBC tumor subtype cases (N = 92) and the three receptor-positive subtypes: Luminal A (N = 226), Luminal B (N = 118), and HER2-enriched (N = 57). Statistical significance for counting differentially expressed genes was set to FDR<0.05. Volcano plots in Figure 5 report the number of genes upregulated and downregulated as well as differentially expressed between TNBC and each of the non-TNBC (Luminal A, Luminal B, and HER2-enriched) tumor groups. A complete list of differentially expressed genes (DEGs) (p < 0.0001) between TNBC and each non-TNBC tumor group can be found in Table S6, filtered for consistency of directional change and significance in all three pairwise comparisons of non-TNBC cases to the TNBC group to further control false positives (N = 1,518). The top 20 downregulated and upregulated genes for each of the four M2-like and five M12-like modules linked to TNBC are given in Tables S7 and S8. Table S7 has rank based on FDR for the Luminal A *versus* TNBC comparison, and Table S8 shows rank based on the comparison FDR for HER2-enriched *versus* TNBC subtypes.

**Figure 6:**
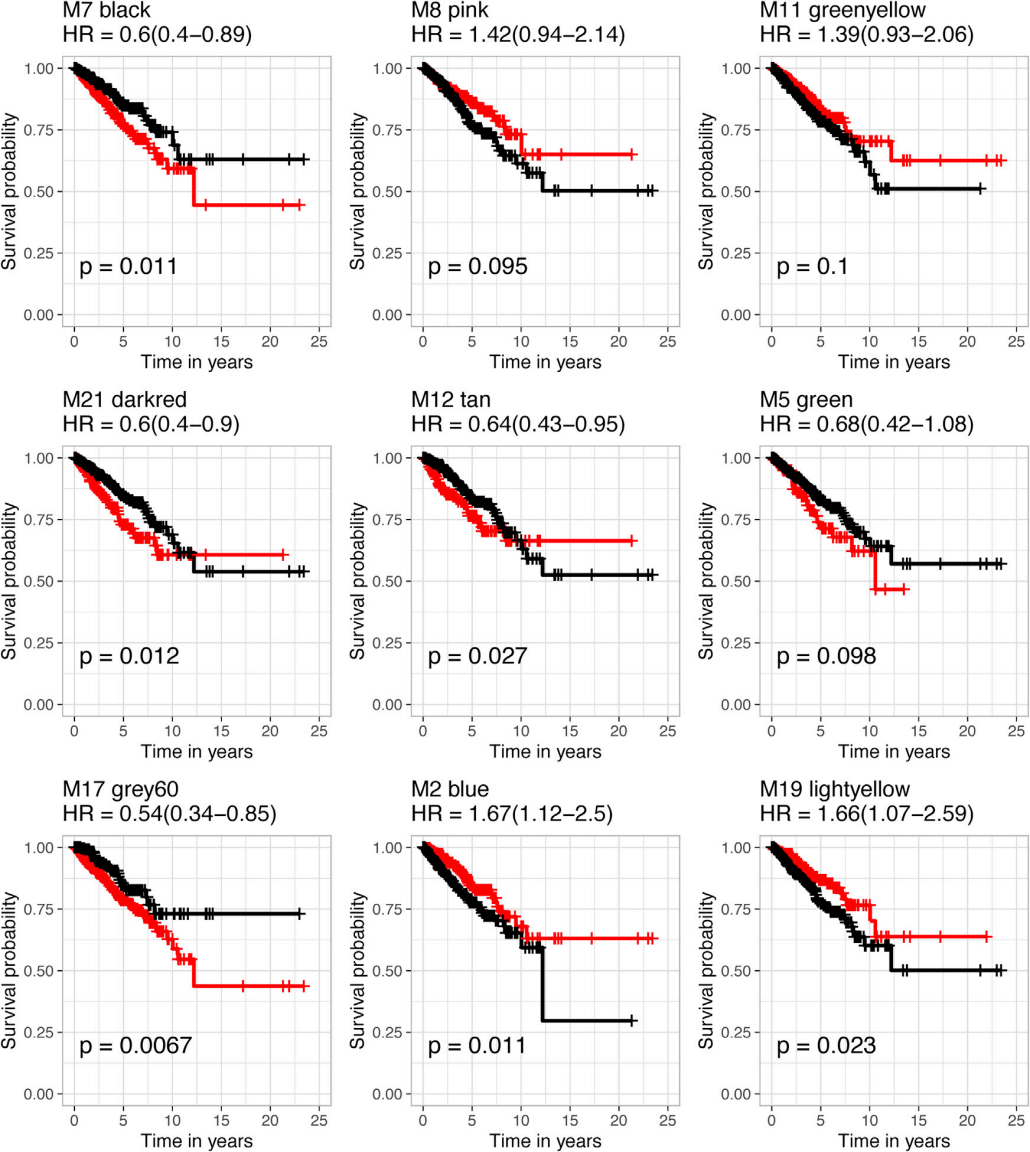
Survival analysis reveals association between M12-and M2-like module eigengene values and progression free interval time PFI in breast cancer Hazard ratios confirmed low expression of M12-like eigengene values, except M8, resulted in a significantly high likelihood of relapse, while high expression of M2-like modules, except M11, resulted in significantly less likelihood of relapse (left). The red line in each Kaplan-Meier plot represents survival of cases in the higher expression tier and the black line represents survival in cases with lower expression. A log rank (Mantel-Cox) test determined p values and hazard ratio (HR) scores. See also Figure S1 and Table 2.

After differential expression analysis, KM survival analysis (Gyorffy et al., 2010) nominated high- and low-expressed gene subsets that significantly distinguished patients dichotomized by survival propensity in the selected two groups of opposing module communities, namely, the aggressive TNBC modules (M7, M21, M17, M8, M12) and the non-TNBC modules (M2, M11, M5, and M19). Nine TNBC module MEs were analyzed to test the association of low *versus* high eigengene value with progression free interval (PFI) time (Figure 6), which is analogous to relapse-free survival (RFS) (Liu et al., 2018), in all BrCa subtypes of the TGCA RNA-seq data. A difference in PFI, higher survival with lower expression, was seen in all M12-like modules and a difference in PFI, higher survival with higher expression, was seen in all M2-like modules. However, M8 and M11 exhibited an association trend in the opposing direction respectively different from trends for the other M12-and M2-like modules. Although we performed the analysis across all 773 individuals, not just the 92 TNBC cases, this result suggests that gene coexpression in these two eigengenes is compensatory and not exacerbating TNBC poor prognosis. Hazard ratios (HRs) with 95% confidence intervals (CIs) confirm four of the five M12-like modules have relative differences in expression of coexpressed genes that coincides with the significant risk for the patients to relapse. HRs confirm three of the four M2-like modules have relative decreases in expression of coexpressed genes coinciding with significantly lower risk of relapse.

Following eigengene survival association, the top 20 TNBC DEGs in the M12 (upregulated) and M2 (downregulated) modules as ranked by log_2_-fold change in Table 2 were also analyzed by KM analysis to test the association of low *versus* high single-gene expression with RFS in all BrCa subtypes of an independently curated RNA-seq meta-analysis (Gyorffy et al., 2010). A difference in RFS, higher survival with lower expression, was seen in the M12-specific DEG hub gene *PSAT1* (p = 0.0042; HR = 1.87, CI = 1.21–2.89). A hub gene is a gene with a connectivity score kME = 0.6 or higher. *PSAT1* (kME 0.81) is the number 1 hub gene in the M12 module. In the opposing direction—higher survival with high expression—of M2 genes *TFF1* (p = 0.011; HR = 0.49, CI = 0.28–2.86) and *SCUBE2* (p = 0.00045; HR = 0.47, CI = 0.3–0.72) (Figure S1). *TFF1* is the top DEG in the M2 module with 256-fold higher expression in TNBC than HER2-enriched BrCa. *SCUBE2* (kME = 0.75) is the top differentially expressed hub gene in the M2 module. As expected, expression of *PSAT1* was highest in the TNBC subtype sample group, and expression of *TFF1* and *SCUBE2* was lowest for the TNBC group, each reaching a two-group Wilcoxon rank-sum test p < 2.2x10^−16^. Based on their association with survival, we selected 11 significant gene transcripts (6 from M2 and 5 from M12) as a seed list of potential high-performing BrCa prognostic signature genes (see the following text). Of the 20 M2-like DEGs, 10 genes have HRs that confirm patients are significantly less likely to relapse, whereas five of 21 M12-like DEGS have HRs that confirm patients are significantly more likely to relapse.

**Table 2:** Relapse-free survival analysis using BrCa RNA-Seq (N = 1,090) for Kaplan-Meier plots

DEGs TNBCversus Luminal A^b^							DEGs TNBCversus Luminal A^b^						
M2							M12						
Gene	WGCNA kMEblue	FDR	Log_2_ diff	Kaplan-Meier Log rank P	Direction^a^	Selected	Gene	WGCNA kMEtan	FDR	Log_2_ diff	Kaplan-Meier Log rank P	Direction^a^	Selected
**TFF1**	0.55	7.13E-52	-8.18	0.011	+	Yes	HORMAD1	0.54	2.46E-61	6.35	0.4	ns	
AGR3	0.61	9.16E-86	−7.61	0.0056	+	no probe	**ART3**	0.67	4.74E-64	6.19	**0.0035**	–	Yes
TFF3	0.56	2.87E-70	−7.14	0.034	+		LINC01956	0.64	5.79E-55	5.59	NA	NA	
SRARP	0.54	1.11E-47	−6.88	NA	NA		FOXCUT	0.78	9.44E-67	5.50	NA	NA	
**AGR2**	0.60	2.63E-71	−6.73	0.014	+	Yes	GABBR2	0.63	4.14E-42	5.23	0.069	ns	
ESR1^c^	0.83	6.05E-93	−6.38	0.01	+	∗∗	ZIC1	0.52	2.77E-37	4.99	0.097	ns	
**GP2**	0.48	6.38E-34	−5.94	0.0061	+	Yes	NKX1-2	0.75	1.06E-38	4.88	NA	NA	
CT62	0.69	6.94E-85	−5.92	0.0062	+	no probe	PRSS33	0.57	2.54E-23	4.68	**0.0035**	–	no probe
LINC00504	0.74	3.06E-98	−5.79	NA	NA		ENSG00000 179066	0.60	5.26E-55	4.38	NA	NA	
FOXA1	0.47	1.59E-90	−5.75	0.091	ns		ENSG00000 248538	0.63	3.82E-35	4.38	NA	NA	
POTEKP	0.66	8.69E-46	−5.70	NA	NA		GFRA3	0.52	1.33E-44	4.31	0.22	ns	
ENSG00000240800	0.53	4.35E-33	−5.62	NA	NA		NDUFB4P11	0.70	1.12E-40	4.24	NA	NA	
**ABCC8**	0.71	4.47E-49	−5.51	0.00056	+	Yes	SLC26A9	0.65	6.60E-39	4.12	0.061	ns	
ENSG00000235584	0.62	2.03E-33	−5.50	NA	NA		**FZD9**	0.64	5.05E-61	4.11	**0.0027**	–	Yes
TNRC18P1	0.67	3.82E-59	−5.50	NA	NA		**PSAT1**	0.81	2.39E-59	4.09	**0.0042**	–	Yes
SLC44A4	0.40	1.92E-71	−5.50	0.017	+		CASC8	0.64	4.83E-49	4.09	NA	NA	
**ERBB4**	0.70	2.00E-68	−5.47	0.008	+ (<5 yrs)	Yes	LINC01198	0.68	4.54E-28	4.09	0.29	ns	
**SCUBE2**	0.75	1.09E-65	−5.47	0.00045	+	Yes	**OPRK1**^d^	0.60	1.45E-27	3.95	0.35	ns	Yes∗∗∗
TTC6	0.62	1.63E-75	−5.43	4.30E-05	+	no probe	ABCA13	0.57	3.08E-29	3.82	0.3	ns	
LINC02568	0.65	3.90E-61	−5.28	NA	NA		**MARCO**	0.64	8.72E-34	3.81	**0.011**	–	Yes
							YBX1	0.73	3.18E-53	1.42	0.12	ns	
**Total M2 numerator genes (bold): 6**							**Total M12 Denominator Genes (bold): 5**						

KMplot.com-curated data for BrCa RNA-seq (Gyorffy et al., 2010) was used to perform survival analysis of gene transcript-dose effects on RFS in BrCa (all subtypes). Genes were nominated by Luminal A versus TNBC DEG FDR rankings (Table S7) of 20 or less with M2 (left) or M12 (right) membership. Genes with significant dose effect (log rank p value < 0.015) were nominated for inclusion in the subsequent first round of ROC analysis. See also Figures 6 and S1.

a Minus, low expression, high survival; plus, high expression, high survival.

b Consistent and significant for all 3 TNBC pairwise comparisons.

c Estrogen receptor excluded as known driver.

d OPRK1 K-M log rank p = 0.00056 for array data (−).

### Interaction analysis of M2-and M12-like modules confirms driver nodes

The coexpression networks of top ranked genes (kME = 0.6 or higher) for the M12-and M2-like modules were analyzed for mammary-gland-specific genetic and other interactions, which were assessed using the GIANT reference database (Wong et al., 2018). Other interactions between survival-linked genes for the top anticorrelated modules, M12 (*PSAT1* and *PRSS33*) and M2 (*TFF1* and *SCUBE2*) were also assessed. Target pair interaction scores for all M12- and M2-like top ranked hub genes were obtained via an adapted interface to the GIANT v2 database (Greene et al., 2015) and are reported in Table S9.

**Figure 7:**
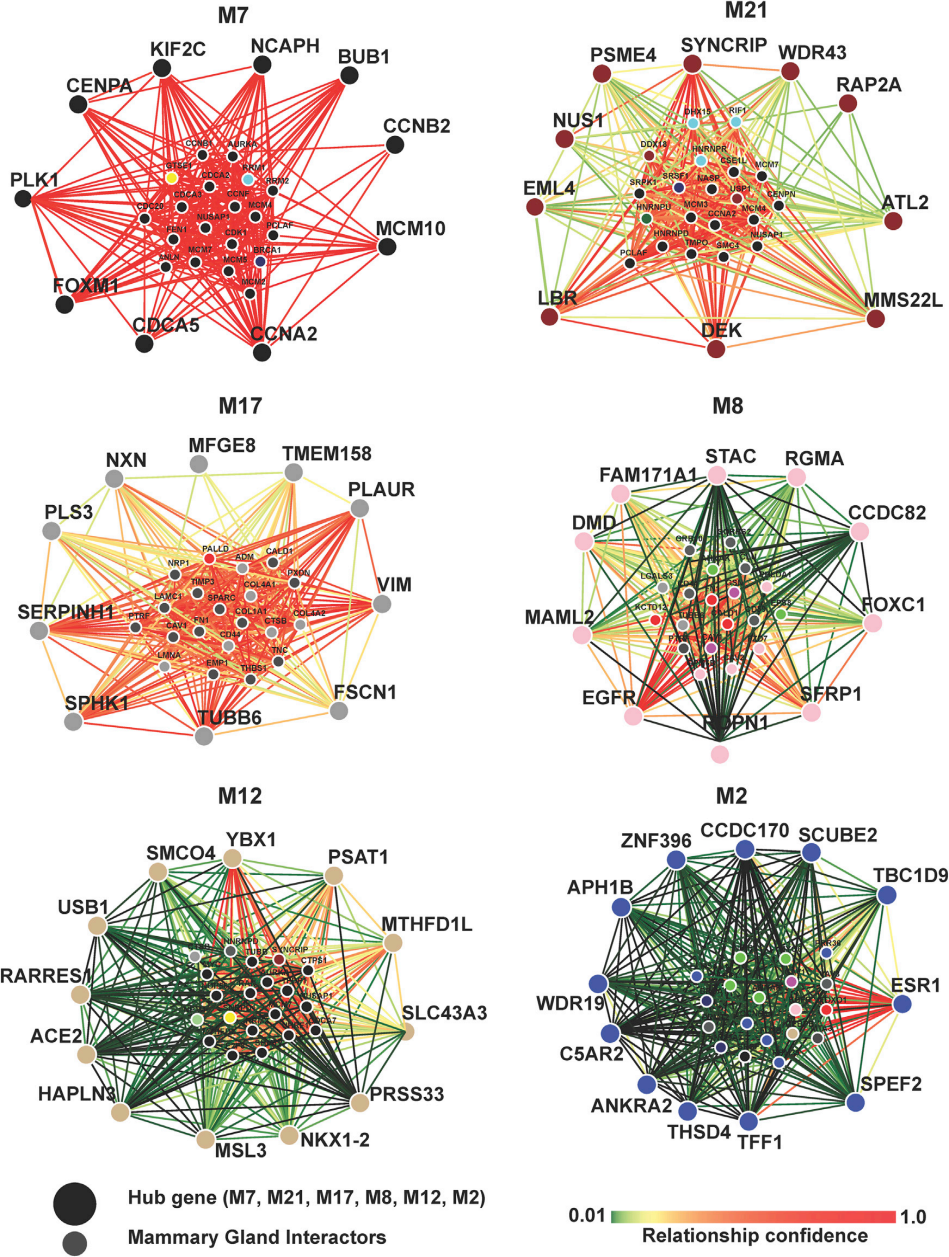
Genome-scale integrated analysis of gene networks in tissues (GIANT) identifies higher connectivity breast-tissue-specific interactions between survival-linked genes and module hub-implicated genes as well as hubs in M12-like and M2-like modules The heatmap scale of edge (interaction) colors represents the confidence of the predicted interactions based on mammary-gland-specific interactions. High confidence interactions are represented by red edges (see scale). Input for GIANT was restricted to the top ten hub genes for each module, plus nonhub survival-linked genes such as *PRSS33* (M12). Small nodes in the center of each network are implicated by the GIANT database network as the best connected to the hubs specified in mammary tissue, colored by their module membership. See also Figure S2 and Table S9.

Modules with higher connectivity breast-specific mammary interactions (M7, M21, M17, M8, M12, and M2) are displayed in Figure 7. The M12-like module, M7, with significant associations to biological processes such as the cell cycle, was the only M12-like module that had strong interactions between all ten M8 hub genes and GIANT breast-specific mammary implicated genes, including a validated BrCa biomarker, *BRCA1*. In addition, *EGFR* is higher expressed in TNBC versus non-TNBC cases and is a hub of M8 as indicated in both the kME table (Table S5, kMEpink = +0.841, ranked fourth in the module) and Figure 7. However, in Figure 6, dichotomization of the M8 eigengene into low- and high-expressing cases and comparison of progression-free interval as a proxy for survival in these two groups does not reach significance in the data analyzed. Overall, our results support that *EGFR* mRNA is elevated in TNBC, which is congruent with literature (Nielsen et al., 2004), but in the data examined as a whole across the 4 different BrCa subtypes, *EGFR* dichotomized high and low relative expression does not separate low from high progression-free interval or in summary no significant association between *EGFR* and our survival outcome, progression-free survival.

Three hub genes of M12, *PSAT1* (kME = 0.81), YBX1 (kME = 0.72), and *MTHFD1L* (kME = 0.72), are strongly interconnected in the M12 hub-derived network of breast tissue (Figure 7). *PSAT1* has the highest M12 kME and has significant mammary specific interactions, *YBX1* has the strongest edges in the network, and *MTHFD1L* had fewer strong interactions. Moreover, *PRSS33*, a nonhub M12 member (kME = 0.57) and significantly associated with BrCa survival, did not contribute to the nomination by GIANT of mammary network-implicated genes, having no significant mammary specific interactions among hubs of M12 or their most connected genetic interactors.

M2 was the module with significant breast-specific mammary gland interactions. Modules with lower connectivity, breast-specific mammary gland interactions are displayed in Figure S2. The M2 hub-derived breast interaction network is highly regulated by one gene, *ESR1* (Figure 7, bottom right panel), with kME = 0.82. *ESR1* has significant mammary-specific interactions with 13 genes including most notably, *BRCA1*, a validated risk gene for BrCa. Moreover, while the nonhub *TFF1* (kME = 0.54) and *SCUBE2* (kME = 0.75 but rank 34 among M2 hubs) integrated from the differentially expressed genes have a significant association of high expression with high RFS, neither was among those strongly connected within the tissue-specific hub-defined network. Interestingly, repeating GIANT with the same M2 hubs minus estrogen receptor (*ESR1*) implicated an entirely different set of interactors among the remaining hubs, with more balanced connectivity (*data not shown*). Notably, the M2 hub-derived GIANT network, *CEBPB* had high M2 hub connectivity but belonged to M12, thereby bridging the two anticorrelated modules. Such genes, known as bottleneck nodes, constitute an important conduit for the interchange of information between the different gene modules which they bridge, enriched as a class for genes essential for survival (Yu et al., 2007), and more often successfully targeted by drugs (Yao and Rzhetsky, 2008), elevating the importance of inclusion of this gene in subsequent analysis.

### ROC of survival prediction for integration of marker candidates, testing, and validation of a prognostic TNBC-specific biomarker ratio panel

These nominated transcripts based on RNA-seq, WGCNA, and KM analyses were assembled into prognostic indicator ratios, tested, and optimized by rounds of ROC analysis in an independent 1,234 array meta-analysis of BrCa (Györffy and Schäfer, 2009), including 180 TNBC cases (Karn et al., 2011) as a data set (traits provided in Table S10). Currently, treatment data for these patients is not available, thus survival is irrespective of treatment. Follow-up duration for this cohort of patients was 10 years for all BrCa subtypes and 7.5 years specifically for patients with TNBC. Our goal was to use this independent aggregate data from an orthogonal measurement technique to test combinatorial gene expression ratios made up of candidate genes discovered as described in aforementioned results, based on the framework of combining M2-and M12-like genes of anticorrelated coexpression transcriptome network modules identified in our curated RNA-seq meta-analysis. We hypothesized that negative correlation of gene expression profiles with high correlation to disease traits including survival could drive discriminatory difference in a ratio of genes (a) with numerator genes having expression positively correlated with survival, divided by (b) denominator genes that synergize or act together negatively to impact mechanisms of survival.

**Figure 8:**
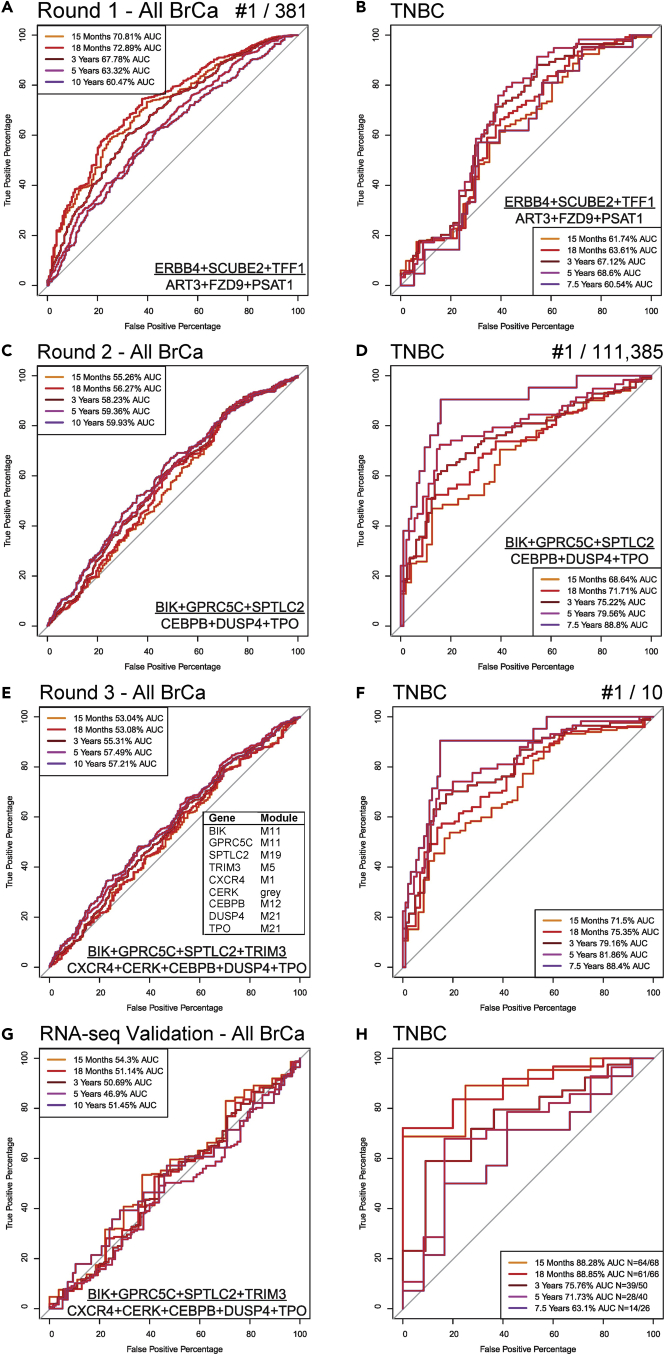
Top-performing survival predictors by ROC AUC ROC curves predicting RFS were generated on combinatorial ratios of M2(-like)/M12(-like) nominated genes, first using microarray-measured normalized transcript abundances in N = 1,234 BrCa cases with RFS traits. In round 1, transcripts consisting mainly of M2 and M12 hubs nominated by Kaplan-Meier distinction of RFS (Table 2) via their levels in independent RNA-Seq were tested in 381 combinatorial ratios for RFS prediction using all microarray BrCa cases (top performer shown in A), and only TNBC cases for the same normalized transcript abundance ratio (B). Round 2 added nominations of transcripts from M2-like and M12-like DEGs, as well as *CEBPB* and other genes from the breast tissue gene interaction network, producing the top ranked ROC curve with the best AUC(s) obtained using only TNBC microarrays, shown first for all BrCa (C), or TNBC only (D). Round 3 ROC tested whether additions of known BrCa genes in pathway(s) implicated by round 2 top predictor genes might further improve AUC, and the top performer in TNBC was plotted for all BrCa (E) and TNBC specific cases (F). Finally, the same ratio was calculated using the 773 RNA-Seq TCGA case abundances used for network building, normalized like the array data, and validated by ROC analysis for all BrCa (G) and TNBC (H). See also Figure S3 and Table S10. Microarray case traits used for ROC testing (N = 1,234), related to Figure 8, Table S11. Round 1 ROC survival predictor gene expression ratio using Affy Arrays (N=1,234), related to Figure 8 and Table 3, Table S12. Round 2 ROC survival predictor gene expression ratio using Affy arrays (N=1,234), related to Figure 8 and Table 3.

The 11 DEGs highlighted with the most significant dose-effects on survival in KM plots (Table 2), available for testing in the array data, were nominated for a first round of combinatorial tests for their potential to contribute to a multigene equal-weight ratio for prediction of BrCa survival. M2 (or M2-like) module member relative abundance was divided by M12 (or M12-like) module member abundance within sample, so that opposing changes within a sample would amplify sensitivity for survival prediction, and specificity of the prediction across many samples could be tested by the ROC of survival prediction for that ratio, given known survival status of each patient during extended follow-up. If a TNBC sample expressed M2 transcripts with a role in survival at elevated levels, this might counter the general TNBC-associated direction of change and poor TNBC prognosis. In addition, if those transcripts are mechanistically involved in slowing cancer progression, they might improve survival across BrCa cases in general. Therefore, high relative expression of M2 genes should represent high survival odds, while low relative expression of M12 genes should represent higher survival odds in BrCa in general. Therefore, a larger ratio predicts better survival prognosis in this model. The complete set of 11 genes combined and 380 additional combinations covering all possible 1:1, 2:2, and 3:3 gene ratios were tested for prediction of RFS using AUC as the readout; all combinations were >50% AUC on average across calculations for 5 specific time points in BrCa (Table S11), supporting the use of M2/M12(-like) gene ratios for survival prediction. The best combination for the full cohort of 1,234 BrCa cases of mixed subtype (Figure 8A) had a maximum AUC of 72.9% for prediction of survival at 18 months, though Table S6 demonstrates that many combinations were similar. The top ratio was (*ERBB4* + *SCUBE2* + *TFF1*/*ART3* + *FZD9* + *PSAT1*), outperforming the naive ratio of all 11 transcripts by only 1.1%. Prediction of survival in the subset of 180 TNBC array cases was highest for this combination at 5 years, but only with a 68.6% AUC (Figures 8B), 4.7% higher than the 11-gene ratio. Interestingly, the 2-gene combination *ERBB4*/*FZD9* was the best 5-year TNBC survival predictor among the 381 combinations, with a 76.0% AUC. This first round of ROC tests suggests different combinations of genes play more of a role in TNBC specifically than in BrCa in general.

We expanded our test to consider transcript abundance ratios of genes implicated by genetic interactions, and M2-or M12-like module members are also highly ranked DEGs in TNBC, in particular compared with the next most severe BrCa subtype, HER2-enriched (Table S8). Of the 184 genes in the table, for the numerator we selected the top 14 M11 genes, the top 5 from M19 plus a lower ranked M19 DEG, *NCAM2*, as well as *ERBB4* and *SCUBE2* from ROC round 1. Two additional genes implicated by the interaction network of M2 hubs that were also DEGs (*AHNAK* of M5 and *VAV3* of M2) were included as well, representing 24 total candidate numerator genes from M2-like modules. For the round 2 ROC indicator ratio denominator candidates, we selected *CEBPB* of M12 implicated by GIANT as connected to the M2 hub network and *CEBPG* of M7 as a negative control not implicated by our integrated analysis, but still in an M12-like module. DEGs implicated by the M12 hub interaction network were *CDCA7, CTPS1*, and *MCM7*. *ART3*, *FZD9*, and *PSAT1* carried over from the first round, and the top 12 M8 DEGs plus the top 14 M21 DEGs in Table S8 rounded out a total of 34 candidates in the M12-like genes of the denominator.

We expected that the 111,385 complete combinations (devised again as 1:1, 2:2, and 3:3 gene ratios), would be enriched for higher AUCs in prediction of TNBC-specific survival compared to overall BrCa because HER2-enriched versus TNBC DEGs (Table S8) are included in addition to the top nominations from survival analysis (Table 2) and the tissue interaction networks (Figure 7). This was indeed the case (Table S12), with the average AUC of the top 10 combinations (all 3:3 ratios) at 79.0% for predicting survival at 5 years, compared with 70.8% in round 1. On the other hand, in round 2, the best all-subtype BrCa prognostication ranked by maximum overall BrCa predictor AUC at any time point but averaged for the top 10 at the 5-year time point was <64.0%, though better at earlier time points, not exceeding 73.1% for any combination at any time point. Moreover, the best performing TNBC predictor obtained by this relatively unbiased survey of candidates performed poorly for overall BrCa (AUC_max_ < 60.0%, Figure 8C), while exceeding 88% for TNBC 7.5-year survival and 79.6% at 5 years (Figure 8D). The top-ranked ratio consisted of markers distinct from round 1, despite 5 of 6 of the round 1 top performing ratio genes being tested: (*BIK* + *GPRC5C* + *SPTLC2*/*CEBPB* + *DUSP4* + *TPO*). These 6 genes were enriched among most of the top 100 performing combinations (Table S12), suggesting robustness of the approach.

To perform a third and final round of ROC tests, we decided to examine peer-reviewed literature for the aforementioned 6 genes and determine logical candidates to augment the ratio by their known involvement in both the biological pathways already implicated and with known roles in BrCa if not TNBC survival prognosis. We are thereby integrating verified biological knowledge into the largely unbiased process of gene nomination and testing, which improves prospects of reproducibility and general applicability of our biomarker ratio in principle. We also nominated *TRIM3*, found in 46 of the top 100 TNBC predictors, but only appearing in 22.6% of all round 2 combinations; it is found in place of *GPRC5C* as the only change in the second-best round 2 TNBC survival predictor, which outperforms 5-year survival prediction for TNBC of the top indicator at 83.3% AUC versus <80.0% (Table S12).

Sphingolipid pathway genes related to BrCa (implicated by *SPTLC2*) in the literature included long-chain ceramide synthase *CERS4*/*LASS4* (connected to round 1 candidate *SCUBE2* by opposing its role in SHH shedding (Gencer et al., 2017; Tsai et al., 2009), where this developmental morphogen being reexpressed in BrCa has been specifically implicated in its migration and invasiveness (Riaz et al., 2019). In addition, ceramide kinase *CERK* was implicated in BrCa metastatic migration (Schwalm et al., 2020). Chemokine receptor signaling was of interest, and we found *CXCR4* and *CXCR5* comentioned with *GPRC5C* in a review of vascular development dysregulation relevant to cancer (De Francesco et al., 2017). Angiogenesis is a requirement for larger tumor growth. We chose to test *CXCR4* because of its higher kME among CXCRs in the weakly M12-like M1, which was the only module harboring this class of genes including *CXCR5* (Table S5). Adding *CERS4*/*LASS4* to the numerator did not improve test ROC AUC (*data not shown*), but the remaining new genes (numerator: *TRIM3*; denominator: *CXCR4* and *CERK*) in addition to members of the round 2 top-performing ratio did raise average AUC for the 5 time points tested in the top TNBC predictor from 76.8% to 79.3%, indicating that literature-informed improvements to the predictor are possible. The final 9-gene prognostic indicator was ineffective for prediction of overall BrCa survival (Figure 8E), compared with TNBC (Figure 8F), for which it was highly specific. Significance of improvement in ROC curves was calculated for relevant pairwise comparisons, along with predictor significance of AUC and 95% CIs, and these are provided in Table 3. Citations across cancer literature were mined by gene symbol, submitting genes from the round 1 top performing ratio and all candidates for rounds 2 and 3 using OncoScore (Piazza et al., 2017) to determine known relevance to cancer, and we found that most of the genes were represented with a significant score (Figure S3).

**Table 3:** ROC analysis Statistics for top performing transcript abundance ratios

Round	Survival predictor	15-month TNBC	18-month TNBC	3-year TNBC	5-year TNBC	7.5-year TNBC
**1**	ERBB4+NCAM2+SCUBE2ART3+ FZD9+PSAT1	p=0.0023 CI 0.5399-0.7373	p=0.00026 CI 0.5681-0.7522	p=1.5e-05 CI 0.6042-0.7723	p=2.1e-05 CI 0.6158-0.7838	p=0.024 CI 0.5267-0.7499
**2**	BIK+GPRC5C+SPTLC2CEBPB+ DUSP4+TPO	p=6.7e-05 CI 0.6025-0.7703	p=1.3e-06 CI 0.641-0.7932	p=1.1e-08 CI 0.6769-0.8275	p=6.9e-10 CI 0.7168-0.8744	p=1.5e-08 CI 0.8058-0.9703
**3**	BIK+GPRC5C+SPTLC2+TRIM3 CXCR4+CERK+ CEBPB+DUSP4+TPO	p=5.3e-06 CI 0.6311-0.7989	p=2.0e-08 CI 0.6791-0.828	p=5.1e-11 CI 0.7224-0.8608	p=3.3e-11 CI 0.7483-0.8889	p=2.1e-08 CI 0.8079-0.9601
	**Rounds compared for improvement (2,000 bootstrap p)**					
	**Round 3 versus Round 1**	0.13	0.059	**0.032**	**0.016**	**0.00013**
	**Round 2 versus Round 1**	0.23	0.17	0.13	**0.044**	**0.00018**
	**Round 3 versus Round 2**	0.32	0.26	0.22	0.33	0.53

**Round**	**Survival predictor**	**15-month BrCa**	**18-month BrCa**	**3-year BrCa**	**5-year BrCa**	**10-year BrCa**
**1**	ERBB4+NCAM2+SCUBE2 ART3+FZD9+PSAT1	p=4.6e-16 CI 0.6665-0.756	p=9.8e-22 CI 0.6901-0.7711	p=6.3e-20 CI 0.6432-0.7147	p=3.9e-14 CI 0.6023-0.6712	p=5.9e-08 CI 0.5722-0.6535
**2**	BIK+GPRC5C+SPTLC2CEBPB+ DUSP4+TPO	p=0.023 CI 0.4973-0.6078	p=0.0048 CI 0.5124-0.6131	p=1.5e-05 CI 0.5425-0.622	p=1.6e-07 CI 0.5578-0.6293	p=1.6e-06 CI 0.5587-0.6398
**3**	BIK+GPRC5C+SPTLC2+TRIM3CXCR4+CERK+ CEBPB+DUSP4+TPO	p=0.12 CI 0.4776-0.5833	p=0.10 CI 0.4821-0.5796	p=0.0036 CI 0.5136-0.5926	p=2.1e-05 CI 0.5390-0.6107	p=0.00036 CI 0.5309-0.6134
	**Rounds compared for improvement (2,000 bootstrap p)**					
	**Round 1 versus Round 2**	**5.40E-06**	**1.10E-07**	**3.30E-04**	**0.044**	0.32
	**Round 1 versus Round 3**	**1.60E-07**	**2.30E-10**	**2.10E-06**	**0.0063**	0.081

Significance and 95% CI are provided for each of the top predictors, in ROC analysis of either TNBC or all BrCa at the five selected timepoints. Comparisons of top predictor ROC curves testing for significance of improvement between rounds of ROC tests are also provided, with significant comparison p values bolded. See also Figure 8 and Tables S10–S12.

To validate our round 3 ROC test of the 9-gene predictor, we calculated the 9-gene ratio in the TCGA RNA-seq quantified samples. Data were subjected to sample-wise normalization identical to the array normalization performed. ROC curves were similar to the ones produced from array-measured samples for both BrCa RFS prediction (Figure 8G) and for TNBC (Figure 8H), where this ratio was only effective for prediction of survival in TNBC. Thus, our ratio predictor of survival determined in array data from independent BrCa cases with gene nominations based on the network built on RNA-seq data validates in the RNA-seq data, indicating reproducibility and robustness of the predictor.

## Discussion

### Complex interaction of hallmark cancer process genes affects cancer survival

A systems biology pipeline (Figure 1) was used to integrate large-scale BrCa transcriptomes, to assess RNA coexpression and to identify gene products influencing survival of patients with BrCa, with emphasis on TNBC, a heterogeneous BrCa subtype with the worst prognosis and fewest specific treatments. While the classical subtype according to representative markers ER, PR, and HER2 for TNBC are well known, the genomic characteristics of TNBC are more complex than expected. Among previous classification studies, six intrinsic subtypes of TNBC have been identified (basal-like [BL], androgen receptor [AR], mesenchymal [M], and immune). These intrinsic TNBC subtypes include two BL subtypes, one with increased cellular proliferation and response to DNA damage response (BL1, 10%) and another with high growth factor signaling with myoepithelial markers (BL2, 20%); two M subtypes associated with cell differentiation and growth factor signaling (M, 20% and MSL, 10%, respectively); an immunomodulatory (IM, 20%) type enriched with immune cell processes; and a luminal androgen subtype characterized by hormone signaling mediated by androgen receptor (LAR, 10%) (Lehmann et al., 2011, 2016; Burstein et al., 2015).

WGCNA identified prototypic module communities with opposing correlation to coordinated gene expression changes in TNBC, which were a rich source of survival driver genes. We homed-in on hubs of these modules having higher connectivity in breast-tissue-specific networks, which also implicated interesting characteristic genes known to drive BrCa including a bottleneck, *CEBPB*. Many DEGs from these modules were highly associated with survival by KM survival analysis. All the aforementioned results were fed into tests of combinatorial gene expression ratio by ROC analysis (Figure 8). We found that initially promising genes linked to BrCa survival and based on some of the most apparent DEGs (by fold change) also fitting the coexpressed, anticorrelated framework of selection criteria underperformed in predicting survival in TNBC versus BrCa in general. However, adding to the nomination list additional genes from neighboring, yet distinct M2-like and M12-like transcript communities, biasing these choices by different criteria for top-ranking DEGs with very low FDR from the comparison of TNBC to HER2-enriched BrCa subtypes, resulted in better performance for prediction of TNBC survival among the top-ranked ROC curves. Among the genes performing best, hallmark cancer processes were implicated as discussed in the following text. Therefore, to further augment performance and to cement reproducibility of our predictor ratio, we mined the literature for definitive work demonstrating the involvement of pathway-related genes on BrCa, if not TNBC survival, and prediction AUC for the top-performing ratios indeed increased.

The hallmark processes of cancer we identified as relevant to TNBC via our relatively unbiased nomination and testing for predictive linkage between gene expression and survival include genes such as *BIK*, encoding a protein that balances proapoptotic and antiapoptotic signaling roles, and key sphingolipid metabolism enzymes that can catalyze interconversion of a lipid class that functions with anticancer propensity into lipid species that promote cancer, each having effects on cellular motility, differentiation, apoptosis, inflammation/immunity, and angiogenesis. These lipids, and their sphingolipid pathway enzymes, are appreciated to be dysregulated in numerous cancers with potential as drug targets (Ryland et al., 2011). Interestingly, the initial hit in the pathway, serine palmitoyl transferase long-chain base complex subunit *SPTLC2* has a low cancer-literature-relevance score (Figure S3), but catalyzes the rate-limiting step controlling flux of molecules into the sphingolipid pool subject to transformation to downstream products in subsequent steps of the pathway, such as ceramide synthesis by *CERS4*, or ceramide-1-phosphate production by *CERK*, which switch the active function of their products relative to substrates, sphingosine, and ceramide, respectively.

Angiogenesis-specific gene function was also implicated by the M2-like transcript abundance of *GPRC5C*, identified as a gene suppressed by estrogen receptor activation and capable of slowing MCF-7 growth (Yamaga et al., 2014), and a close paralog of *GPRC5* family genes *GPRC5A* and *GPRC5B*, with more established roles in cancer to date (Acquafreda et al., 2009; De Francesco et al., 2017; Hirabayashi and Kim, 2020), including dysregulation of ceramide production in a *GPRC5B*-deficient model (Kim et al., 2018). *GPRC5C* is only yet established to function in maintaining relatively higher blood pH, which may have an effect on endothelial proliferation capacity (Faes et al., 2016). *CXCR4*, nominated from literature comention with *GPRC5C* (De Francesco et al., 2017), has roles in immunity and a tumor microenvironment in addition to angiogenesis.

Interestingly, elevated *CXCR4* promoting a conducive tumor microenvironment through upregulation of stress activated kinase signaling is consistent with a weak M12-like signature in our network and with better survival when expressed less abundantly (Chatterjee et al., 2014). *MAPK1* and 2, *p38*, *JNK*, and other stress-activated kinases are also regulated by *DUSP4* (Mazumdar et al., 2016), which we took to be M12-like based on module assignment to M21. But on examination, it is not well-assigned owing to discordance of dissimilarity (directly used in WGCNA for assignment) and kME, which implicates it as an M19 (M2-like) member (kME = 0.54). Indeed, *DUSP4* was reported to be downregulated in TNBC (Mazumdar et al., 2016). We subsequently tested ratios with *DUSP4* and 1/*DUSP4* in the numerator for improving ROC AUC to no avail (*data not shown*). A potential explanation for the positive *DUSP4* contribution to the predictive ratio when offside in the denominator, compared with where its network membership would suggest *DUSP4* has a more complex biological role. The loss in TNBC of *DUSP4* could be a downstream effect of other expression changes in TNBC that has negative correlation to patient survival but with no role affecting survival or dual roles including one that compensates for the other in TNBC. Elucidation of these possibilities requires further study. Regardless, both *DUSP4* (Saigusa et al., 2013) and *TRIM3* (Huang et al., 2017) on opposite sides of our 9-gene ratio are suppressors of metastasis of hepatic cancer, suggesting tissue-specific if not pan-cancer roles in survival.

*CEBPB*, the high-information-load bottleneck node between M12 and M2, promotes inflammation-mediated metastasis in BrCa (Kurzejamska et al., 2014). The aforementioned 8 genes of the ROC round 3 survival predictor ratio are shown in Figure 1 (Venn diagram at lower left) with their overlapping roles in 5 hallmark cancer-related processes indicated. The remaining gene of the 9, thyroid peroxidase (*TPO*), has a less established role in cancer with a low cancer-literature-relevance score (Piazza et al., 2017) of 22.0, but it has been shown that autoantibodies to *TPO* predict BrCa risk (Tosovic et al., 2012). In total, TNBC survival prediction capability of our 9-gene equal-weight ratio was bolstered by selection of DEGs within two anticorrelated sets of modules of the network tied to BrCa survival and specific shifts by subtype. The ability of these genes to both encode the survival risk of patients and integrate hallmark cancer processes in TNBC nominates them as linchpins of TNBC survival. We further showed that the BrCa network is robust, demonstrating that the nomination framework applied from the RNA-seq-derived network to predictions in array data validates in RNA-seq data.

### Focus on DEGs of only M12 and M2 misses linchpin TNBC survival genes, but finds genes with well-studied roles in BrCa survival

All the gene ratios tested in round 1 performed better (or similar) for all BrCa compared with TNBC survival prediction. Moreover, the previously nominated linchpin survival genes of top-performing predictor combinations refined in ROC analysis rounds 2 and 3 in our pipeline outperformed genes in the naively informed round 1 predictor. This is attributed to more refined parameters for DEGs in later rounds compared with DEGs only in M2 and M12 and with the largest fold changes (of the TNBC versus Luminal A comparison) in the data set for round 1. Top DEGs by fold change (upregulated consistently in TNBC compared with each non-TNBC group) included *PSAT1*, an M12 hub (Table S6). *PSAT1* encodes phosphoserine aminotransferase. Selective loss of *PSAT1* suppresses migration, invasion, and experimental metastasis in TNBC (Metcalf et al., 2020). *PSAT1* catalyzes serine biosynthesis, where serine is required for several anabolic processes, such as protein, nucleic acid, and lipid synthesis, including for the initial step of *de novo* sphingosine production. Because metabolic processes are reprogrammed in cancer to promote growth and proliferation, it is not surprising that modified amino acid metabolism was overrepresented as an M12-specific biological process (Table 1). Another enzyme of the serine synthesis pathway found in M12 was *PHGDH* (kME_tan_ = 0.63), encoding 3-phosphoglycerate dehydrogenase. Overexpression of *PHGDH* has been reported in BrCa tumors and cell lines (Mullarky et al., 2016). Notably in TNBC, amplification and overexpression of *PHGDH* is associated with aggressive disease (Locasale et al., 2011; Possemato et al., 2011; Pollari et al., 2011). Mullarky et al. demonstrated that BrCa cell lines intrinsically overexpressing *PHGDH* are uniquely sensitive to its knockdown, whereas others are insensitive, suggesting that *PHGDH* inhibitors may have cancer cell survival in their crosshairs.

M2 downregulation in TNBC (Figure 3) was evident for M2 genes *TFF1* and *SCUBE2*, and their transcript levels also predict BrCa survival (Figure S1 and Table 2), as previously reported for *TFF1*, with established roles in the inhibition of proliferation, migration, and invasion of BrCa cells *in vivo* (Yi et al., 2020). *TFF1* is a secreted protein normally expressed in gastrointestinal mucosa, with transcriptional regulation by *ESR1* and 2 (Pelden et al., 2013). It is well-studied in relation to cancer (Figure S3, *TFF1* OncoScore = 80) and has been proposed as a biomarker for breast and other cancers (Wang et al., 2018; Yi et al., 2020; Yusufu et al., 2019; Klett et al., 2018). *TFF1* gene products were elevated in ER^+^ and PR^+^ BrCa (Yi et al., 2020; Elnagdy et al., 2018), whereas they are downregulated in TNBC (Yi et al., 2020). Indeed, *TFF1* levels correlate with *ESR1* and other transcription factors of M2, namely *GATA3*, *FOXA1*, and *MYB* (Table S5), supporting *TFF1* regulation by *ESR1* in our transcriptomic coexpression network. Given the aforemnentioned information, loss of M2 hub *ESR1* would be a driver of *TFF1* downregulation in TNBC and of increased cancer aggression. Enhancing expression or activity of *ESR1* or other M2 transcription factors could be a therapeutic approach in TNBC.

*SCUBE2* is a lipid-binding protein and coreceptor for *VEGFR2* to mediate angiogenesis. Guan et al. reported high *SCUBE2* expression associated with increased disease-free interval and good prognosis in BrCa, also finding ethnicity-specific differences in the expression of *SCUBE2* (Guan et al., 2020). *SCUBE2* is downregulated in invasive BrCa but overexpressed by breast cancer stem cells (BCSCs) (Chen et al., 2018). Possibly, the true prognostic value of *SCUBE2* is not observed in tissue biopsies because BCSCs are rare cells compared with differentiated cells with lower expression of *SCUBE2*. TNBC has a higher proportion of BCSCs compared with other BrCa subtypes (Honeth et al., 2008), promoting progression through proliferation, migration to metastatic sites, and therapy resistance. *SCUBE2*-expressing TNBC BCSCs also increase NOTCH signaling, epithelial to mesenchymal transition, and chemoresistance. Patients with TNBC with African ancestry have a higher prevalence of tumors expressing stem cell markers such as CD44, which elevates the expression of *SCUBE2* (Jiagge et al., 2018), thus *SCUBE2* could serve as a therapeutic target for African-American patients with TNBC. Our study and those cited support *SCUBE2* and *TFF1* links to estrogen signaling, but further *in vitro* studies are necessary to determine how these genes are connected in TNBC signaling (Fernandez-Ramires et al., 2009; Smith et al., 2008). Given the additional role of *SCUBE2* in SHH signaling (Tsai et al., 2009) and SHH signaling downregulation by ceramide (Gencer et al., 2017), a closer look at interactions of *SCUBE2* with ceramides and linchpin survival genes affecting sphingolipid balance is also warranted.

We noticed that M12 hub genes *PSAT1*, *YBX1*, and *MTHFD1L* with high confidence interactions to the nucleic acid and DNA metabolism module M7 (Figure 7; *M7 nodes, black*), plus *PHGDH*, have been comentioned in previous work finding gross dysregulation of the proteome in model cell lines for *HPRT1* deficiency (Dammer et al., 2015). Hypergeometric overlap testing was performed for overrepresentation of 1,055 *HPRT1* deficiency-dysregulated gene products among M2-like or M12-like module hubs (kME≥0.70, n = 552) in the BrCa network, relative to the hubs of other modules (n = 1,209). Of 88 *HPRT1*-linked hub proteins, 38 were hubs in the 9 BrCa network modules of core interest to TNBC (p = 0.0066; OR = 1.79), indicating significant enrichment within hubs of our TNBC survival-linked network communities. Twenty of the 38 were M7 hubs, and M7 harbors *HPRT1* as a bottleneck (kME_black_ = 0.561; kME_yellow_ = 0.556). As an M12-like module, the M7 DNA synthesis module is elevated in TNBC, consistent with the study by Sedano, et al., which found *HPRT1* elevation particularly in TNBC, and association with poor BrCa outcomes (Sedano et al., 2020). The unusual genetic interaction network of *HPRT1*, as seen in both proteomics and RNA, makes this region of the network a rich hunting ground for mechanisms of cancer progression. We found that connectivity among the M12-like community of modules is relevant to TNBC survival, which supported our decision to expand our search over the network landscape to all M2-like and M12-like modules when we homed-in on TNBC linchpin survival genes.

Overall, ROC analysis helped us establish a predictor of progression free survival in BrCa without literature or other selection bias. We discuss how the unique combination of implicated pathways relating to BrCa survival overlap with known hallmark pathways of cancer. We predict that therapies targeting multiple of the 9 implicated targets and their respective pathways via modulation of expression (or function) of these key driver genes will improve treatment outcomes through synergistic effects on mechanistically distinct molecular pathways influencing BrCa progression, relapse, and survival.

### Limitations of the study

The identification of candidate biomarker genes from high throughput abundance data for the use of therapeutic prediction is subject to defects by way of analysis parameters. Often, the result is unreproducible by complementary methods, limiting the power of translating these gene lists into clinical biomarkers and therapeutic targets. The workflow used in this study addressed this problem by integrating systems biology approaches to discover nominated genes and unbiasedly testing their combinations, representative of biological interaction possibilities, arriving at multigene survival indicators well-supported by prior biological knowledge, also demonstrating that existing literature can augment the predictivity of a nominated biomarker list. Text mining in future integrative systems biology analysis pipelines such as ours, amenable to automation, would be valuable.

We sampled a small region of a vast network landscape. Ideally, all hubs, hub interactors, bottlenecks, and DEGs of M2-and M12-like modules would have been tested for potential to contribute to the combinatorial predictive biomarker. Although logical as the choice of genes likely to differentiate patient survival, examining the top 20 or fewer DEGs of the M2(-like) and M12(-like) modules for survival and ROC analyses probably introduced bias toward better-studied genes, while leaving many details of the network landscape unexplored. To optimize predictors of TNBC survival, distinct subsets of DEGs were nominated, but changing the nomination criteria will allow for application of the analysis pipeline to other subtypes or BrCa in general. The computational demands of ROC testing of all gene combinations in an expanded landscape search would explode owing to the number of potential combinations to test. With only 24 numerator and 34 denominator candidates, round 2 of ROC analysis already tested more than100,000 combinations. This also could be viewed as a multiple testing problem, despite the large number of biological measurements involved. Machine learning might be used to optimize prescreen of small-number combinations before assembling them into more complex gene ratios. Indeed, machine learning approaches for BrCa and TNBC prognostic marker panel definition are gaining traction (Alsaleem et al., 2020). Weights for gene contributions to combinatorial predictors are often inferred by linear regression (Liu et al., 2019). We considered all combinations with equal gene weight after sample-wise normalization, which affected underlying biological functional interactions driving the ratio's sensitivity and specificity. Future work may explore the benefits of integrating both unweighted and weighted combinatorial predictors.

Our ROC analysis validating array-based results in RNA-seq data used corrected and complete traits for the TCGA samples (Liu et al., 2018). Nonetheless, the statistical power of the validation ROC analysis was limited by an unusual imbalance of outcomes in the cases with sufficient follow-up time. Twenty-three of the 92 TCGA TNBC cases were tumor-free before 15 months, at their last follow-up. This highlights the possible impact of therapies on candidate gene linkage to survival outcomes, hiding markers of naive BrCa prognosis already targeted by therapy. Because we chose to focus on survival of patients with TNBC, the biomarkers we have found are likely poorly engaged by current treatments. Biomarkers relying on bulk tissue biopsy also suffer from an inability to distinguish rarer cell type contributions to gene product abundances and prognosis. Integrated systems biology pipelines examining single-cell RNA-seq data therefore hold promise to distinguish BCSC and other rare but important cell-type contributions to BrCa mortality.

### Conclusion

This study successfully leverages coexpression and interaction networks of invasive tumors of the four BrCa subtypes in large transcriptomic cohorts, overlapping this structure with differential expression, nominating candidate biomarker genes tested for combinatorial functional interactions that influence survival of patients with TNBC. It also demonstrates that hub status is insufficient to assign genes as key drivers of causality and directionality in WGCNA networks. The combinations performing best comprised linchpin survival genes, representing hallmark cancer processes that involve sphingolipid metabolism, regulation of apoptosis, proteostasis, angiogenesis, and metastasis propensity. Therefore, our top survival-related genes from ROC ranking are generally already well-established, but their specific combination here implicates unappreciated functional interactions in BrCa remaining to be fully explored. Thus, our network serves as a resource for the research community. The networks identified for BrCa also remain to be leveraged by systems pharmacology, which may focus on therapies targeting the functions of gene combinations that normalize the profile of entire survival-associated network modules. Finally, this analysis pipeline holds promise for broader application to other disease-specific tissue-level transcriptomic and proteomic networks.

### Resource availability

#### Lead contact

Further information and requests for resources and reagents should be directed to and will be fulfilled by the lead contact, Dr. James W Lillard, Jr (jlillard@msm.edu).

#### Materials availability

Materials used or generated in this study will be available upon reasonable request, and a material transfer agreement may be required.

#### Data and code availability

All data are available from the corresponding author upon reasonable request.

## Methods

All methods can be found in the accompanying transparent methods supplemental file.

## Supplemental information

Document S1. Transparent methods and Figures S1–S4

Table S1. Patient characteristics of TCGA BRCA population, related to Figure 1 A total of 773 ductal and lobular breast carcinoma primary tumor samples were downloaded from the TCGA data portal and cleaned via the TAMPOR method. Listed previously is a summary of the clinical data for the TCGA BRCA data set. These clinical correlates are common factors influencing BrCa diagnoses and were correlated to the overriding patterns (MEs) of coexpressed transcripts during weighted gene coexpression network analysis.

Table S2. Breast cancer staging breakdown of cases in TCGA RNA-seq, related to Figure 1 Summary of pathologic and TNM staging breakout for 773 breast primary tumors in the TCGA BRCA data set excluding 4 outlier cases.

Table S3. RNA-seq case traits used for coexpression analysis (N = 773), related to Figure 1 Traits for individual cases in the TCGA BRCA data set were downloaded from the TCGA portal. TCGA cases listed as TNBC were confirmed via the Lehmann BD et al Web-based subtyping tool, TNBCtype. Results are included.

Table S4. Module size/rank and ME values (log2-scale) from TCGA RNA-Seq (N=773), related to Figures 2 and 4 Module size rank, module color, and number of genes present in each coexpression module after WGCNA network construction are provided along with the 773 sample-specific weighted expression patterns for each module.

Table S5. RNA-seq network module eigengene members and sorted kME values, related to Figure 2 and 4 Genes (n=31,338) are listed, followed by their membership scores for each module, and the module to which they were assigned by WGCNA clustering.

Table S6. Full TNBC versus receptor-positive subtypes DEG list, related to Figure 5 Genes are listed followed by T-test p-values, FDR (BH), log_2_(FPKM ratio), i.e., differences, and module colors. Genes implicated via GIANT for genetic interactions to M2 hubs and M12 hubs in mammary tissue are noted.

Table S7. Top 20 differentially expressed genes in TNBC versus Luminal A BrCa for M2-like and M12-like modules, related to Figure 5 The top 20 DEGs ranked by Benjamini-Hochberg FDR-corrected T-test p values in Table S6 (TNBC versus Luminal A comparison) for core M2- and M12-like modules are listed.

Table S8. Top 20 differentially expressed genes in TNBC versus HER2-enriched BrCa for M2-like and M12-like modules, related to Figure 5 The top 20 DEGs ranked by Benjamini-Hochberg FDR-corrected T-test p values in Table S6 (TNBC versus HER2-enriched comparison) for core M2- and M12-like modules are listed.

Table S9. GIANT analysis targets of hub genes for M12- and M2-like modules, related to Figure 7 Table generation relied on a broader list of hubs than the top 10 used for the GIANT graphs. Top-ranked hub genes (kME = 0.6 or higher) for the M12- and M2-like modules were entered into the GIANT server via the Flatiron Institute adapted interface to the GIANT database. Targets and weights for all interacting pairs of M12-like and M2-like hub genes are reported.

Table S10. Microarray case traits used for ROC testing (N = 1,234), related to Figure 8 Traits for individual arrays in GEO datasets curated originally by Gyöffry et al (N=1,079) (Györffy and Schäfer, 2009) and Karn et al (N=155) (Karn et al., 2011) were assembled from supplementary data in these references and GEO sample annotations. GEO cases listed as TNBC were confirmed via the Lehmann BD et al Web-based subtyping tool, TNBC type. Results are included.

Table S11. Round 1 ROC survival predictor gene expression ratio using Affy Arrays (N=1,234), related to Figure 8 and Table 3 381 combinations of sample-wise normalized merged array gene expression values were tested for ROC AUC at 5 timepoints in all BrCa subtypes and also in repeated analysis restricted to TNBC. Table as provided is sorted by best maximum AUC for the full data set (all BrCa subtypes).

Table S12. Round 2 ROC survival predictor gene expression ratio using Affy arrays (N=1,234), related to Figure 8 and Table 3 111,385 combinations of sample-wise normalized merged array gene expression values were tested for ROC AUC at 5 timepoints in all BrCa subtypes and also in repeated analysis restricted to TNBC. Table as provided is sorted by best maximum AUC for TNBC (N=180).

Data S1. Global plots of BrCa coexpression network identify modules correlated to TNBC subtype-relevant traits, Related to Figures 2, 3 and 4 The compilation of figures presented in Supplemental Dataset 1 is the full print out of results from WGCNA analysis. The dendrograms on pages 1 and 2 depict module eigengene relatedness between the 22 constructed modules. The heatmaps on pages 3–6 depict bicor module eigengene-trait correlations. The scatterplots on pages 7-28 depict Pearson correlation between module eigengenes and each clinical trait. Box plots measuring ANOVA eigenegene expression differences between the 4 BrCa subtypes, for each of the 22 modules, is also presented on pages 7–28. See also Figures 2 and 4. See also Tables S4 and S5.

Data S2. Global plots of BrCa coexpression network identify modules correlated to TNBC subtype-relevant traits with regression of age and race, Figure 2, 3, and 4 The compilation of figures presented in Supplemental Dataset 2 is also the full print out of results from WGCNA analysis. Age and race were regressed with bootstrap nonparametric regression, and identical module membership to that found in the unregressed data was used for calculation of eigengenes. See also Figures 2 and 4. See also Tables S4 and S5.
