# Supplementary material for: A network approach reveals driver genes associated with survival of patients with triple-negative breast cancer
Source: iScience. 2021 Apr 19;24(5):102451. doi: 10.1016/j.isci.2021.102451 (PMC8111681; doi:10.1016/j.isci.2021.102451)
Supplement: Document S1. Transparent methods and Figures S1–S4 [file mmc1.pdf]

iScience, Volume *24*

Supplemental information

A network approach reveals driver genes associated with survival of patients with triple-negative breast cancer

Courtney D. Dill, Eric B. Dammer, Ti'ara L. Griffen, Nicholas T. Seyfried, and James W. Lillard Jr.

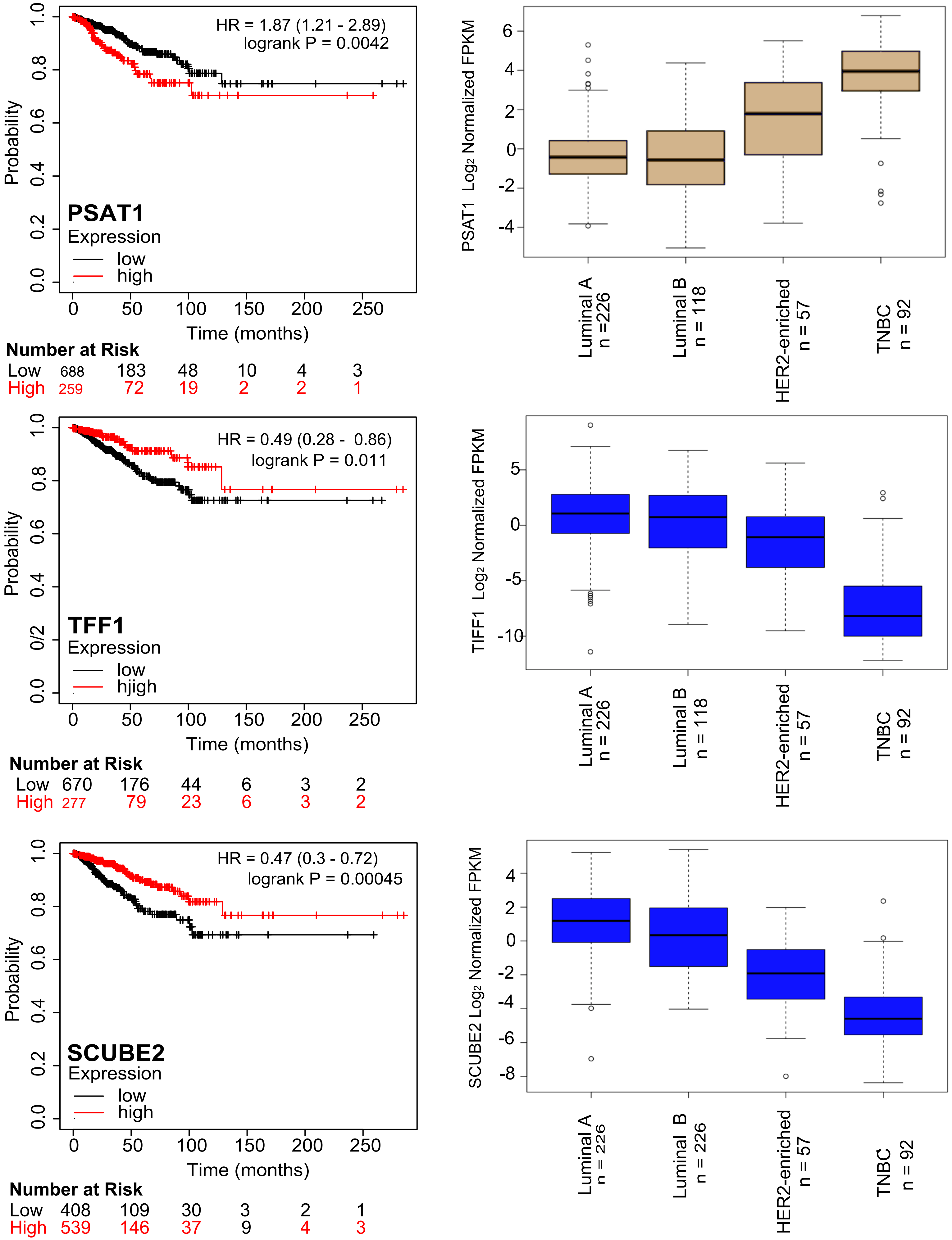

HR = 1.87 (1.21 - 2.89)
logrank P = 0.0042
Probability
PSAT1
Expression
low
high
Time (months)
Number at Risk
Low 688 183 48 10 4 3
High 259 72 19 2 2 1
PSAT1 Log2 Normalized FPKM
Luminal A n =226
Luminal B n = 118
HER2-enriched n = 57
TNBC n = 92
HR = 0.49 (0.28 - 0.86)
logrank P = 0.011
Probability
TFF1
Expression
low
hjigh
Time (months)
Number at Risk
Low 670 176 44 6 3 2
High 277 79 23 6 3 2
TIFF1 Log2 Normalized FPKM
Luminal A n = 226
Luminal B n = 118
HER2-enriched n = 57
TNBC n = 92
HR = 0.47 (0.3 - 0.72)
logrank P = 0.00045
Probability
SCUBE2
Expression
low
high
Time (months)
Number at Risk
Low 408 109 30 3 2 1
High 539 146 37 9 4 3
SCUBE2 Log2 Normalized FPKM
Luminal A n = 226
Luminal B n = 226
HER2-enriched n = 57
TNBC n = 92

Supplementary Figure 1, Related to Figure 6 and Table 2. Survival and gene expression analysis reveal association between PSAT1, TFF1, SCUBE2, and RFS in BrCa. Hazard ratios confirm low expression of PSAT1 resulted in significant high likelihood of relapse, while high expression of TFF1 and SCUBE2 resulted in significant less likelihood of relapse (left). The red line in each Kaplan-Meier plot represents survival of cases in the higher expression tier and the black line represents survival in cases with lower expression. A log-rank (Mantel-Cox) test determined *p*-values and hazard ratio (HR) scores. Survival results were validated by measuring mRNA gene expression reflected, as $\log_2$ FPKM, via box and whisker plots (right). Gene expression of PSAT1 mRNA was highest among the TNBC subtype biopsy group compared to non-TNBC, while TFF1 and SCUBE2 were lowest in TNBC. A two group, Wilcoxon rank-sum, test determined *p*-values.

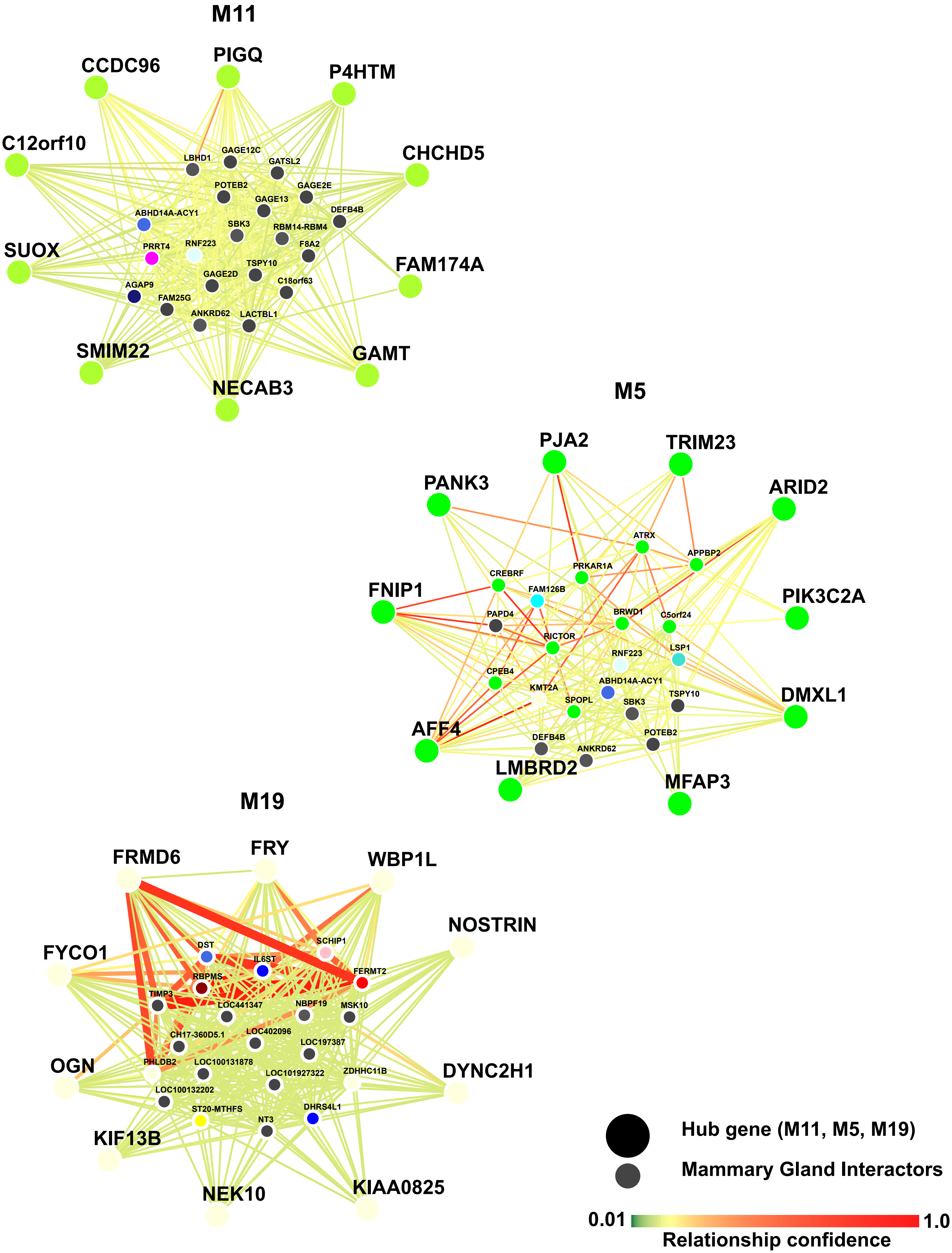
M11
PIGQ
CCDC96
P4HTM
C12orf10
CHCHD5
SUOX
FAM174A
SMIM22
GAMT
NECAB3
LBHD1
GAGE12C
GATSL2
POTEB2
GAGE2E
GAGE13
DEFB4B
ABHD14A-ACY1
SBK3
RBM14-RBM4
PRRT4
RNF223
F8A2
TSPY10
GAGE2D
C18orf63
AGAP9
FAM25G
ANKRD62
LACTBL1
M5
PJA2
TRIM23
PANK3
ARID2
ATRX
APPBP2
CREBRF
PRKAR1A
FAM126B
FNIP1
PIK3C2A
PAPD4
BRWD1
C5orf24
RICTOR
RNF223
LSP1
CPEB4
ABHD14A-ACY1
KMT2A
TSPY10
SPOPL
SBK3
DMXL1
AFF4
DEFB4B
POTEB2
ANKRD62
LMBRD2
MFAP3
M19
FRMD6
FRY
WBP1L
NOSTRIN
FYCO1
DST
SCHIP1
IL6ST
RBPMS
FERMT2
TIMP3
LOC441347
NBPF19
MSK10
LOC402096
CH17-360D5.1
LOC197387
OGN
PHLDB2
LOC100131878
ZDHHC11B
DYNC2H1
LOC101927322
LOC100132202
ST20-MTHFS
DHRS4L1
NT3
KIF13B
NEK10
KIAA0825
Hub gene (M11, M5, M19)
Mammary Gland Interactors
0.01
1.0
Relationship confidence

Supplementary Figure 2, Related to Figure 7. Genome-scale integrated analysis of gene networks in tissues (GIANT) identifies lower connectivity breast tissue-specific interactions between survival-linked genes and module hub-implicated genes as well as hubs in M2-like modules (M11, M5, M19). The heatmap scale represents the confidence of the predicted interactions based on mammary gland specific interactions. High confidence interactions are represented by red edges (see scale). Input for GIANT was restricted to the top ten hub genes (kME = 0.6 or higher) for each module. Small nodes in the center of each network are implicated by the GIANT database network as the best connected to the hubs specified in mammary tissue, colored by their module membership.

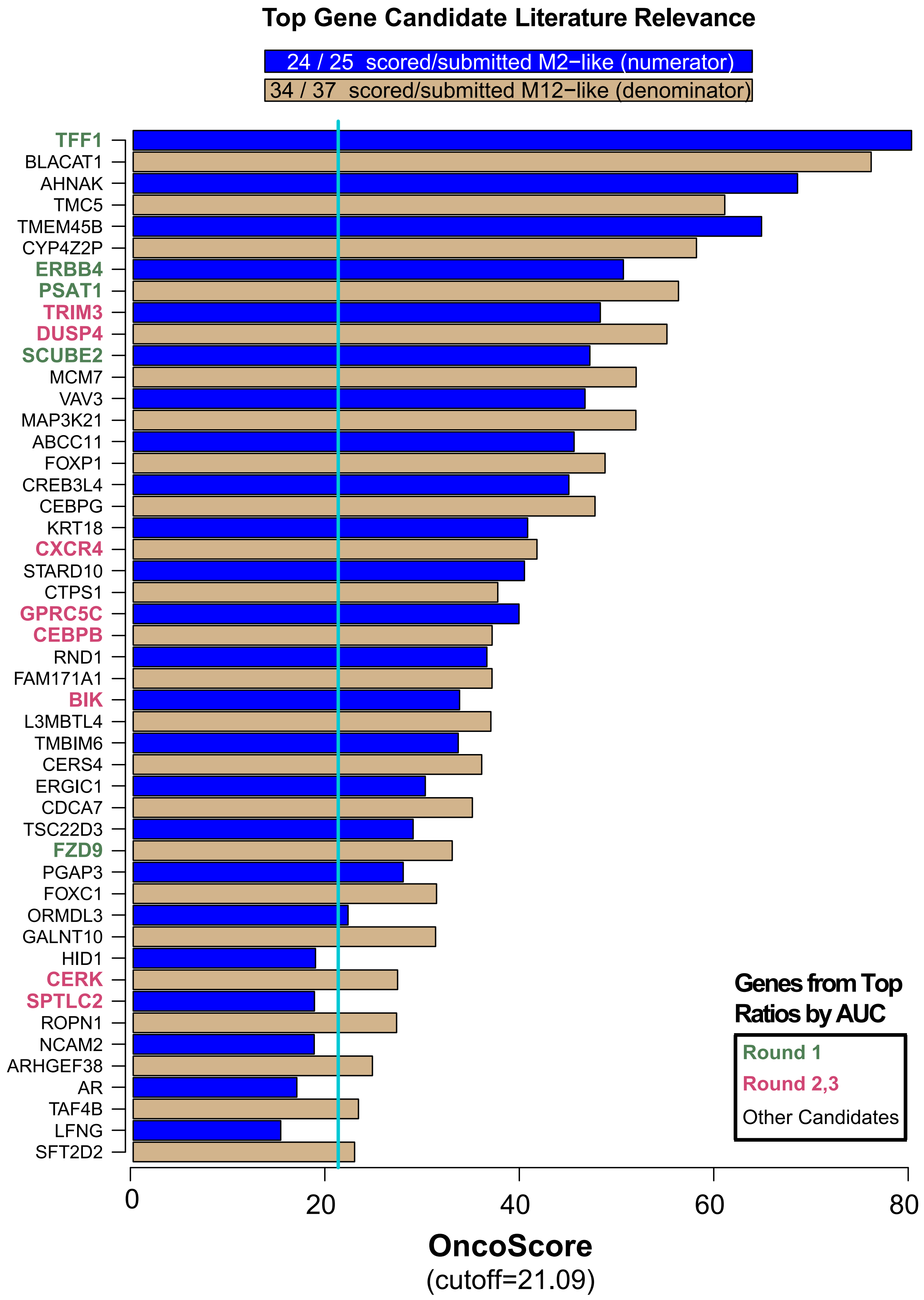

Top Gene Candidate Literature Relevance
24 / 25 scored/submitted M2−like (numerator)
34 / 37 scored/submitted M12−like (denominator)
TFF1
BLACAT1
AHNAK
TMC5
TMEM45B
CYP4Z2P
ERBB4
PSAT1
TRIM3
DUSP4
SCUBE2
MCM7
VAV3
MAP3K21
ABCC11
FOXP1
CREB3L4
CEBPG
KRT18
CXCR4
STARD10
CTPS1
GPRC5C
CEBPB
RND1
FAM171A1
BIK
L3MBTL4
TMBIM6
CERS4
ERGIC1
CDCA7
TSC22D3
FZD9
PGAP3
FOXC1
ORMDL3
GALNT10
HID1
CERK
SPTLC2
ROPN1
NCAM2
ARHGEF38
AR
TAF4B
LFNG
SFT2D2
Genes from Top Ratios by AUC
Round 1
Round 2,3
Other Candidates
0
20
40
60
80
OncoScore
(cutoff=21.09)

Supplementary Figure 3, Related to Figure 8. Scored representation of gene products in the cancer literature. OncoScore (Piazza et al., 2017) was used to determine the relevance of gene products to cancer based on existing literature on Pubmed. The top scoring 24 M2-like (numerator) and 24 M12-like (denominator) gene symbols are shown.

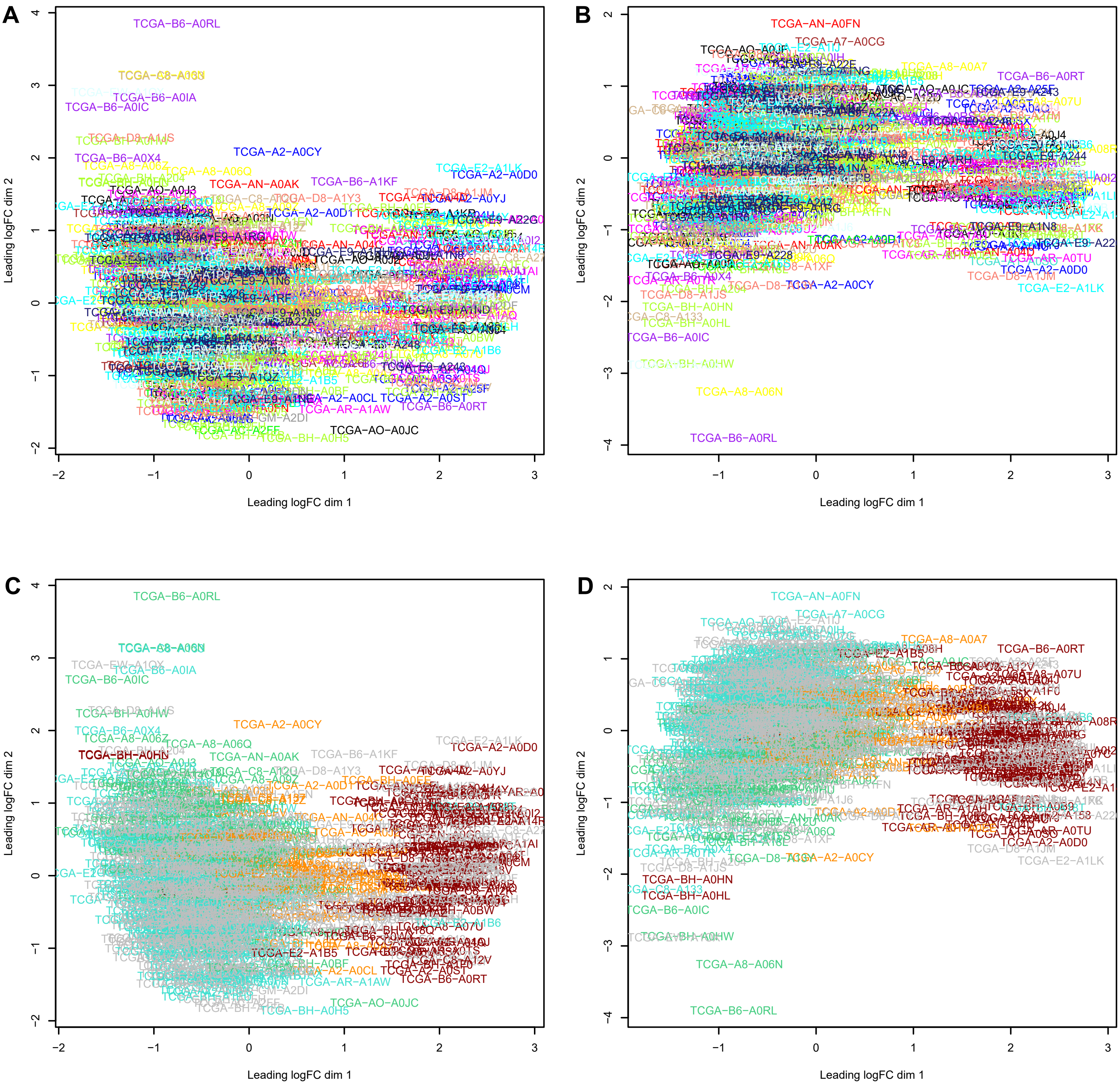
A
B
C
D
Leading logFC dim 1
Leading logFC dim 2
TCGA−B6−A0RL
TCGA−AN−A0FN
TCGA−A7−A0CG
TCGA−A8−A06N
TCGA−B6−A0IC
TCGA−B6−A0IA
TCGA−A2−A0CY
TCGA−B6−A0RT
TCGA−A8−A0A7
TCGA−E2−A1LK
TCGA−A2−A0D0
TCGA−AO−A0JC
TCGA−BH−A0HN
TCGA−BH−A0HL
TCGA−BH−A0HW
TCGA−B6−A0X4
TCGA−AN−A0AK
TCGA−B6−A1KF
TCGA−AR−A1AW
TCGA−A2−A0ST
TCGA−A2−A0CL
TCGA−E2−A1B5
TCGA−A2−A0YJ
TCGA−A8−A06Q
TCGA−D8−A1Y3

Supplementary Figure 4, Related to Figure 1. PCA plots of TCGA BRCA dataset without and with zero-FPKM gene filtering (removal of genes with ≥50% zero values), outlier removal (using WGCNA sample connectivity>3SD), and central tendency two-way table median polish transformation (batch site effect removal). Principle component analysis was performed on the TCGA BrCa log2(FPKM) data before (A) and after (B) these steps. Variance of the top 500 contributing genes within the dataset was captured by the limma R package plotMDS function in 2 dimensions, PC1 and PC2. Changes in the range of the y axis post filtering, outlier removal, and transformation were minimal, and batches represented by different colors in panels A and B are not tightly clustered, indicating variation due to batch effect was also minimal. The same plots with samples colored by BrCa subtype (Luminal A, turquoise; Luminal B, seagreen; HER2-enriched, orange; TNBC, darkred) are shown in panels C and D.

Transparent Methods

Data curation and normalization. RNA-Seq data for co-expression analysis in this study was obtained from The Cancer Genome Atlas (TCGA) BRCA project. Breast primary tumor samples were collected from patients with lobular and ductal breast carcinomas with informed consent and IRB approval (Koboldt et al., 2012). FPKM normalized transcript abundance for 777 primary tumors were downloaded from the TCGA portal on or before August 27, 2019. An overview of clinical trait breakdowns collected for 777 case samples is provided (**Supplementary Tables S1- S3**). Level 3 RNA-Seq data was used for this study, which is de-identified and publicly available through TCGA online. Corrected, augmented trait data for all TCGA cases (Liu et al., 2018) was obtained and used for more accurate RFS information.

For array data used in ROC analysis, we curated 1,234 case-sample array-based measurements of transcriptome expression of BrCa of all subtypes selected from meta-analysis studies of BrCa (Györffy and Schäfer, 2009) and TNBC (Karn et al., 2011) as a coherent dataset, made possible by all these arrays sharing a common probe set and design. All data are available as GEO datasets (listed with traits in **Supplementary Table S10**), and as published supplementary data already normalized. MAS5.0-normalized expression data from both meta-analysis studies (Györffy and Schäfer, 2009, Karn et al., 2011) was aligned to use a common three-step sample-wise normalization as described in Karn, *et al* (Karn et al., 2014) prior to merging of the data. 155 TNBC cases from this meta-analysis was used, and a total of 180 TNBC cases were represented. All cases were annotated with relapse occurrence and RFS as necessary traits. Tumor samples identified as TNBC for the TCGA and GEO datasets were confirmed as subtypes of TNBC using the web based TNBCtype tool described by Lehmann BD et al (Chen et al., 2012, Lehmann et al., 2011). Subclassification of TNBC samples can be found in (Supplementary Table S3 and S10).

Data normalization, site effect handling, and removal of outliers. TCGA BrCa RNA-seq FPKM data from multiple collection sites (N=777 baseline case samples with 64,483 gene-wise short read-based quantification) was curated, addressing potential technical, site-specific sources of variance and of noise before gene clustering for co-expression. Only transcripts that had less than 50 percent of values censored were retained. 31,338 genes remained. Site or batch effect handling, normalization, and quality assessment relied on a Tunable Approach for Median Polish of Ratio (TAMPOR). This R-based function implements a two-way table median polish algorithm (Tukey, 1977) and acts on a log-transformed ratio of specific sample signals to the central tendency of a set of case samples common to all batches, which may be biological or technical replicates, or mixed pools of all samples (Johnson et al., 2020).

To capture biological variance of TNBC vs. non-TNBC groups, and to preserve it through normalization, all samples (N =777) for the TCGA-BrCa cohort were processed jointly in the sample-gene transcript matrix. TAMPOR implements user choice (i.e., tunability) for which samples are considered in Eq. 1 to obtain ratio denominators representing central tendency within and across batches for gene- (row)-wise median normalization in both terms of the equation.

[Eq. 1] $$\frac{abundance}{median\ (ALL\ SAMPLEs)_{\ site}} * \frac{grand\ median}{\boldsymbol{median}\left(\{\ \frac{\boldsymbol{abundance}}{\boldsymbol{median\ (ALL\ SAMPLEs)}_{\ site}}\ \mid \boldsymbol{all\ samples\ from\ site}\ \}\right)}$$

Equation 1 applies to each measurement of a given gene transcript across all samples, where the first term represents site-wise median-centered abundance of a specific sample, and the second term is a site-specific normalization factor comprised of the grand median of all site-specific multi-sample ($n \geq 5$) medians, divided by the site-specific multi-sample median ratio. Ratios are log_2-transformed, each log_2-transformed ratio is adjusted sample- (column-) wise to a median value of 0, via subtraction of the sample's median log_2-transformed ratio for all transcripts. Ratios are then anti-logged and multiplied by the row-wise abundance mean, extracted at the beginning of the iteration. The process is repeated for a default of 250 iterations

or until convergence achieving an absolute value of the Frobenius norm difference from the previous iteration $| (||A||_{F(n-1)} - ||A||_{F(n)}) | < 1.0x10^{-8}$.

TAMPOR-normalized relative gene abundances were log_2-transformed, then checked for network connectivity outliers 3 or more standard deviations from the average z-transformed sample connectivity calculated using the WGCNA R package fundamentalNetworkConcepts function (Oldham et al., 2008). This process is repeated until no outliers are detected. We then used principle component analysis implemented in the limma package plotMDS function to visualize TAMPOR-mediated removal of batch effects after removal of outliers vs. the original data before median polish, with zeroes also censored by log_2-transformation. The samples showed no apparent systematic site effect but did separate by BrCa subtype, particularly TNBC (**Supplementary Figure 4**).

WGCNA for co-expressed gene clustering. The weighted gene co-expression network analysis (WGCNA) R software package was used to assess gene co-expression profiles across breast tumor samples, with the TAMPOR-normalized FPKM abundance matrix as input, and module eigengenes as the key output, which are correlated gene co-expression patterns (Oldham et al., 2008, Langfelder and Horvath, 2012). Eigengenes representing each module are assessed for correlation to sample-specific disease-related and other traits of interest, thereby enabling downstream analyses to focus attention on disease-relevant modules. WGCNA identifies gene clusters by calculating a gene dissimilarity matrix (1-topology overlap matrix) across samples and clustering genes that have similar expression patterns within the patient cohort into modules. As described previously, (Ohandjo et al., 2019) the WGCNA package blockwiseModules function was used for network construction, with module detection features including calls to the WGCNA dynamic tree-cutting algorithm, cutreeHybrid (Oldham et al., 2008, Langfelder and Horvath, 2012). Parameters for the function were as follows: power=6, deepsplit=4, minModuleSize=100, mergeCutHeight=0.15, TOMDenom="mean",

corType="bicor", networkType="signed", pamStage=TRUE, pamRespectsDendro=TRUE, maxBlockSize larger than the number of genes being clustered (40000), and reassignThresh=0.05. We used biweight midcorrelation (bicor), as opposed to Pearson correlation, to provide robust correlations with less weight given to outlier measures (Oldham et al., 2008, Langfelder and Horvath, 2012). RNA-seq data often has high dynamic range and therefore often exhibits high variance across samples, making bicor rho and associated Student *p* values ideal for summarizing correlation robustly, a pivotal feature of the analysis.

Similarity between WGCNA module eigengenes (the first principle component of co-expression in a module) was determined by assessing module eigengene relatedness. Eigengenes are the first principal component of each module, overweighting the most highly correlated gene profiles across case samples that contribute to a co-expression network. The WGCNA blockwiseModules function provided an output of eigengene values for each module, further correlated in pairs. The output from this analysis depicted a relatedness dendrogram indicating the relatedness of all modules. This attribute of WGCNA, in addition to module trait correlation, drew attention to the identification of modules of interest.

Differential expression. An unpaired, two-tailed, equal variance hypothesis t-test was conducted to compare differential expression of TNBC subtyped tumor biopsies (N=92) to the non-TNBC group comprised of Luminal A (N=226), Luminal B (N=118), and HER2-enriched (N=57) subtyped tumor biopsies. Tumor samples missing subtype information were excluded from differential expression analysis. The t test is robust to noisy or skewed RNA-seq data and well-accommodates the unequal sample sizes of each group. FDR adjustment was performed using the Benjamini-Hochberg method. Three comparisons were considered to determine differential expression in TNBC: TNBC vs. Luminal A, TNBC vs. Luminal B, and TNBC vs. HER2-enriched subtyped sample abundances. For inclusion in **Supplementary Table S6**, a

threshold was set at FDR<0.0001 for each of the above comparisons, and DEGs were only reported if significant and consistent direction of change occurred in all three comparisons.

Gene ontology (GO) enrichment analysis. GO-Elite v.1.2.5, was used to perform GO enrichment analysis on the M12 and M2 modules to identify overall module enrichment of biological functions (Zambon et al., 2012, Young et al., 2010). Version 62 of the Ensembl database (comprised of pre-defined gene lists organized by biological process, molecular function, and cellular component) was selected for standard GO enrichment analysis. Fisher's exact test p, adjusted for false discovery, was used to determine overrepresentation or significant overlap between members of WGCNA modules of interest and pre-defined gene lists (Young et al., 2010). The reference background gene list was the subset of 31,338 genes with symbols in the final cleaned abundance matrix (23,241 symbols).

Survival and gene expression analysis. Survival analysis was performed across 773 individuals to determine M12- and M-2 like eigengene value association to PFI using R packages, survival and survival miner. Generation of survival plots with optimal high/low cutoff for each gene were performed using the cutpoint R package with default parameters and the cutpointr function; cutpointr(data=survTraits, thisME, PFI.1, method = maximize_metric, metric = sum_sens_spec). Additionally, association to RFS for select genes from selected modules, M12 and M2, was determined using KMplotter (Gyorffy et al., 2010). The tool assesses the relationship of 54,000 gene transcript levels individually to RFS in various cancers. Analysis using gene expression paired with patient survival was performed using the Pan Cancer Atlas BrCa dataset (n = 1,090). These data were curated by authors of KMplotter from the Genome Expression Omnibus (GEO), the European Genome-Phenome Archive (EGA), and TCGA. Generation of survival plots was performed with KMplot.com default parameters except for enabling of auto-selection of an optimal high/low cutoff for each gene. Patient samples for the

eigengene value and single gene curves were split into two groups (high and low expressers) according to median and mean expression to analyze the prognostic value of a gene. A log-rank (Mantel-Cox) test was used to determine p-values and hazard ratio with 95% confidence intervals for both sets of KM analyses. Box plots of TCGA RNA-seq relative abundance in **Supplementary Figure 1** were graphed using the base R boxplot function applied to TAMPOR-normalized log_2 relative abundance. Significance of TNBC vs. non-TNBC groupwise differences was tested with a Mann-Whitney U test.

Genome-scale integrated analysis of gene networks in tissues (GIANT). GIANT version 2.0 (Wong et al., 2018) was used to examine the breast tissue-specific interaction network of the top ten hubs of the M2- and M12-like modules and selected survival-associated genes. Module memberships of genes predicted to interact with the top hub genes and rankings of the hubs were referenced by kME for each gene transcript's assigned module, as listed in **Supplementary Table S5**. Targets and weights for the top ranked hub genes (kME = 0.6 or higher) were obtained via the Flatiron Institute adapted interface to the GIANT database.

ROC testing of combinatorial transcript ratios as predictors of survival. Sample-wise normalized array probe abundances were collapsed to gene-level measurements keeping probes with maximal variance. Nominated genes represented on the arrays were then combined in all 1:1, 2:2, or 3:3 combinations and unweighted ratios were calculated for each sample. RFS was used as a proxy for survival in general, informing generalized linear model fits of each set of ratio calculations (the predictor) to RFS outcome at five different survival times. Survival is irrespective of treatment received by patients as these records are not available. Follow-up duration for this cohort was 10 years for all BrCa subtypes and 7.5 years specifically for TNBC patients. Binomial variance family with default linkage were specified for the R glm function. Time-dependent binary RFS outcome and the ordered glm-fit predictor were then

tested and plot by the roc function of the pROC R package. AUC was used to rank predictor ratios. The verification R package provided functions for calculation of AUC Mann-Whitney U-based p value and 95% CI based on the calculated ROC curves. A one-tailed test for significance of improvement in ROC curve pairs was performed using a bootstrap method (n=2,000 permutations) as implemented in the roc.test function.

Supplemental References

Chen, X., Li, J., Gray, W. H., Lehmann, B. D., Bauer, J. A., Shyr, Y. & Pietenpol, J. A. 2012. TNBCtype: A Subtyping Tool for Triple-Negative Breast Cancer. *Cancer Inform,* 11**,** 147-56.

Gyorffy, B., Lanczky, A., Eklund, A., Denkert, C., Budczies, J., Li, Q. & Szallasi, Z. 2010. An online survival analysis tool to rapidly assess the effect of 22,277 genes on breast cancer prognosis using microarray data of 1,809 patients. *Breast Cancer Research and Treatment,* 123**,** 725-731.

Györffy, B. & Schäfer, R. 2009. Meta-analysis of gene expression profiles related to relapse-free survival in 1,079 breast cancer patients. *Breast Cancer Res Treat,* 118**,** 433-41.

Johnson, E. C. B., Dammer, E. B., Duong, D. M., Ping, L., Zhou, M., Yin, L., Higginbotham, L. A., Guajardo, A., White, B., Troncoso, J. C., et al. 2020. Large-scale proteomic analysis of Alzheimer's disease brain and cerebrospinal fluid reveals early changes in energy metabolism associated with microglia and astrocyte activation. *Nature Medicine,* 26**,** 769-780.

Karn, T., Pusztai, L., Holtrich, U., Iwamoto, T., Shiang, C. Y., Schmidt, M., Müller, V., Solbach, C., Gaetje, R., Hanker, L., et al. 2011. Homogeneous datasets of triple negative breast cancers enable the identification of novel prognostic and predictive signatures. *PloS one,* 6**,** e28403-e28403.

Karn, T., Rody, A., Müller, V., Schmidt, M., Becker, S., Holtrich, U. & Pusztai, L. 2014. Control of dataset bias in combined Affymetrix cohorts of triple negative breast cancer. *Genomics data,* 2**,** 354-356.

Koboldt, D. C., Fulton, R. S., Mclellan, M. D., Schmidt, H., Kalicki-Veizer, J., Mcmichael, J. F., Fulton, L. L., Dooling, D. J., Ding, L., Mardis, E. R., et al. 2012. Comprehensive molecular portraits of human breast tumours. *Nature,* 490**,** 61-70.

Langfelder, P. & Horvath, S. 2012. Fast R Functions for Robust Correlations and Hierarchical Clustering. *J Stat Softw,* 46.

Lehmann, B. D., Bauer, J. A., Chen, X., Sanders, M. E., Chakravarthy, A. B., Shyr, Y. & Pietenpol, J. A. 2011. Identification of human triple-negative breast cancer subtypes and preclinical models for selection of targeted therapies. *The Journal of clinical investigation,* 121**,** 2750-2767.

Liu, J., Lichtenberg, T., Hoadley, K. A., Poisson, L. M., Lazar, A. J., Cherniack, A. D., Kovatich, A. J., Benz, C. C., Levine, D. A., Lee, A. V., et al. 2018. An Integrated TCGA Pan-Cancer Clinical Data Resource to Drive High-Quality Survival Outcome Analytics. *Cell,* 173**,** 400-416.e11.

Ohandjo, A. Q., Liu, Z., Dammer, E. B., Dill, C. D., Griffen, T. L., Carey, K. M., Hinton, D. E., Meller, R. & Lillard, J. W., Jr. 2019. Transcriptome Network Analysis Identifies CXCL13-CXCR5 Signaling Modules in the Prostate Tumor Immune Microenvironment. *Sci Rep,* 9**,** 14963.

Oldham, M. C., Konopka, G., Iwamoto, K., Langfelder, P., Kato, T., Horvath, S. & Geschwind, D. H. 2008. Functional organization of the transcriptome in human brain. *Nature Neuroscience,* 11**,** 1271-1282.

Piazza, R., Ramazzotti, D., Spinelli, R., Pirola, A., De Sano, L., Ferrari, P., Magistroni, V., Cordani, N., Sharma, N. & Gambacorti-Passerini, C. 2017. OncoScore: a novel, Internet-based tool to assess the oncogenic potential of genes. *Scientific Reports,* 7**,** 46290.

Tukey, J. W. 1977. *Exploratory data analysis*, Reading, Mass.

Wong, A. K., Krishnan, A. & Troyanskaya, O. G. 2018. GIANT 2.0: genome-scale integrated analysis of gene networks in tissues. *Nucleic Acids Research,* 46**,** W65-W70.

Young, M. D., Wakefield, M. J., Smyth, G. K. & Oshlack, A. 2010. Gene ontology analysis for RNA-seq: accounting for selection bias. *Genome biology,* 11**,** R14.

Zambon, A. C., Gaj, S., Ho, I., Hanspers, K., Vranizan, K., Evelo, C. T., Conklin, B. R., Pico, A. R. & Salomonis, N. 2012. GO-Elite: a flexible solution for pathway and ontology over-representation. *Bioinformatics,* 28**,** 2209-10.
