# Supplementary material for: A network approach reveals driver genes associated with survival of patients with triple-negative breast cancer
Source: iScience. 2021 Apr 19;24(5):102451. doi: 10.1016/j.isci.2021.102451 (PMC8111681; doi:10.1016/j.isci.2021.102451)
Supplement: Data S1. Global plots of BrCa coexpression network identify modules correlated to TNBC subtype-relevant traits, Related to Figures 2, 3 and 4 — The compilation of figures presented in Supplemental Dataset 1 is the full print out of results from WGCNA analysis. The dendrograms on pages 1 and 2 depict module eigengene relatedness between the 22 constructed modules. The heatmaps on pages 3–6 depict bicor module eigengene-trait correlations. The scatterplots on pages 7-28 depict Pearson correlation between module eigengenes and each clinical trait. Box plots measuring ANOVA eigenegene expression differences between the 4 BrCa subtypes, for each of the 22 modules, is also presented on pages 7–28. See also Figures 2 and 4. See also Tables S4 and S5. [file mmc14.zip › Supplemental Dataset1.pdf]

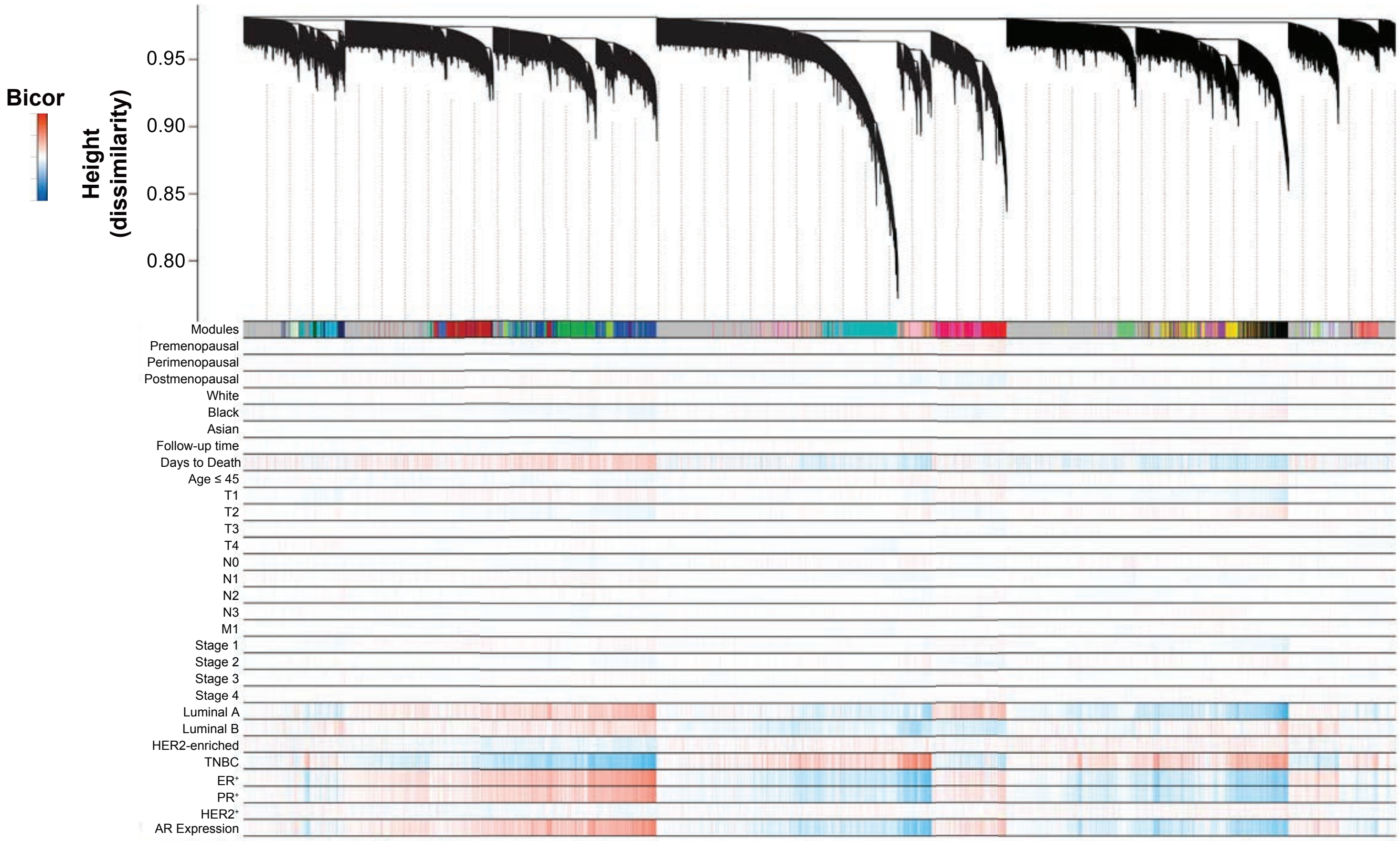

Bicor
Height (dissimilarity)
0.95
0.90
0.85
0.80
Modules
Premenopausal
Perimenopausal
Postmenopausal
White
Black
Asian
Follow-up time
Days to Death
Age ≤ 45
T1
T2
T3
T4
N0
N1
N2
N3
M1
Stage 1
Stage 2
Stage 3
Stage 4
Luminal A
Luminal B
HER2-enriched
TNBC
ER+
PR+
HER2+
AR Expression

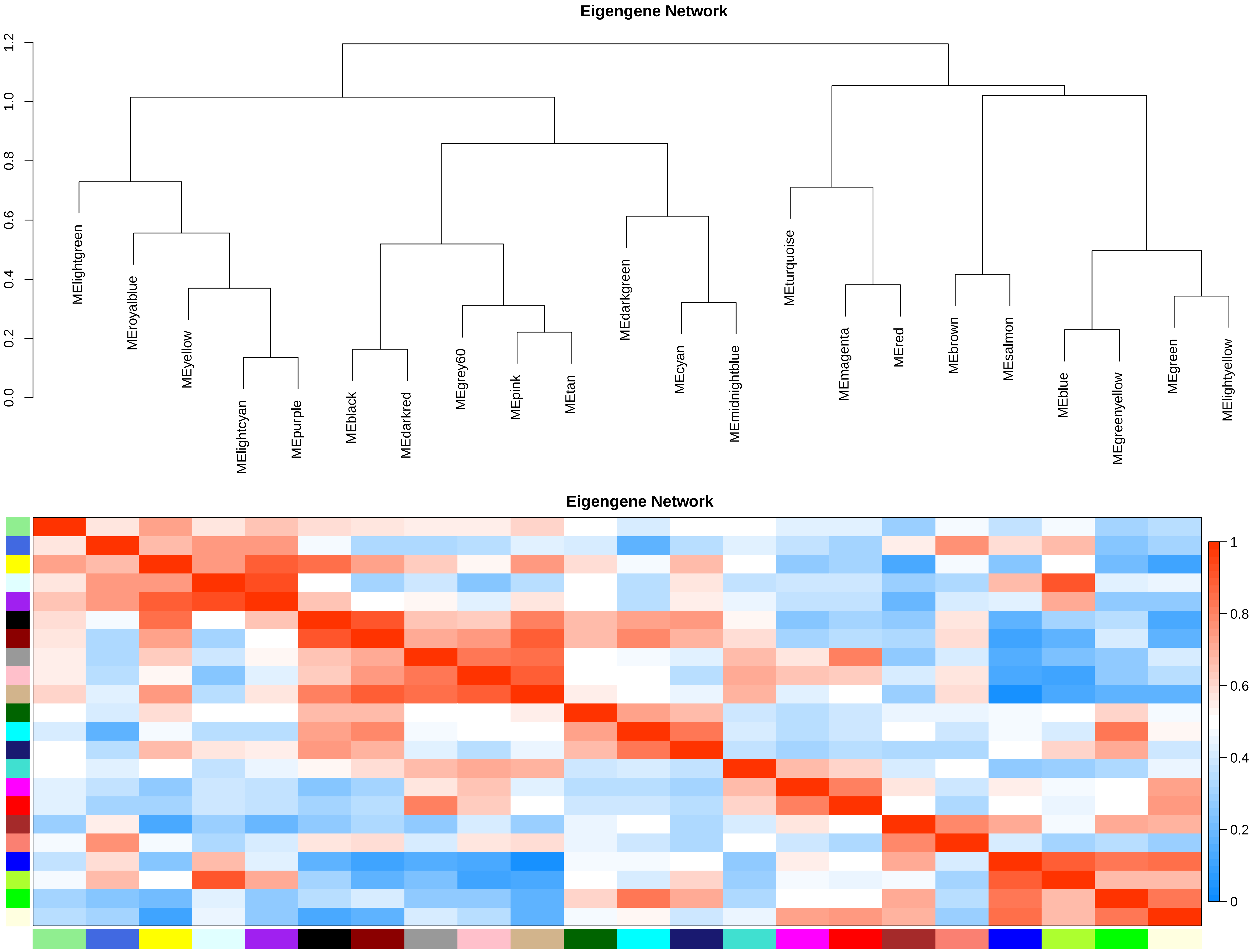
Eigengene Network
0.0
0.2
0.4
0.6
0.8
1.0
1.2
MElightgreen
MEroyalblue
MEyellow
MElightcyan
MEpurple
MEblack
MEdarkred
MEgrey60
MEpink
MEtan
MEdarkgreen
MEcyan
MEmidnightblue
MEturquoise
MEmagenta
MEred
MEbrown
MEsalmon
MEblue
MEgreenyellow
MEgreen
MElightyellow
Eigengene Network
1
0.8
0.6
0.4
0.2
0

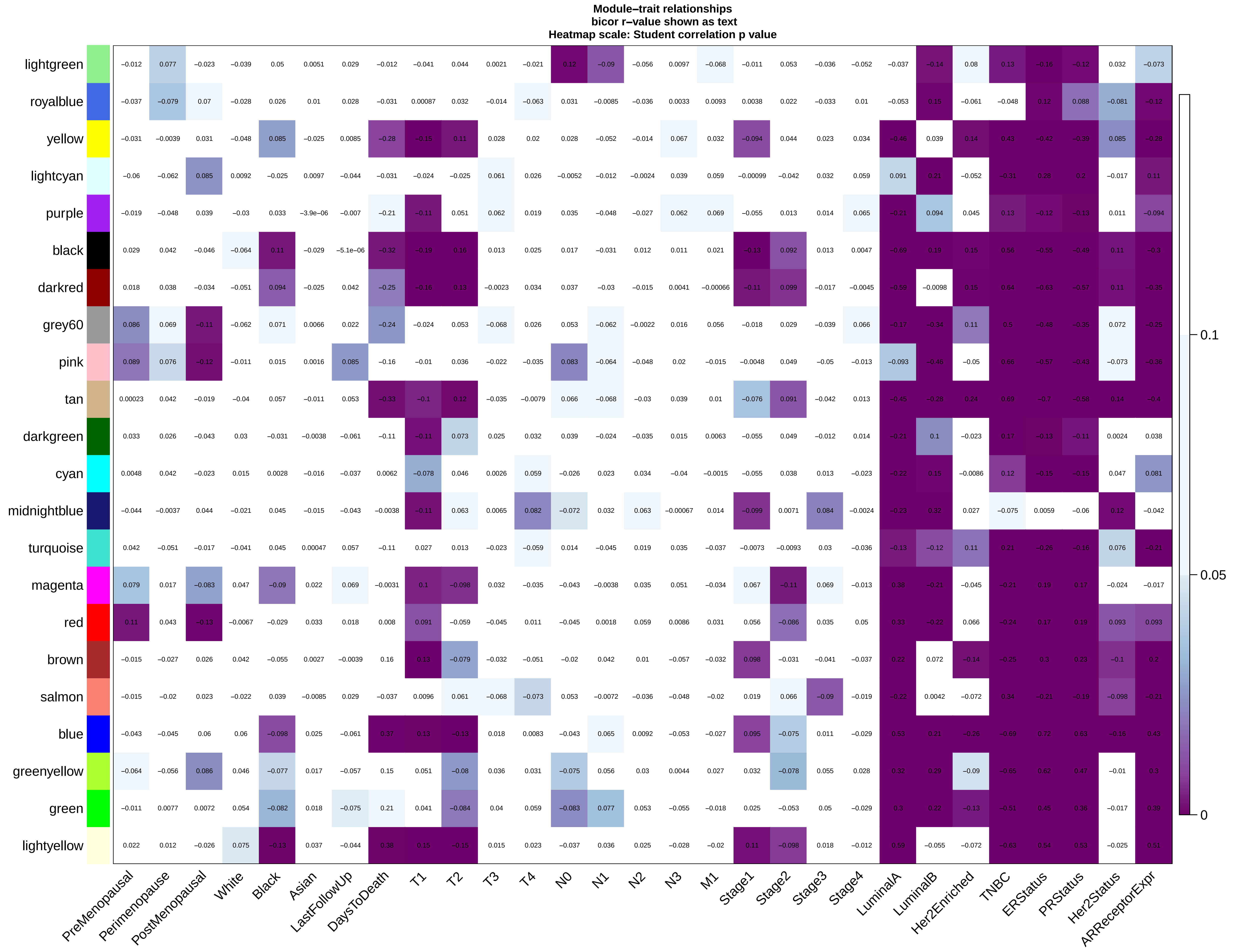
Module–trait relationships
bicor r–value shown as text
Heatmap scale: Student correlation p value
lightgreen
royalblue
yellow
lightcyan
purple
black
darkred
grey60
pink
tan
darkgreen
cyan
midnightblue
turquoise
magenta
red
brown
salmon
blue
greenyellow
green
lightyellow
PreMenopausal
Perimenopause
PostMenopausal
White
Black
Asian
LastFollowUp
DaysToDeath
T1
T2
T3
T4
N0
N1
N2
N3
M1
Stage1
Stage2
Stage3
Stage4
LuminalA
LuminalB
Her2Enriched
TNBC
ERStatus
PRStatus
Her2Status
ARReceptorExpr
0.1
0.05
0

Figure 4

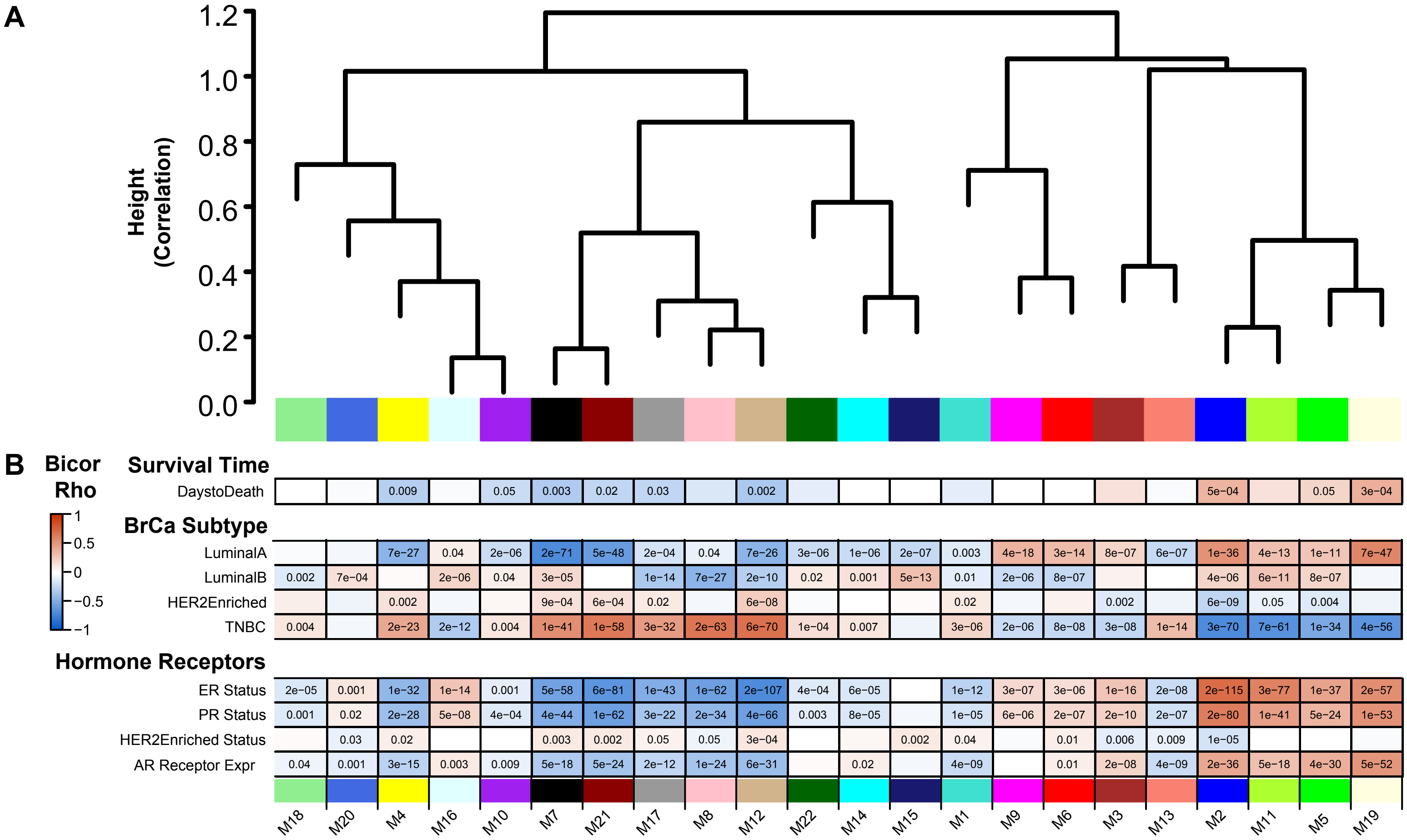
A
Height (Correlation)
1.2
1.0
0.8
0.6
0.4
0.2
0.0
B
Bicor Rho
1
0.5
0
−0.5
−1
Survival Time
DaystoDeath 0.009 0.05 0.003 0.02 0.03 0.002 5e−04 0.05 3e−04
BrCa Subtype
LuminalA 7e−27 0.04 2e−06 2e−71 5e−48 2e−04 0.04 7e−26 3e−06 1e−06 2e−07 0.003 4e−18 3e−14 8e−07 6e−07 1e−36 4e−13 1e−11 7e−47
LuminalB 0.002 7e−04 2e−06 0.04 3e−05 1e−14 7e−27 2e−10 0.02 0.001 5e−13 0.01 2e−06 8e−07 4e−06 6e−11 8e−07
HER2Enriched 0.002 9e−04 6e−04 0.02 6e−08 0.02 0.002 6e−09 0.05 0.004
TNBC 0.004 2e−23 2e−12 0.004 1e−41 1e−58 3e−32 2e−63 6e−70 1e−04 0.007 3e−06 2e−06 8e−08 3e−08 1e−14 3e−70 7e−61 1e−34 4e−56
Hormone Receptors
ER Status 2e−05 0.001 1e−32 1e−14 0.001 5e−58 6e−81 1e−43 1e−62 2e−107 4e−04 6e−05 1e−12 3e−07 3e−06 1e−16 2e−08 2e−115 3e−77 1e−37 2e−57
PR Status 0.001 0.02 2e−28 5e−08 4e−04 4e−44 1e−62 3e−22 2e−34 4e−66 0.003 8e−05 1e−05 6e−06 2e−07 2e−10 2e−07 2e−80 1e−41 5e−24 1e−53
HER2Enriched Status 0.03 0.02 0.003 0.002 0.05 0.05 3e−04 0.002 0.04 0.01 0.006 0.009 1e−05
AR Receptor Expr 0.04 0.001 3e−15 0.003 0.009 5e−18 5e−24 2e−12 1e−24 6e−31 0.02 4e−09 0.01 2e−08 4e−09 2e−36 5e−18 4e−30 5e−52
M18
M20
M4
M16
M10
M7
M21
M17
M8
M12
M22
M14
M15
M1
M9
M6
M3
M13
M2
M11
M5
M19

Plot of Eigengene–Trait Relationships – SAMPLES IN ORIGINAL, e.g. BATCH OR REGION ORDER

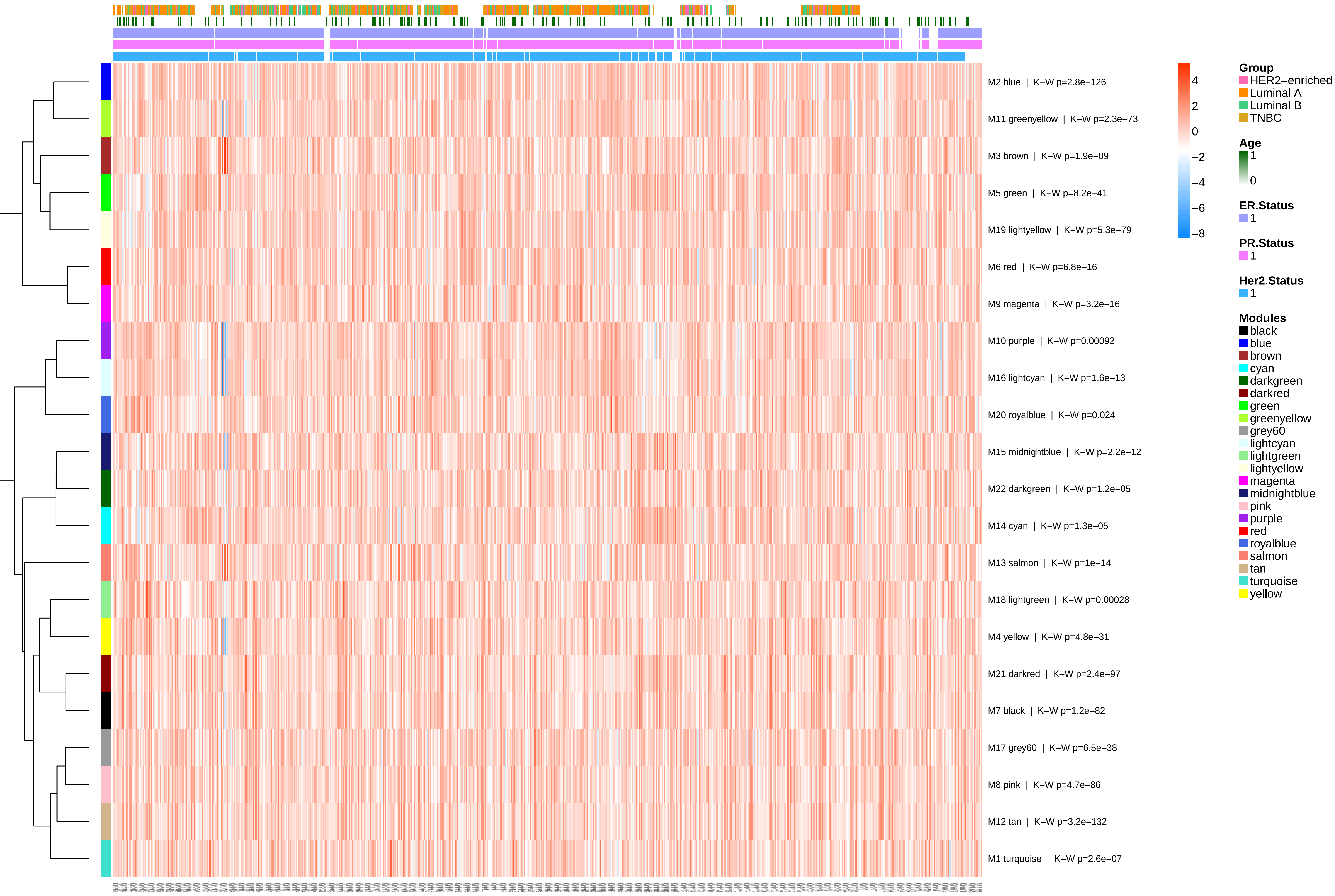

Plot of Eigengene–Trait Relationships – SAMPLES CLUSTERED

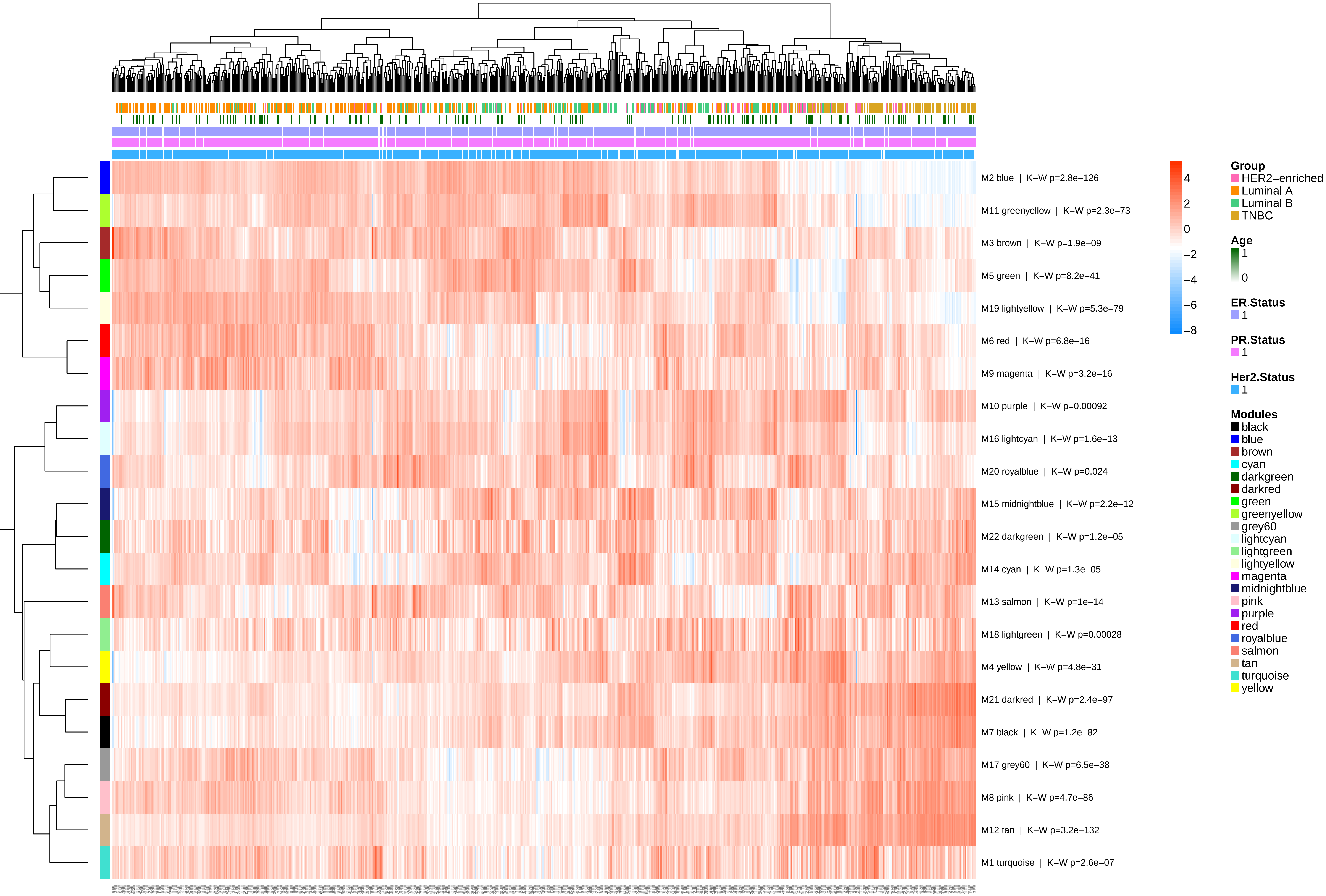

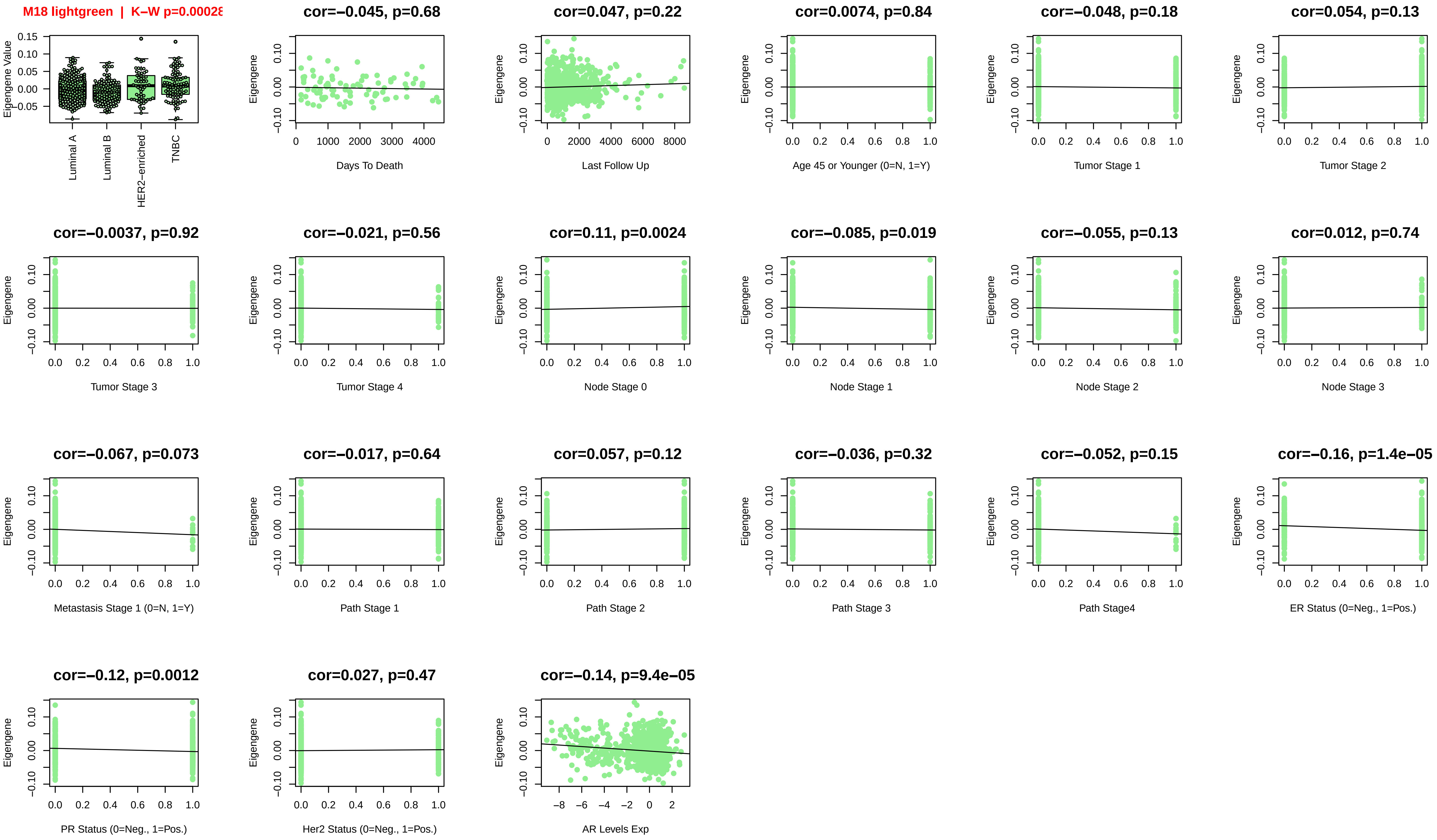
M18 lightgreen | K–W p=0.00028
Eigengene Value
Luminal A
Luminal B
HER2–enriched
TNBC
cor=–0.045, p=0.68
Days To Death
cor=0.047, p=0.22
Last Follow Up
cor=0.0074, p=0.84
Age 45 or Younger (0=N, 1=Y)
cor=–0.048, p=0.18
Tumor Stage 1
cor=0.054, p=0.13
Tumor Stage 2
cor=–0.0037, p=0.92
Tumor Stage 3
cor=–0.021, p=0.56
Tumor Stage 4
cor=0.11, p=0.0024
Node Stage 0
cor=–0.085, p=0.019
Node Stage 1
cor=–0.055, p=0.13
Node Stage 2
cor=0.012, p=0.74
Node Stage 3
cor=–0.067, p=0.073
Metastasis Stage 1 (0=N, 1=Y)
cor=–0.017, p=0.64
Path Stage 1
cor=0.057, p=0.12
Path Stage 2
cor=–0.036, p=0.32
Path Stage 3
cor=–0.052, p=0.15
Path Stage4
cor=–0.16, p=1.4e–05
ER Status (0=Neg., 1=Pos.)
cor=–0.12, p=0.0012
PR Status (0=Neg., 1=Pos.)
cor=0.027, p=0.47
Her2 Status (0=Neg., 1=Pos.)
cor=–0.14, p=9.4e–05
AR Levels Exp
Eigengene

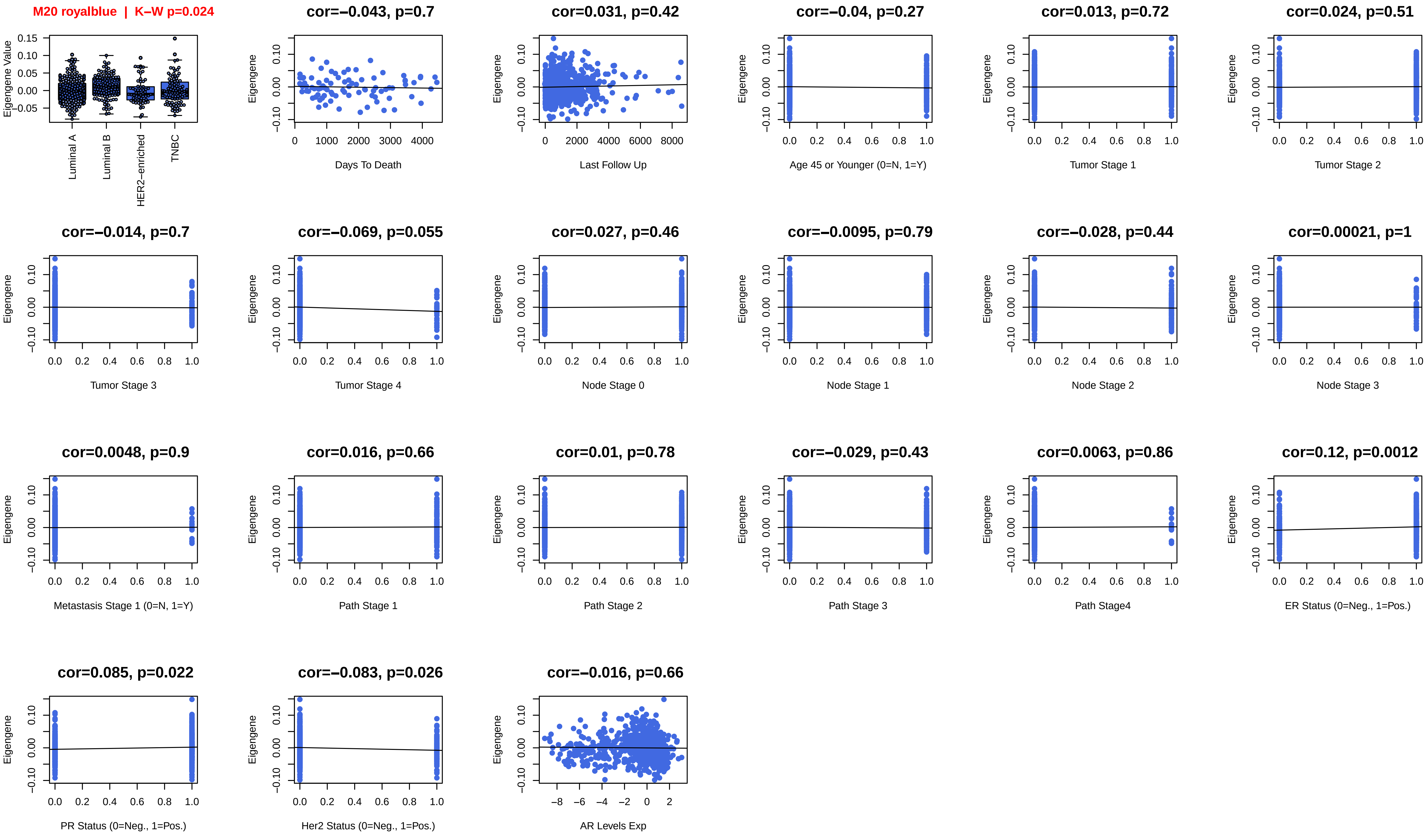
M20 royalblue | K–W p=0.024
Eigengene Value
Luminal A
Luminal B
HER2–enriched
TNBC
cor=–0.043, p=0.7
Days To Death
cor=0.031, p=0.42
Last Follow Up
cor=–0.04, p=0.27
Age 45 or Younger (0=N, 1=Y)
cor=0.013, p=0.72
Tumor Stage 1
cor=0.024, p=0.51
Tumor Stage 2
cor=–0.014, p=0.7
Tumor Stage 3
cor=–0.069, p=0.055
Tumor Stage 4
cor=0.027, p=0.46
Node Stage 0
cor=–0.0095, p=0.79
Node Stage 1
cor=–0.028, p=0.44
Node Stage 2
cor=0.00021, p=1
Node Stage 3
cor=0.0048, p=0.9
Metastasis Stage 1 (0=N, 1=Y)
cor=0.016, p=0.66
Path Stage 1
cor=0.01, p=0.78
Path Stage 2
cor=–0.029, p=0.43
Path Stage 3
cor=0.0063, p=0.86
Path Stage4
cor=0.12, p=0.0012
ER Status (0=Neg., 1=Pos.)
cor=0.085, p=0.022
PR Status (0=Neg., 1=Pos.)
cor=–0.083, p=0.026
Her2 Status (0=Neg., 1=Pos.)
cor=–0.016, p=0.66
AR Levels Exp
Eigengene

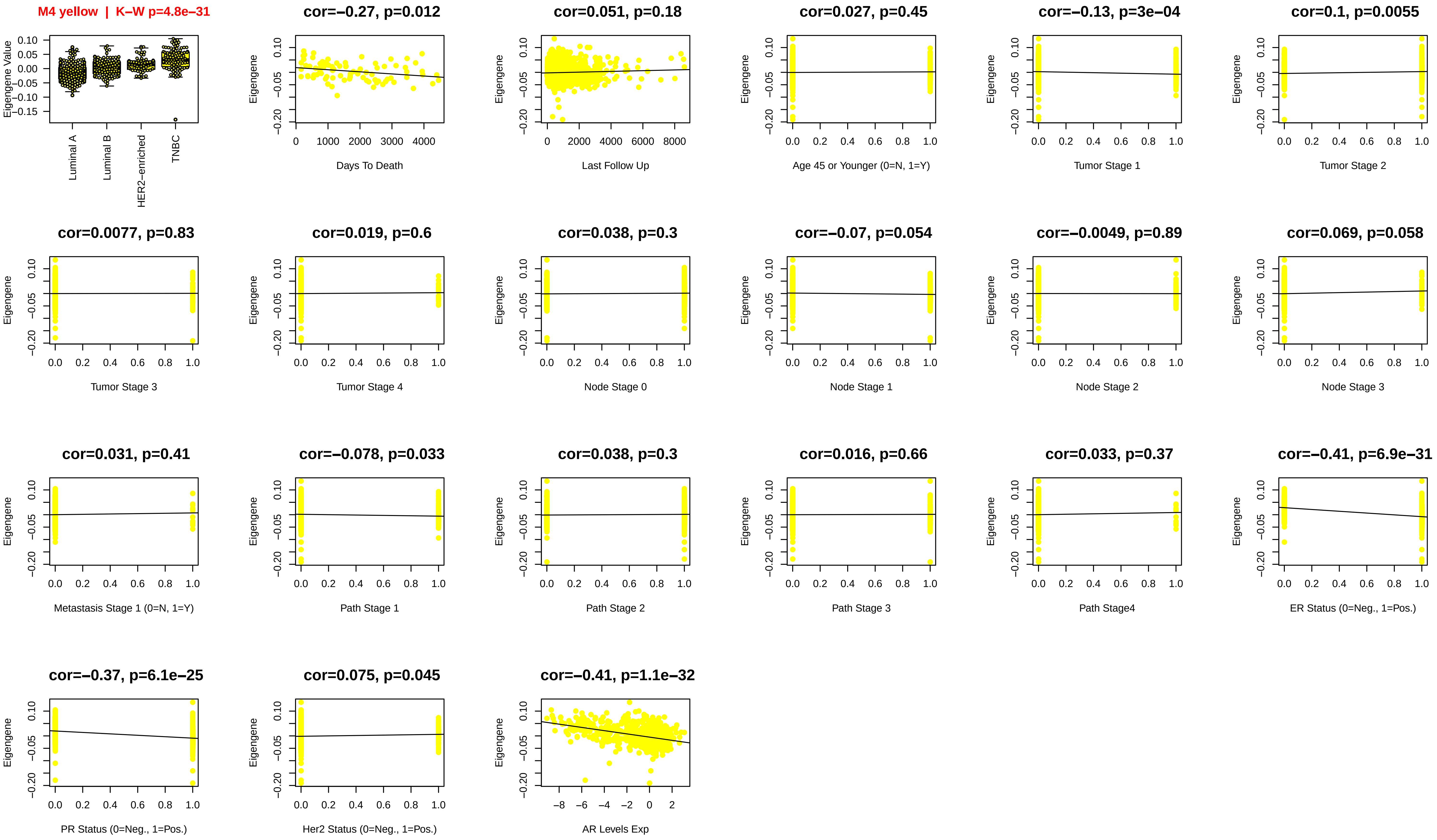
M4 yellow | K–W p=4.8e–31
Eigengene Value
Luminal A
Luminal B
HER2–enriched
TNBC
cor=–0.27, p=0.012
Days To Death
cor=0.051, p=0.18
Last Follow Up
cor=0.027, p=0.45
Age 45 or Younger (0=N, 1=Y)
cor=–0.13, p=3e–04
Tumor Stage 1
cor=0.1, p=0.0055
Tumor Stage 2
cor=0.0077, p=0.83
Tumor Stage 3
cor=0.019, p=0.6
Tumor Stage 4
cor=0.038, p=0.3
Node Stage 0
cor=–0.07, p=0.054
Node Stage 1
cor=–0.0049, p=0.89
Node Stage 2
cor=0.069, p=0.058
Node Stage 3
cor=0.031, p=0.41
Metastasis Stage 1 (0=N, 1=Y)
cor=–0.078, p=0.033
Path Stage 1
cor=0.038, p=0.3
Path Stage 2
cor=0.016, p=0.66
Path Stage 3
cor=0.033, p=0.37
Path Stage4
cor=–0.41, p=6.9e–31
ER Status (0=Neg., 1=Pos.)
cor=–0.37, p=6.1e–25
PR Status (0=Neg., 1=Pos.)
cor=0.075, p=0.045
Her2 Status (0=Neg., 1=Pos.)
cor=–0.41, p=1.1e–32
AR Levels Exp
Eigengene

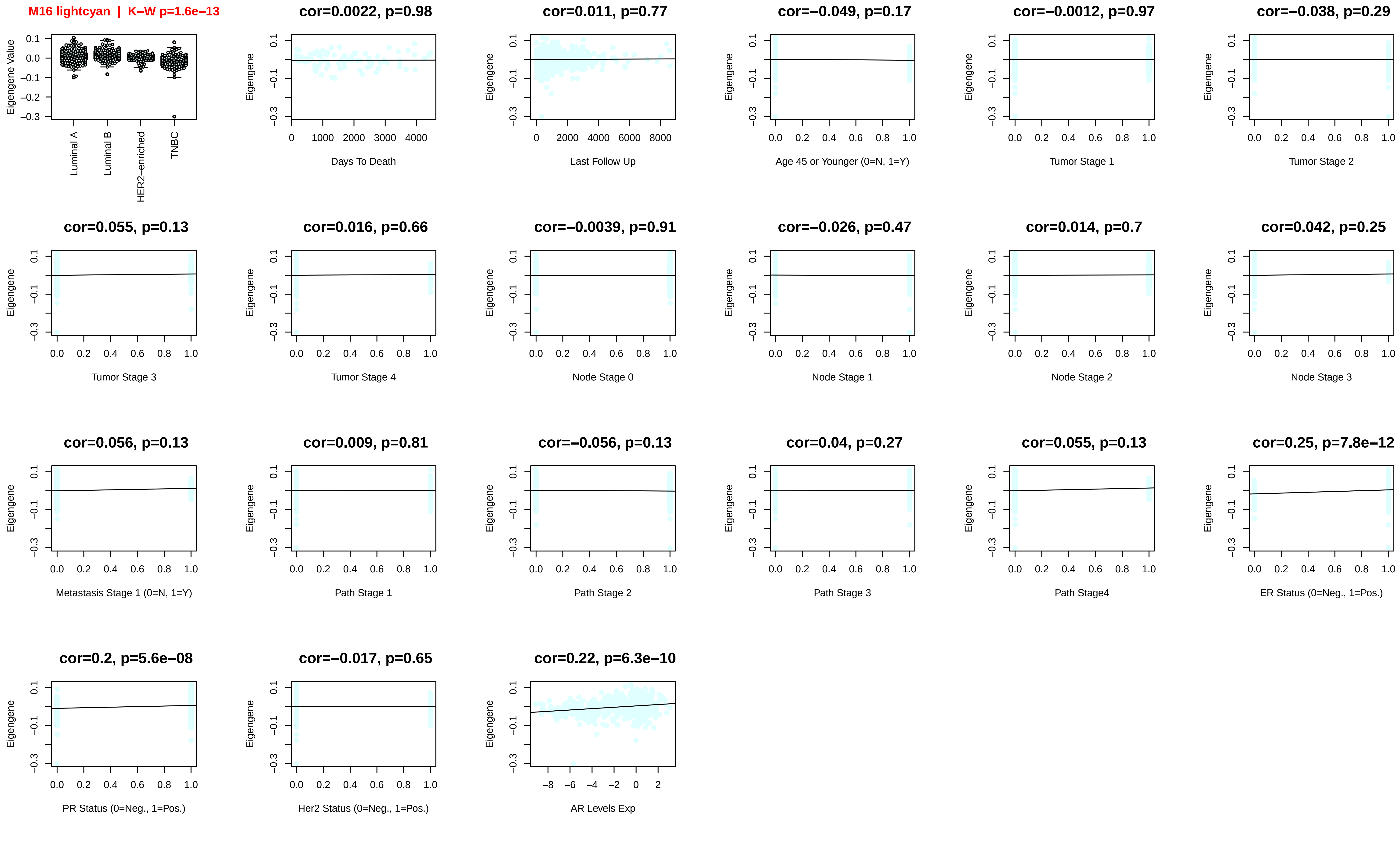
M16 lightcyan | K–W p=1.6e–13
Eigengene Value
Luminal A
Luminal B
HER2–enriched
TNBC
cor=0.0022, p=0.98
Days To Death
cor=0.011, p=0.77
Last Follow Up
cor=–0.049, p=0.17
Age 45 or Younger (0=N, 1=Y)
cor=–0.0012, p=0.97
Tumor Stage 1
cor=–0.038, p=0.29
Tumor Stage 2
cor=0.055, p=0.13
Tumor Stage 3
cor=0.016, p=0.66
Tumor Stage 4
cor=–0.0039, p=0.91
Node Stage 0
cor=–0.026, p=0.47
Node Stage 1
cor=0.014, p=0.7
Node Stage 2
cor=0.042, p=0.25
Node Stage 3
cor=0.056, p=0.13
Metastasis Stage 1 (0=N, 1=Y)
cor=0.009, p=0.81
Path Stage 1
cor=–0.056, p=0.13
Path Stage 2
cor=0.04, p=0.27
Path Stage 3
cor=0.055, p=0.13
Path Stage4
cor=0.25, p=7.8e–12
ER Status (0=Neg., 1=Pos.)
cor=0.2, p=5.6e–08
PR Status (0=Neg., 1=Pos.)
cor=–0.017, p=0.65
Her2 Status (0=Neg., 1=Pos.)
cor=0.22, p=6.3e–10
AR Levels Exp
Eigengene

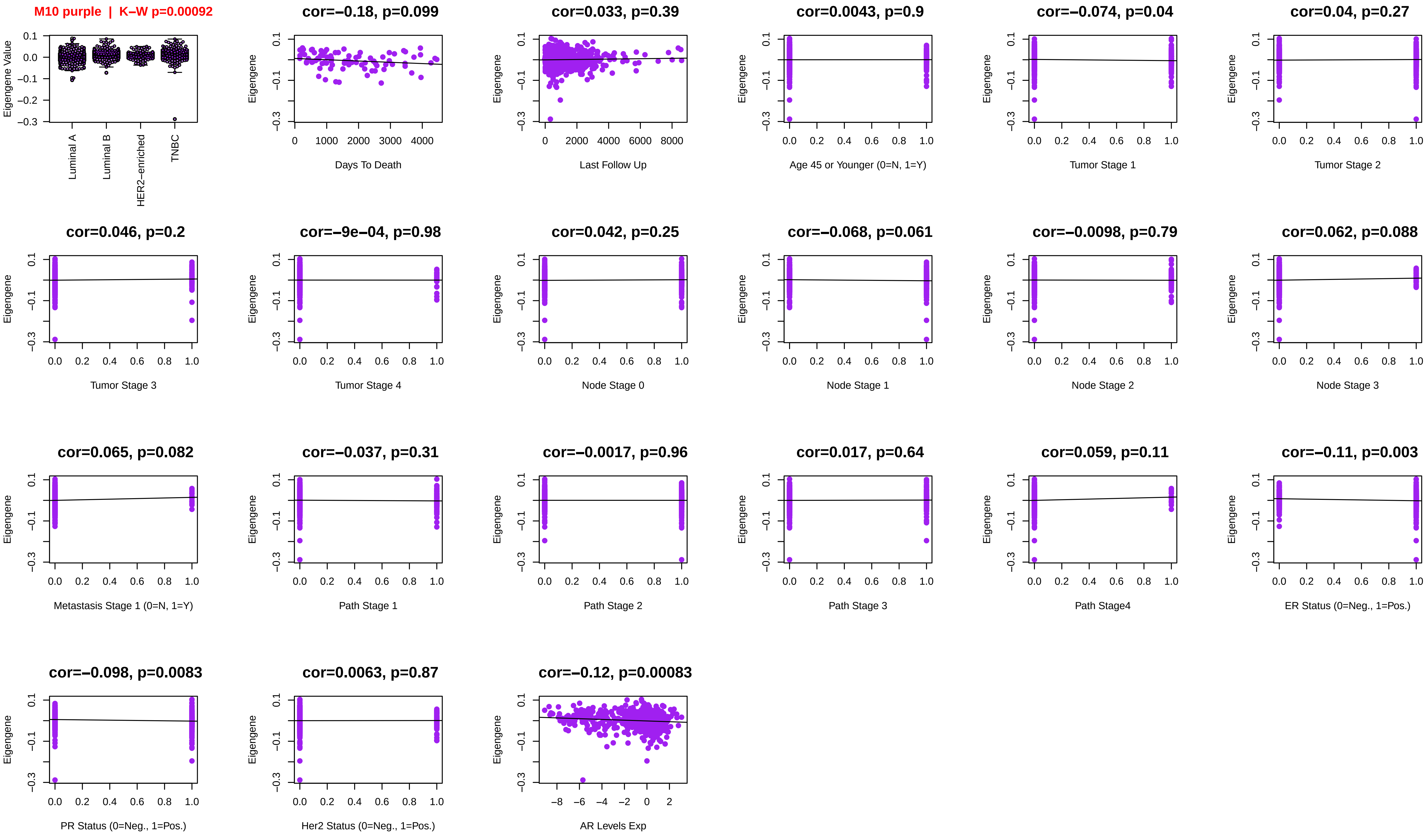
M10 purple | K–W p=0.00092
Eigengene Value
Luminal A
Luminal B
HER2–enriched
TNBC
cor=–0.18, p=0.099
Days To Death
cor=0.033, p=0.39
Last Follow Up
cor=0.0043, p=0.9
Age 45 or Younger (0=N, 1=Y)
cor=–0.074, p=0.04
Tumor Stage 1
cor=0.04, p=0.27
Tumor Stage 2
cor=0.046, p=0.2
Tumor Stage 3
cor=–9e–04, p=0.98
Tumor Stage 4
cor=0.042, p=0.25
Node Stage 0
cor=–0.068, p=0.061
Node Stage 1
cor=–0.0098, p=0.79
Node Stage 2
cor=0.062, p=0.088
Node Stage 3
cor=0.065, p=0.082
Metastasis Stage 1 (0=N, 1=Y)
cor=–0.037, p=0.31
Path Stage 1
cor=–0.0017, p=0.96
Path Stage 2
cor=0.017, p=0.64
Path Stage 3
cor=0.059, p=0.11
Path Stage4
cor=–0.11, p=0.003
ER Status (0=Neg., 1=Pos.)
cor=–0.098, p=0.0083
PR Status (0=Neg., 1=Pos.)
cor=0.0063, p=0.87
Her2 Status (0=Neg., 1=Pos.)
cor=–0.12, p=0.00083
AR Levels Exp
Eigengene

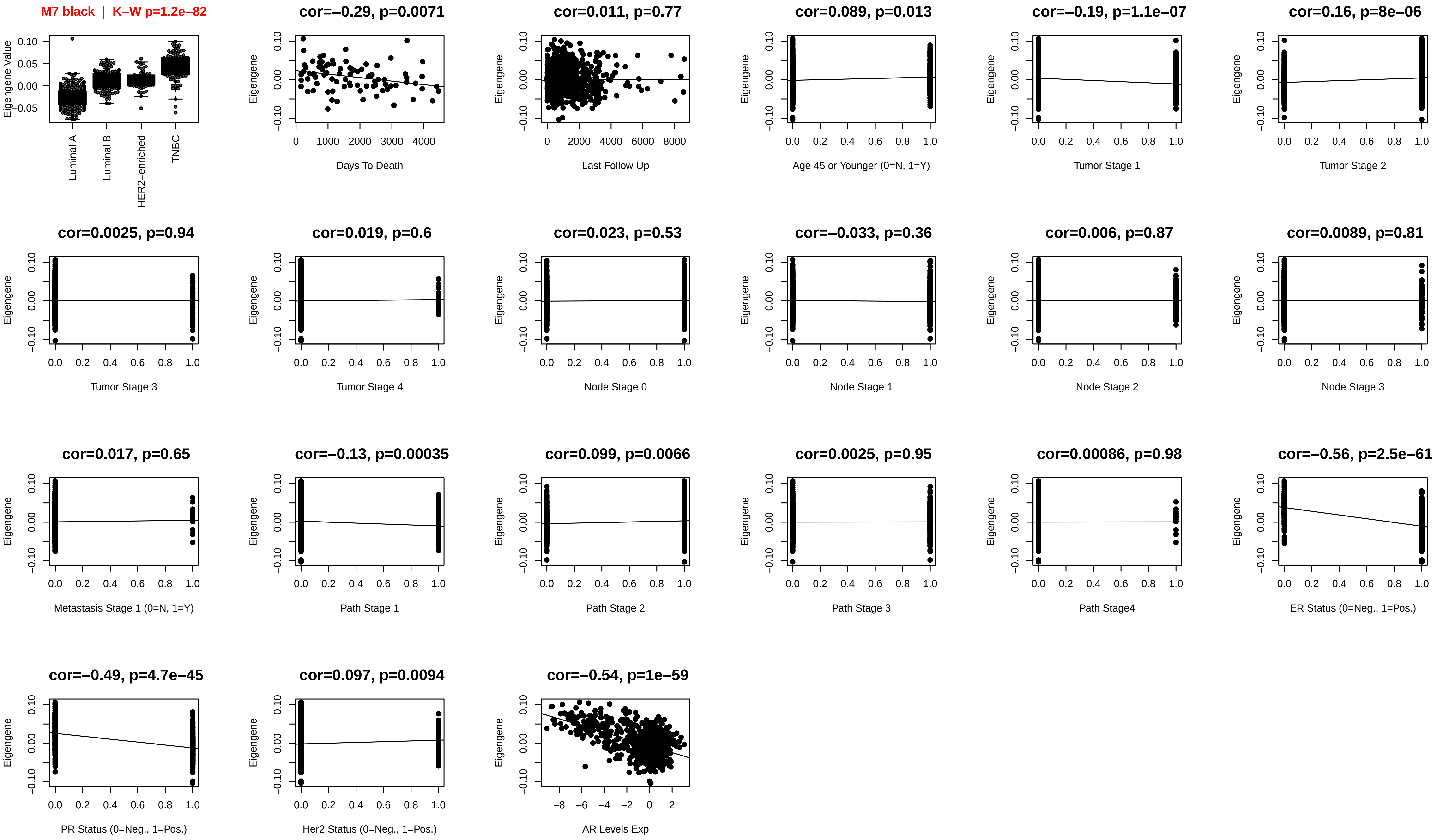
M7 black | K–W p=1.2e–82
Eigengene Value
Luminal A
Luminal B
HER2–enriched
TNBC
cor=–0.29, p=0.0071
Days To Death
cor=0.011, p=0.77
Last Follow Up
cor=0.089, p=0.013
Age 45 or Younger (0=N, 1=Y)
cor=–0.19, p=1.1e–07
Tumor Stage 1
cor=0.16, p=8e–06
Tumor Stage 2
cor=0.0025, p=0.94
Tumor Stage 3
cor=0.019, p=0.6
Tumor Stage 4
cor=0.023, p=0.53
Node Stage 0
cor=–0.033, p=0.36
Node Stage 1
cor=0.006, p=0.87
Node Stage 2
cor=0.0089, p=0.81
Node Stage 3
cor=0.017, p=0.65
Metastasis Stage 1 (0=N, 1=Y)
cor=–0.13, p=0.00035
Path Stage 1
cor=0.099, p=0.0066
Path Stage 2
cor=0.0025, p=0.95
Path Stage 3
cor=0.00086, p=0.98
Path Stage4
cor=–0.56, p=2.5e–61
ER Status (0=Neg., 1=Pos.)
cor=–0.49, p=4.7e–45
PR Status (0=Neg., 1=Pos.)
cor=0.097, p=0.0094
Her2 Status (0=Neg., 1=Pos.)
cor=–0.54, p=1e–59
AR Levels Exp
Eigengene

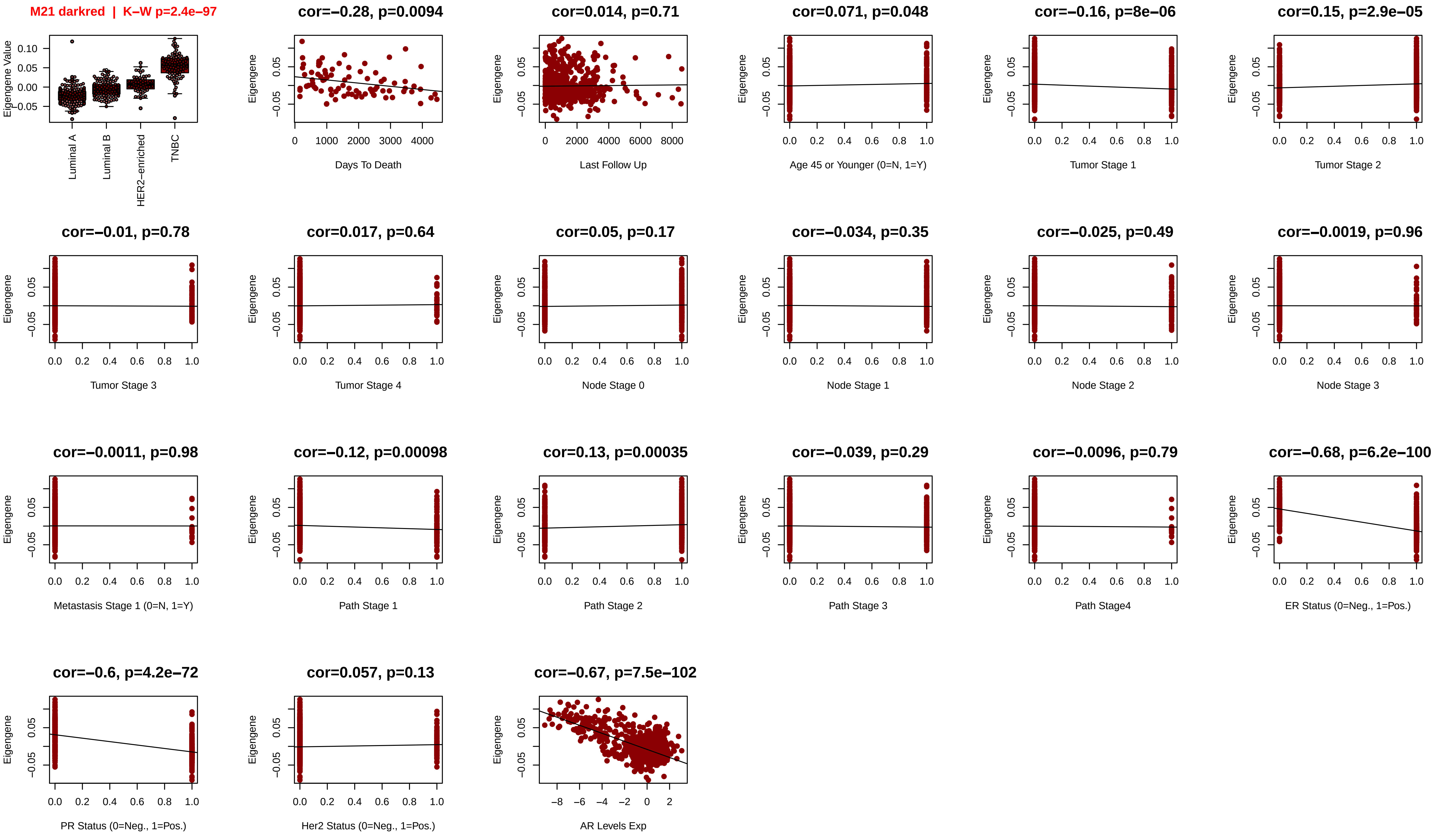
M21 darkred | K–W p=2.4e–97
Eigengene Value
Luminal A
Luminal B
HER2–enriched
TNBC
cor=–0.28, p=0.0094
Days To Death
cor=0.014, p=0.71
Last Follow Up
cor=0.071, p=0.048
Age 45 or Younger (0=N, 1=Y)
cor=–0.16, p=8e–06
Tumor Stage 1
cor=0.15, p=2.9e–05
Tumor Stage 2
cor=–0.01, p=0.78
Tumor Stage 3
cor=0.017, p=0.64
Tumor Stage 4
cor=0.05, p=0.17
Node Stage 0
cor=–0.034, p=0.35
Node Stage 1
cor=–0.025, p=0.49
Node Stage 2
cor=–0.0019, p=0.96
Node Stage 3
cor=–0.0011, p=0.98
Metastasis Stage 1 (0=N, 1=Y)
cor=–0.12, p=0.00098
Path Stage 1
cor=0.13, p=0.00035
Path Stage 2
cor=–0.039, p=0.29
Path Stage 3
cor=–0.0096, p=0.79
Path Stage4
cor=–0.68, p=6.2e–100
ER Status (0=Neg., 1=Pos.)
cor=–0.6, p=4.2e–72
PR Status (0=Neg., 1=Pos.)
cor=0.057, p=0.13
Her2 Status (0=Neg., 1=Pos.)
cor=–0.67, p=7.5e–102
AR Levels Exp
Eigengene

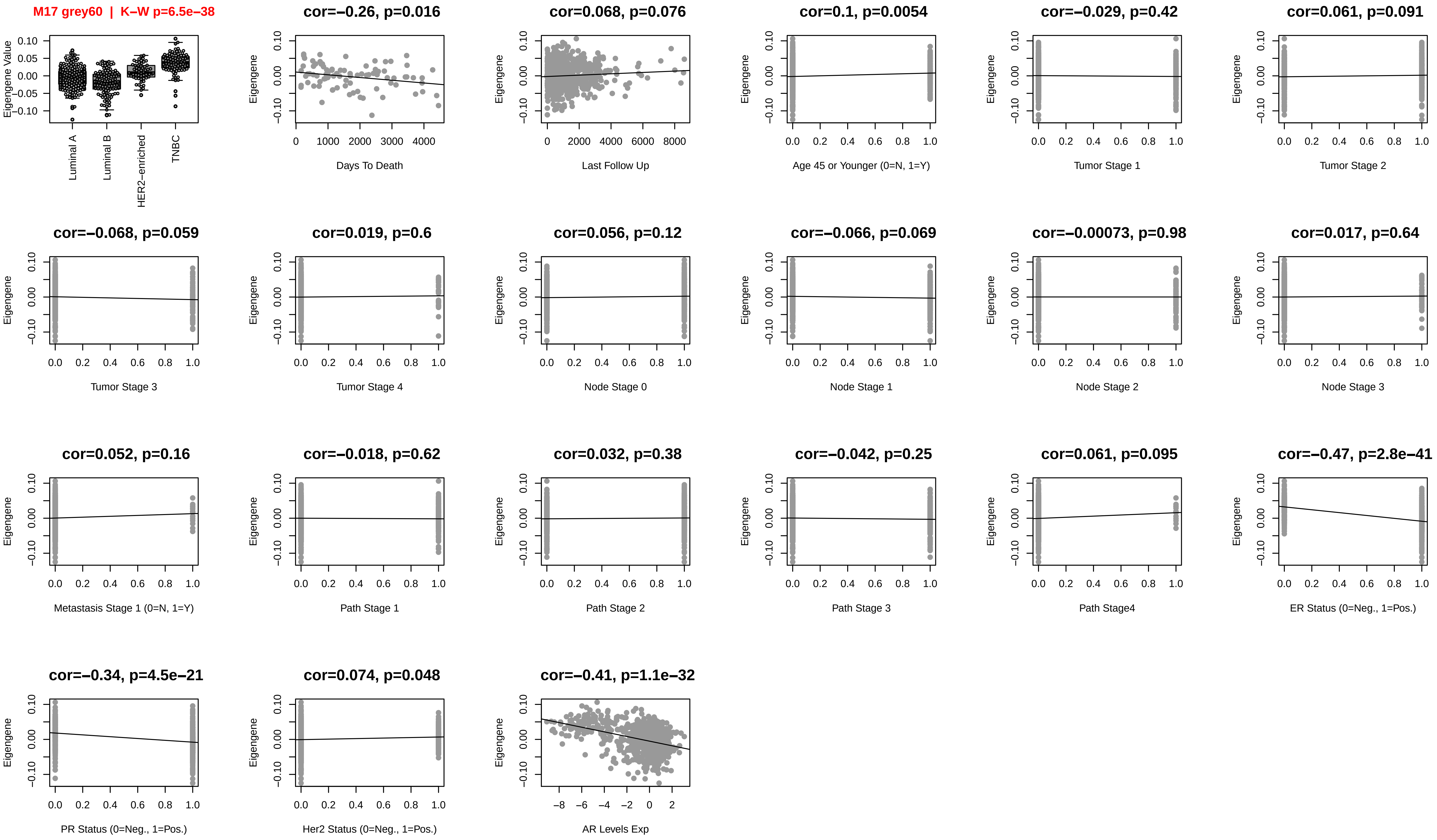
M17 grey60 | K–W p=6.5e–38
Eigengene Value
Luminal A
Luminal B
HER2–enriched
TNBC
cor=–0.26, p=0.016
Days To Death
cor=0.068, p=0.076
Last Follow Up
cor=0.1, p=0.0054
Age 45 or Younger (0=N, 1=Y)
cor=–0.029, p=0.42
Tumor Stage 1
cor=0.061, p=0.091
Tumor Stage 2
cor=–0.068, p=0.059
Tumor Stage 3
cor=0.019, p=0.6
Tumor Stage 4
cor=0.056, p=0.12
Node Stage 0
cor=–0.066, p=0.069
Node Stage 1
cor=–0.00073, p=0.98
Node Stage 2
cor=0.017, p=0.64
Node Stage 3
cor=0.052, p=0.16
Metastasis Stage 1 (0=N, 1=Y)
cor=–0.018, p=0.62
Path Stage 1
cor=0.032, p=0.38
Path Stage 2
cor=–0.042, p=0.25
Path Stage 3
cor=0.061, p=0.095
Path Stage4
cor=–0.47, p=2.8e–41
ER Status (0=Neg., 1=Pos.)
cor=–0.34, p=4.5e–21
PR Status (0=Neg., 1=Pos.)
cor=0.074, p=0.048
Her2 Status (0=Neg., 1=Pos.)
cor=–0.41, p=1.1e–32
AR Levels Exp
Eigengene

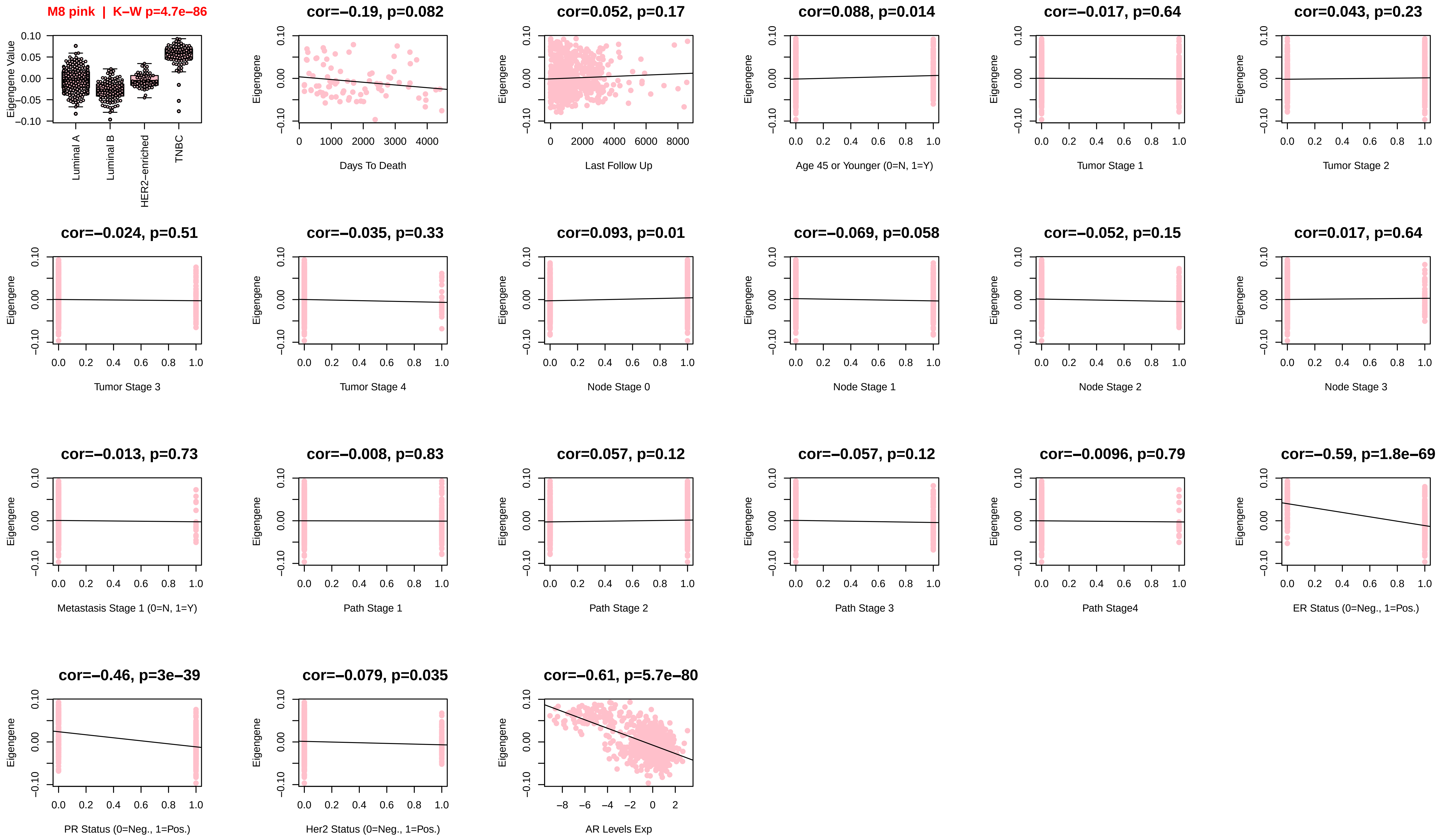

M8 pink | K–W p=4.7e–86
Eigengene Value
Luminal A
Luminal B
HER2–enriched
TNBC
cor=–0.19, p=0.082
Days To Death
cor=0.052, p=0.17
Last Follow Up
cor=0.088, p=0.014
Age 45 or Younger (0=N, 1=Y)
cor=–0.017, p=0.64
Tumor Stage 1
cor=0.043, p=0.23
Tumor Stage 2
cor=–0.024, p=0.51
Tumor Stage 3
cor=–0.035, p=0.33
Tumor Stage 4
cor=0.093, p=0.01
Node Stage 0
cor=–0.069, p=0.058
Node Stage 1
cor=–0.052, p=0.15
Node Stage 2
cor=0.017, p=0.64
Node Stage 3
cor=–0.013, p=0.73
Metastasis Stage 1 (0=N, 1=Y)
cor=–0.008, p=0.83
Path Stage 1
cor=0.057, p=0.12
Path Stage 2
cor=–0.057, p=0.12
Path Stage 3
cor=–0.0096, p=0.79
Path Stage4
cor=–0.59, p=1.8e–69
ER Status (0=Neg., 1=Pos.)
cor=–0.46, p=3e–39
PR Status (0=Neg., 1=Pos.)
cor=–0.079, p=0.035
Her2 Status (0=Neg., 1=Pos.)
cor=–0.61, p=5.7e–80
AR Levels Exp
Eigengene

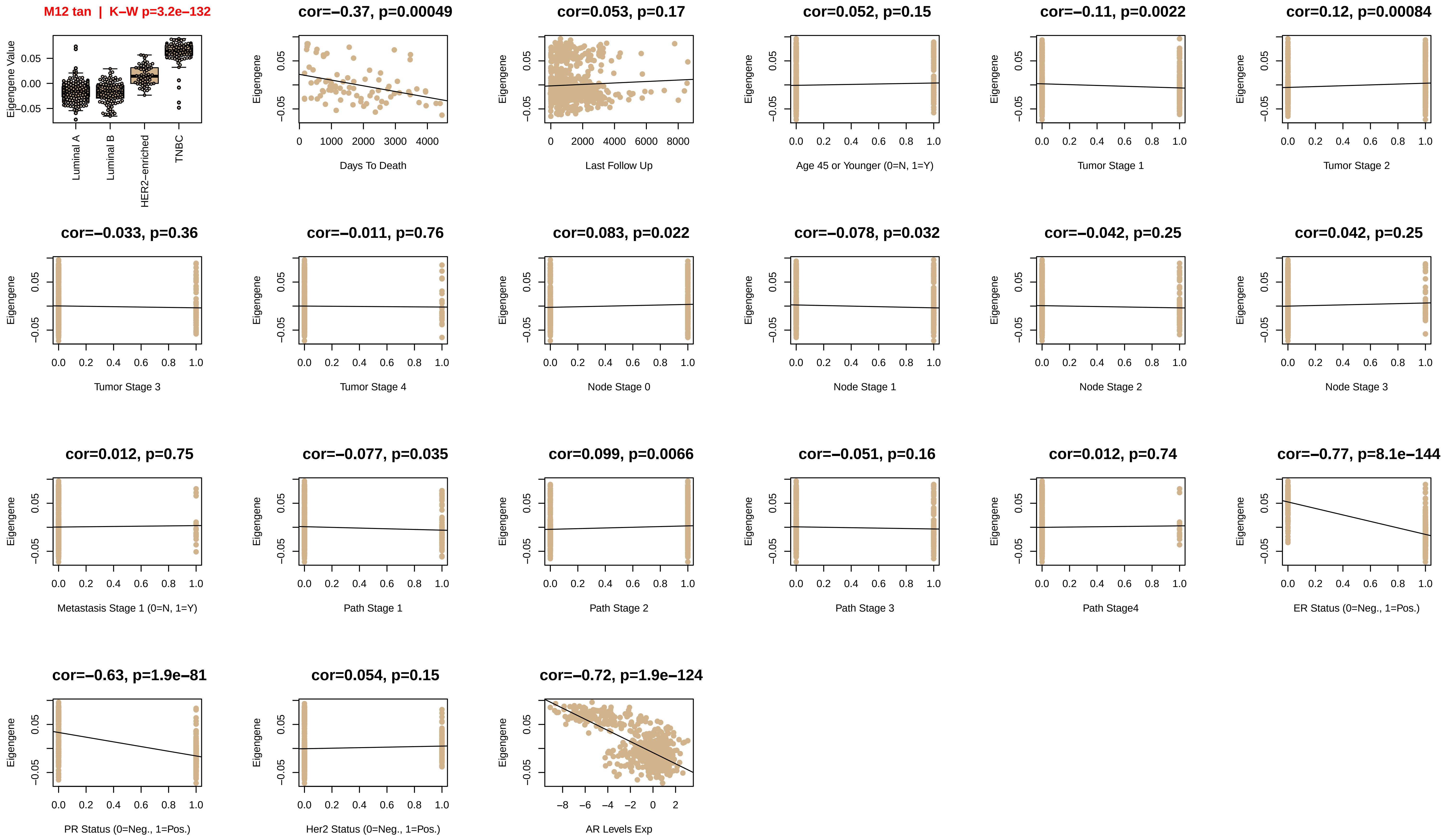

M12 tan | K–W p=3.2e–132
Eigengene Value
Luminal A
Luminal B
HER2–enriched
TNBC
cor=–0.37, p=0.00049
Days To Death
cor=0.053, p=0.17
Last Follow Up
cor=0.052, p=0.15
Age 45 or Younger (0=N, 1=Y)
cor=–0.11, p=0.0022
Tumor Stage 1
cor=0.12, p=0.00084
Tumor Stage 2
cor=–0.033, p=0.36
Tumor Stage 3
cor=–0.011, p=0.76
Tumor Stage 4
cor=0.083, p=0.022
Node Stage 0
cor=–0.078, p=0.032
Node Stage 1
cor=–0.042, p=0.25
Node Stage 2
cor=0.042, p=0.25
Node Stage 3
cor=0.012, p=0.75
Metastasis Stage 1 (0=N, 1=Y)
cor=–0.077, p=0.035
Path Stage 1
cor=0.099, p=0.0066
Path Stage 2
cor=–0.051, p=0.16
Path Stage 3
cor=0.012, p=0.74
Path Stage4
cor=–0.77, p=8.1e–144
ER Status (0=Neg., 1=Pos.)
cor=–0.63, p=1.9e–81
PR Status (0=Neg., 1=Pos.)
cor=0.054, p=0.15
Her2 Status (0=Neg., 1=Pos.)
cor=–0.72, p=1.9e–124
AR Levels Exp
Eigengene

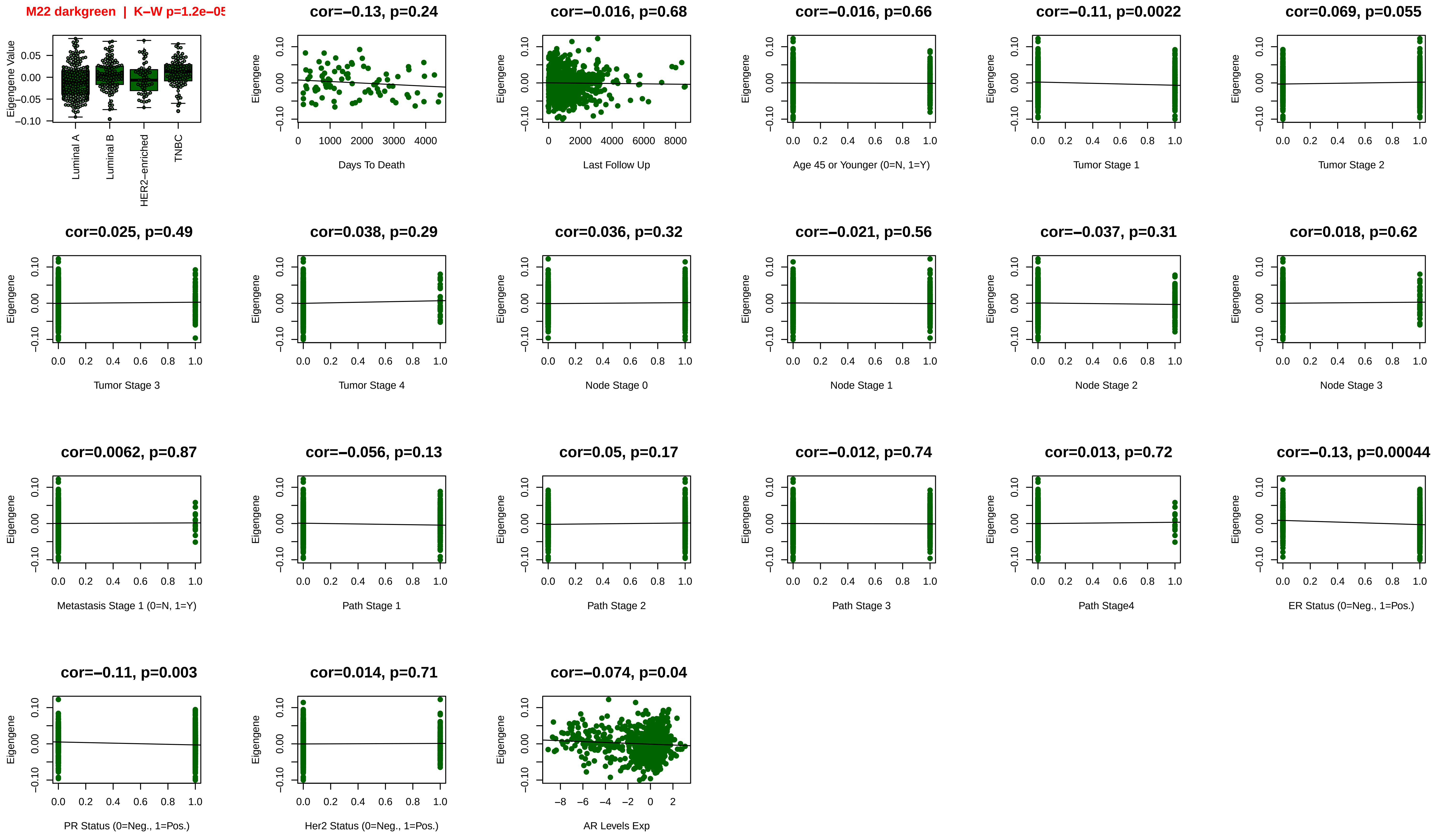

M22 darkgreen | K–W p=1.2e–05
Eigengene Value
Luminal A
Luminal B
HER2–enriched
TNBC
cor=–0.13, p=0.24
Days To Death
cor=–0.016, p=0.68
Last Follow Up
cor=–0.016, p=0.66
Age 45 or Younger (0=N, 1=Y)
cor=–0.11, p=0.0022
Tumor Stage 1
cor=0.069, p=0.055
Tumor Stage 2
cor=0.025, p=0.49
Tumor Stage 3
cor=0.038, p=0.29
Tumor Stage 4
cor=0.036, p=0.32
Node Stage 0
cor=–0.021, p=0.56
Node Stage 1
cor=–0.037, p=0.31
Node Stage 2
cor=0.018, p=0.62
Node Stage 3
cor=0.0062, p=0.87
Metastasis Stage 1 (0=N, 1=Y)
cor=–0.056, p=0.13
Path Stage 1
cor=0.05, p=0.17
Path Stage 2
cor=–0.012, p=0.74
Path Stage 3
cor=0.013, p=0.72
Path Stage4
cor=–0.13, p=0.00044
ER Status (0=Neg., 1=Pos.)
cor=–0.11, p=0.003
PR Status (0=Neg., 1=Pos.)
cor=0.014, p=0.71
Her2 Status (0=Neg., 1=Pos.)
cor=–0.074, p=0.04
AR Levels Exp
Eigengene

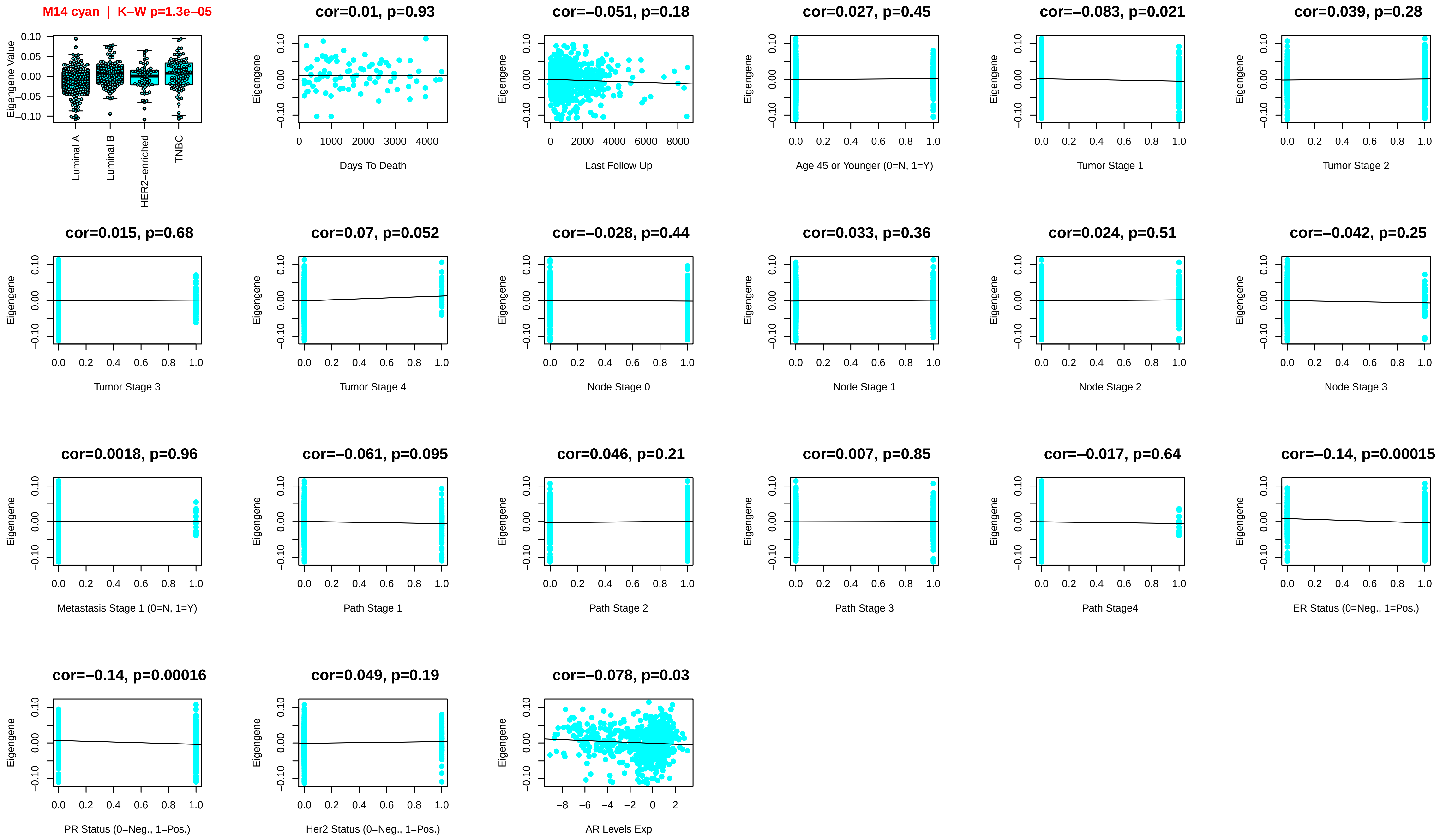
M14 cyan | K−W p=1.3e−05
Eigengene Value
Luminal A
Luminal B
HER2−enriched
TNBC
cor=0.01, p=0.93
Days To Death
cor=−0.051, p=0.18
Last Follow Up
cor=0.027, p=0.45
Age 45 or Younger (0=N, 1=Y)
cor=−0.083, p=0.021
Tumor Stage 1
cor=0.039, p=0.28
Tumor Stage 2
cor=0.015, p=0.68
Tumor Stage 3
cor=0.07, p=0.052
Tumor Stage 4
cor=−0.028, p=0.44
Node Stage 0
cor=0.033, p=0.36
Node Stage 1
cor=0.024, p=0.51
Node Stage 2
cor=−0.042, p=0.25
Node Stage 3
cor=0.0018, p=0.96
Metastasis Stage 1 (0=N, 1=Y)
cor=−0.061, p=0.095
Path Stage 1
cor=0.046, p=0.21
Path Stage 2
cor=0.007, p=0.85
Path Stage 3
cor=−0.017, p=0.64
Path Stage4
cor=−0.14, p=0.00015
ER Status (0=Neg., 1=Pos.)
cor=−0.14, p=0.00016
PR Status (0=Neg., 1=Pos.)
cor=0.049, p=0.19
Her2 Status (0=Neg., 1=Pos.)
cor=−0.078, p=0.03
AR Levels Exp
Eigengene

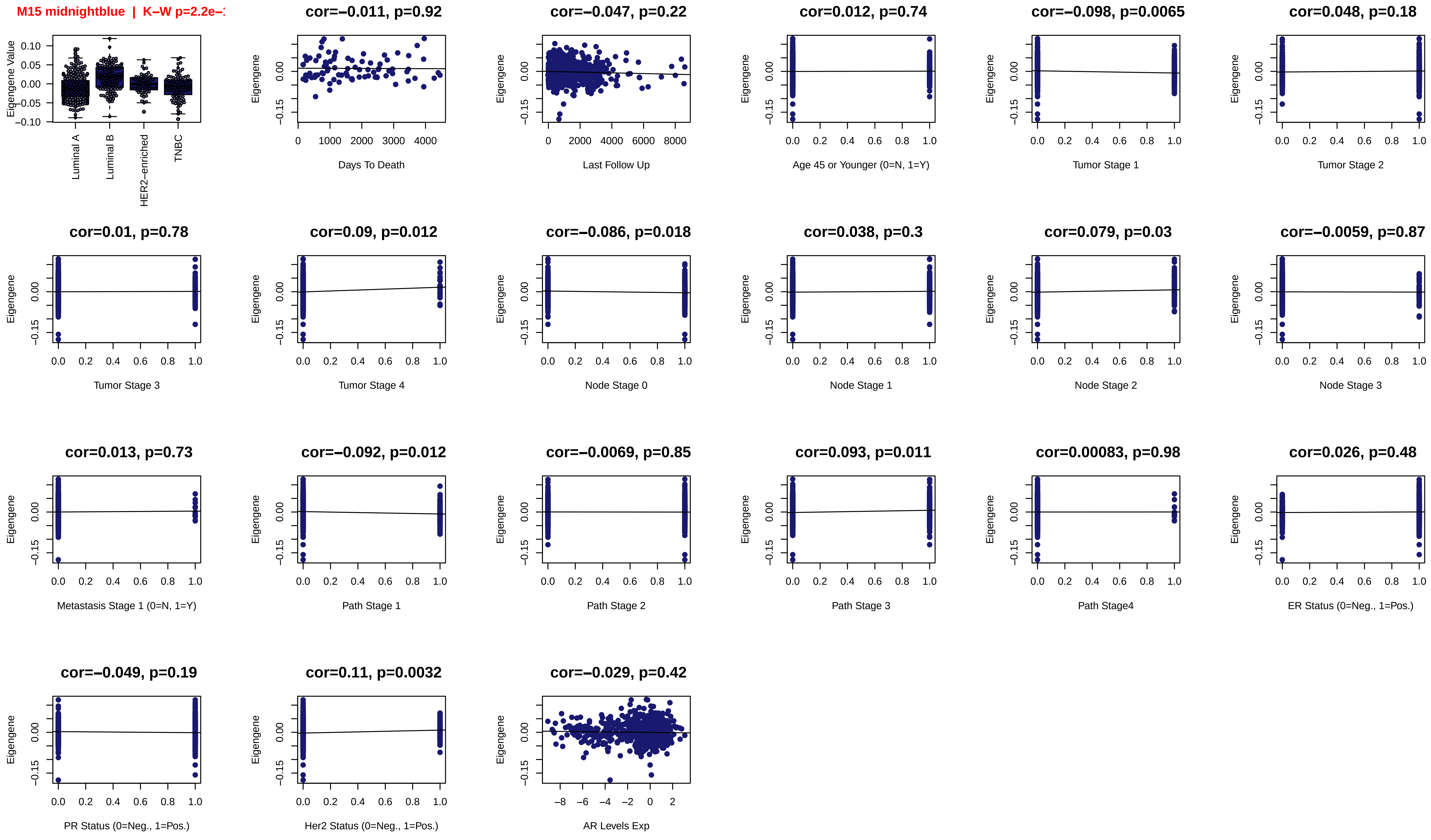
M15 midnightblue | K–W p=2.2e–1
Eigengene Value
Luminal A
Luminal B
HER2–enriched
TNBC
cor=–0.011, p=0.92
Days To Death
cor=–0.047, p=0.22
Last Follow Up
cor=0.012, p=0.74
Age 45 or Younger (0=N, 1=Y)
cor=–0.098, p=0.0065
Tumor Stage 1
cor=0.048, p=0.18
Tumor Stage 2
cor=0.01, p=0.78
Tumor Stage 3
cor=0.09, p=0.012
Tumor Stage 4
cor=–0.086, p=0.018
Node Stage 0
cor=0.038, p=0.3
Node Stage 1
cor=0.079, p=0.03
Node Stage 2
cor=–0.0059, p=0.87
Node Stage 3
cor=0.013, p=0.73
Metastasis Stage 1 (0=N, 1=Y)
cor=–0.092, p=0.012
Path Stage 1
cor=–0.0069, p=0.85
Path Stage 2
cor=0.093, p=0.011
Path Stage 3
cor=0.00083, p=0.98
Path Stage4
cor=0.026, p=0.48
ER Status (0=Neg., 1=Pos.)
cor=–0.049, p=0.19
PR Status (0=Neg., 1=Pos.)
cor=0.11, p=0.0032
Her2 Status (0=Neg., 1=Pos.)
cor=–0.029, p=0.42
AR Levels Exp
Eigengene

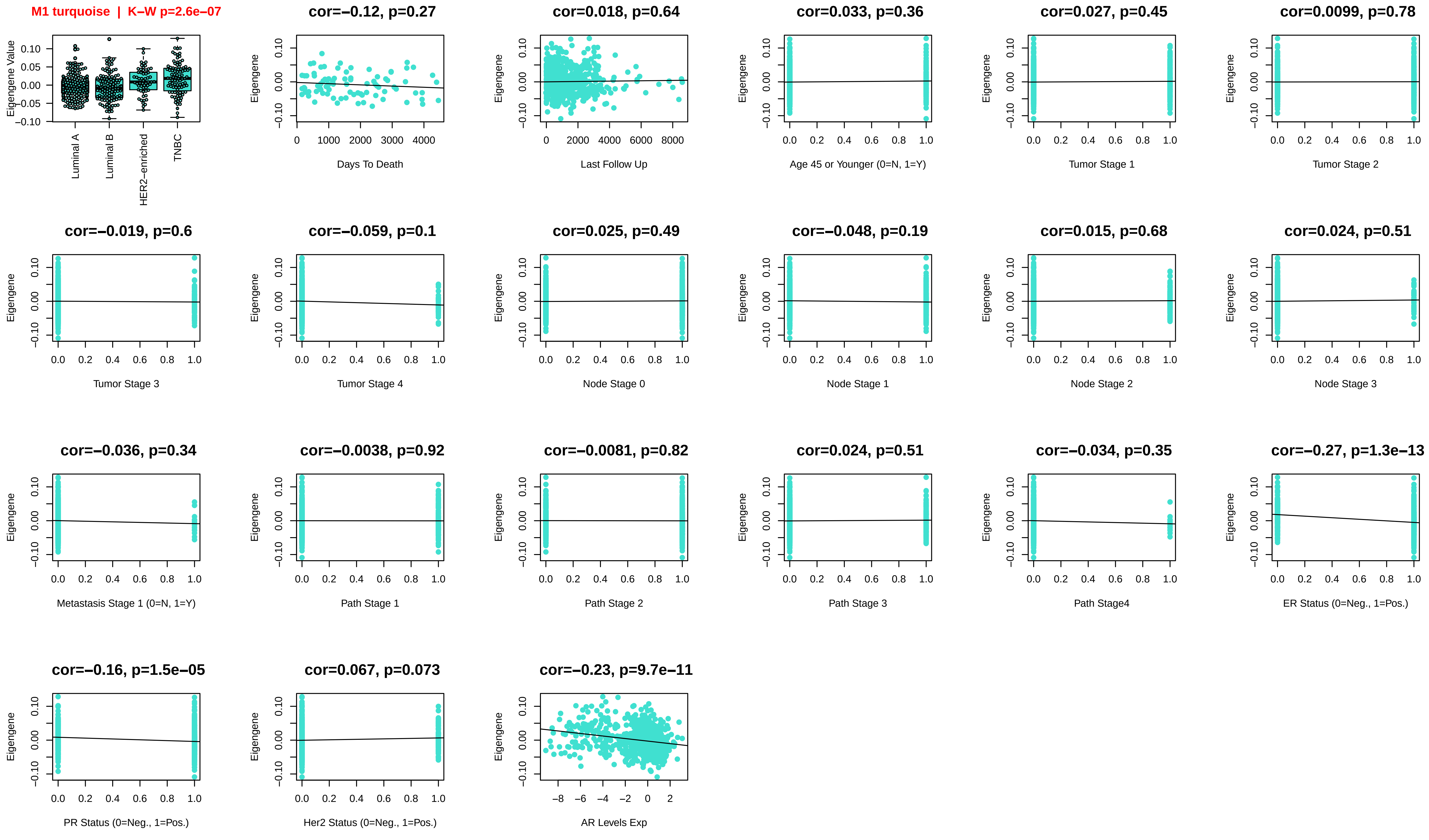
M1 turquoise | K–W p=2.6e–07
Eigengene Value
Luminal A
Luminal B
HER2–enriched
TNBC
cor=–0.12, p=0.27
Days To Death
cor=0.018, p=0.64
Last Follow Up
cor=0.033, p=0.36
Age 45 or Younger (0=N, 1=Y)
cor=0.027, p=0.45
Tumor Stage 1
cor=0.0099, p=0.78
Tumor Stage 2
cor=–0.019, p=0.6
Tumor Stage 3
cor=–0.059, p=0.1
Tumor Stage 4
cor=0.025, p=0.49
Node Stage 0
cor=–0.048, p=0.19
Node Stage 1
cor=0.015, p=0.68
Node Stage 2
cor=0.024, p=0.51
Node Stage 3
cor=–0.036, p=0.34
Metastasis Stage 1 (0=N, 1=Y)
cor=–0.0038, p=0.92
Path Stage 1
cor=–0.0081, p=0.82
Path Stage 2
cor=0.024, p=0.51
Path Stage 3
cor=–0.034, p=0.35
Path Stage4
cor=–0.27, p=1.3e–13
ER Status (0=Neg., 1=Pos.)
cor=–0.16, p=1.5e–05
PR Status (0=Neg., 1=Pos.)
cor=0.067, p=0.073
Her2 Status (0=Neg., 1=Pos.)
cor=–0.23, p=9.7e–11
AR Levels Exp
Eigengene

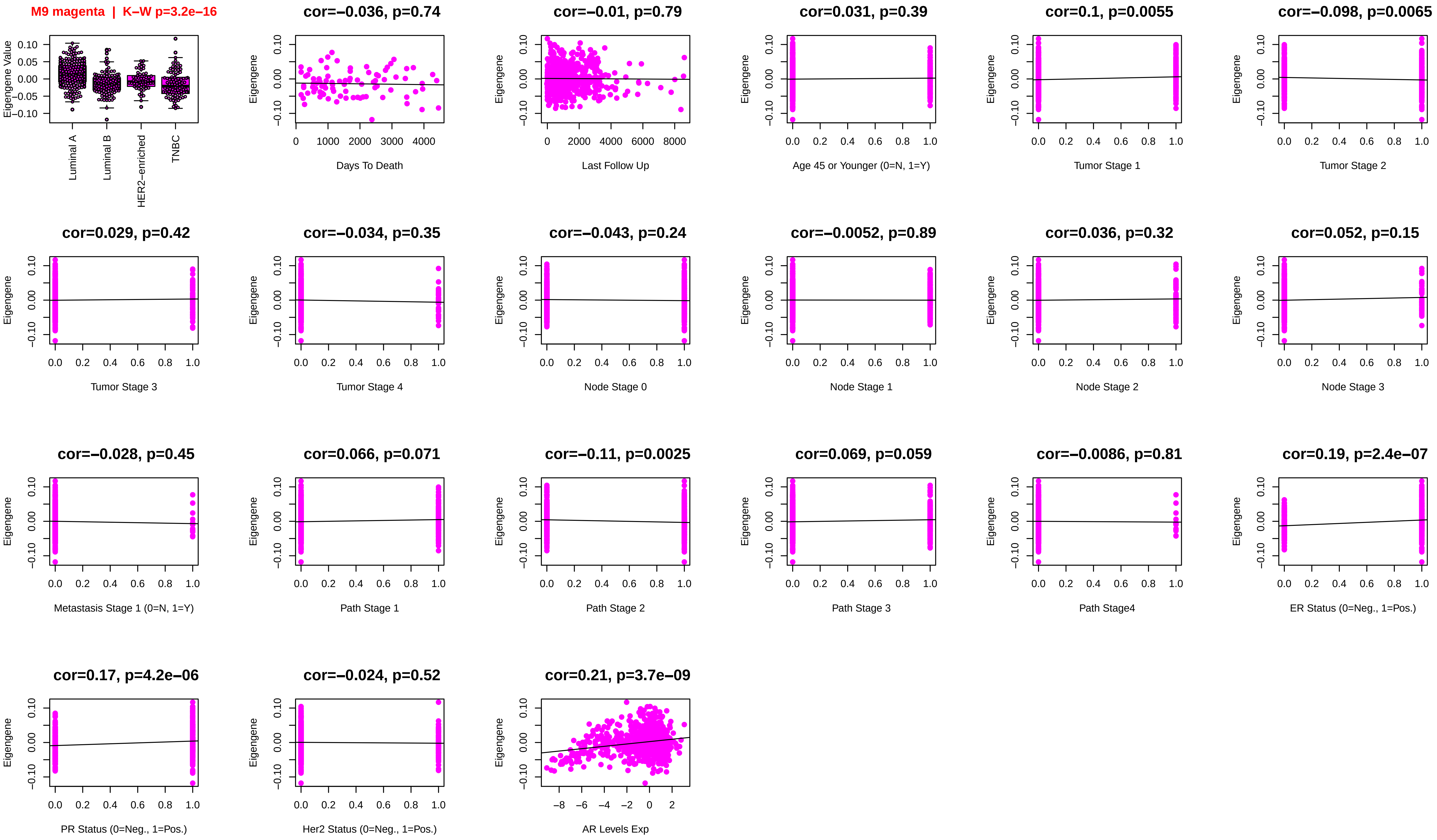
M9 magenta | K–W p=3.2e–16
Eigengene Value
Luminal A
Luminal B
HER2–enriched
TNBC
cor=–0.036, p=0.74
Days To Death
cor=–0.01, p=0.79
Last Follow Up
cor=0.031, p=0.39
Age 45 or Younger (0=N, 1=Y)
cor=0.1, p=0.0055
Tumor Stage 1
cor=–0.098, p=0.0065
Tumor Stage 2
cor=0.029, p=0.42
Tumor Stage 3
cor=–0.034, p=0.35
Tumor Stage 4
cor=–0.043, p=0.24
Node Stage 0
cor=–0.0052, p=0.89
Node Stage 1
cor=0.036, p=0.32
Node Stage 2
cor=0.052, p=0.15
Node Stage 3
cor=–0.028, p=0.45
Metastasis Stage 1 (0=N, 1=Y)
cor=0.066, p=0.071
Path Stage 1
cor=–0.11, p=0.0025
Path Stage 2
cor=0.069, p=0.059
Path Stage 3
cor=–0.0086, p=0.81
Path Stage4
cor=0.19, p=2.4e–07
ER Status (0=Neg., 1=Pos.)
cor=0.17, p=4.2e–06
PR Status (0=Neg., 1=Pos.)
cor=–0.024, p=0.52
Her2 Status (0=Neg., 1=Pos.)
cor=0.21, p=3.7e–09
AR Levels Exp
Eigengene

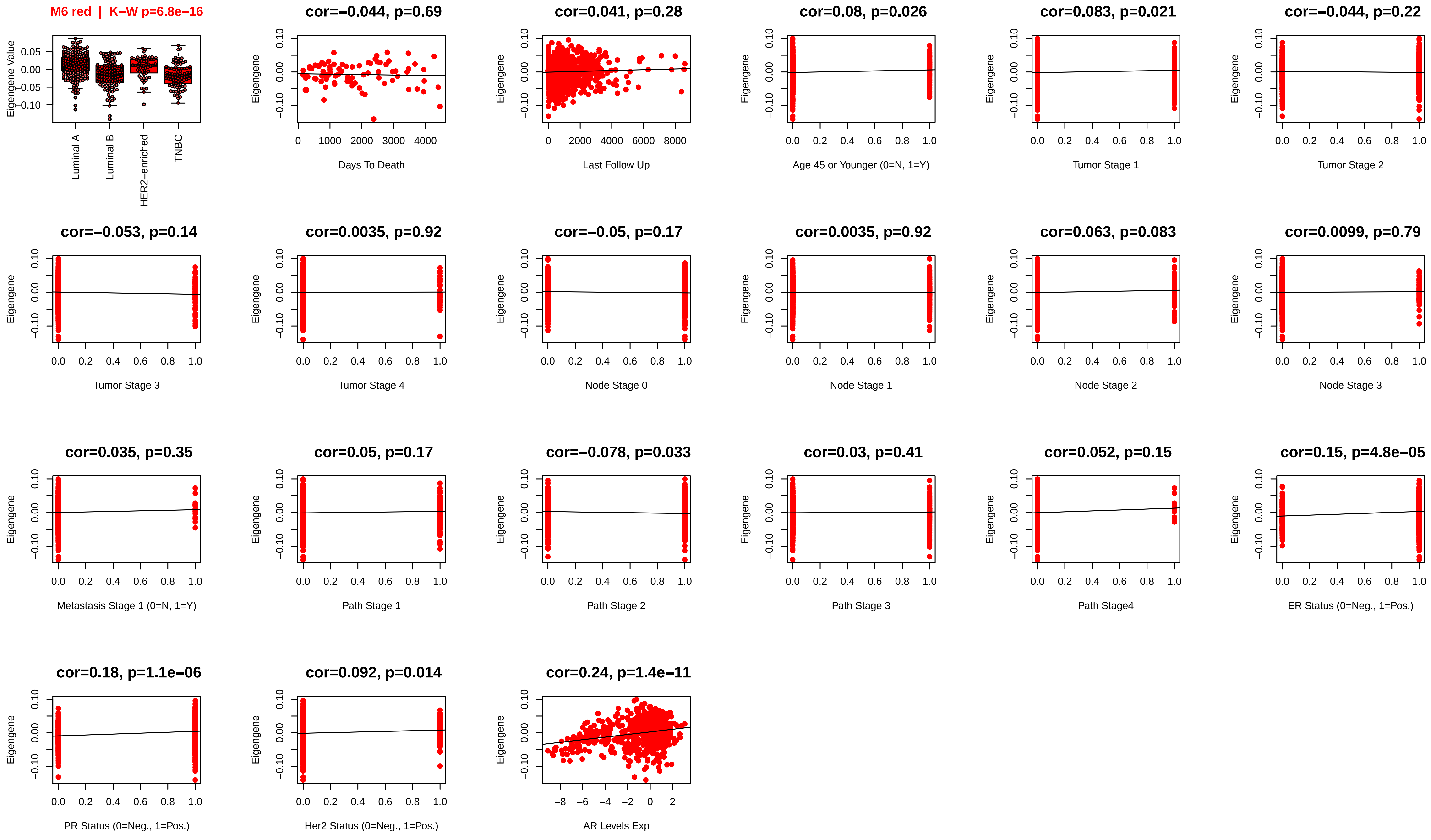
M6 red | K–W p=6.8e–16
Eigengene Value
Luminal A
Luminal B
HER2–enriched
TNBC
cor=–0.044, p=0.69
Days To Death
cor=0.041, p=0.28
Last Follow Up
cor=0.08, p=0.026
Age 45 or Younger (0=N, 1=Y)
cor=0.083, p=0.021
Tumor Stage 1
cor=–0.044, p=0.22
Tumor Stage 2
cor=–0.053, p=0.14
Tumor Stage 3
cor=0.0035, p=0.92
Tumor Stage 4
cor=–0.05, p=0.17
Node Stage 0
cor=0.0035, p=0.92
Node Stage 1
cor=0.063, p=0.083
Node Stage 2
cor=0.0099, p=0.79
Node Stage 3
cor=0.035, p=0.35
Metastasis Stage 1 (0=N, 1=Y)
cor=0.05, p=0.17
Path Stage 1
cor=–0.078, p=0.033
Path Stage 2
cor=0.03, p=0.41
Path Stage 3
cor=0.052, p=0.15
Path Stage4
cor=0.15, p=4.8e–05
ER Status (0=Neg., 1=Pos.)
cor=0.18, p=1.1e–06
PR Status (0=Neg., 1=Pos.)
cor=0.092, p=0.014
Her2 Status (0=Neg., 1=Pos.)
cor=0.24, p=1.4e–11
AR Levels Exp
Eigengene

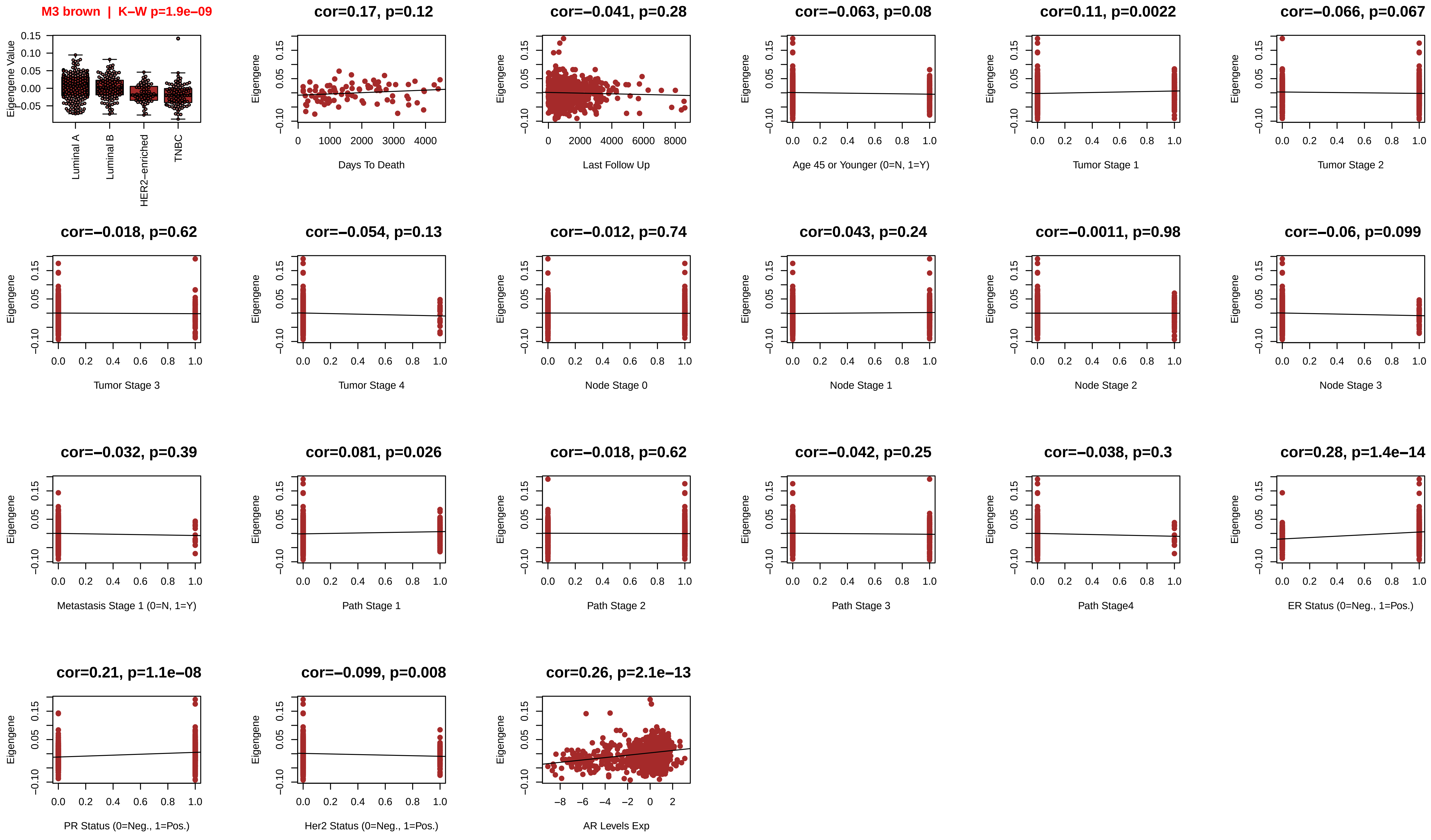
M3 brown | K–W p=1.9e–09
Eigengene Value
Luminal A
Luminal B
HER2–enriched
TNBC
cor=0.17, p=0.12
Days To Death
cor=–0.041, p=0.28
Last Follow Up
cor=–0.063, p=0.08
Age 45 or Younger (0=N, 1=Y)
cor=0.11, p=0.0022
Tumor Stage 1
cor=–0.066, p=0.067
Tumor Stage 2
cor=–0.018, p=0.62
Tumor Stage 3
cor=–0.054, p=0.13
Tumor Stage 4
cor=–0.012, p=0.74
Node Stage 0
cor=0.043, p=0.24
Node Stage 1
cor=–0.0011, p=0.98
Node Stage 2
cor=–0.06, p=0.099
Node Stage 3
cor=–0.032, p=0.39
Metastasis Stage 1 (0=N, 1=Y)
cor=0.081, p=0.026
Path Stage 1
cor=–0.018, p=0.62
Path Stage 2
cor=–0.042, p=0.25
Path Stage 3
cor=–0.038, p=0.3
Path Stage4
cor=0.28, p=1.4e–14
ER Status (0=Neg., 1=Pos.)
cor=0.21, p=1.1e–08
PR Status (0=Neg., 1=Pos.)
cor=–0.099, p=0.008
Her2 Status (0=Neg., 1=Pos.)
cor=0.26, p=2.1e–13
AR Levels Exp
Eigengene

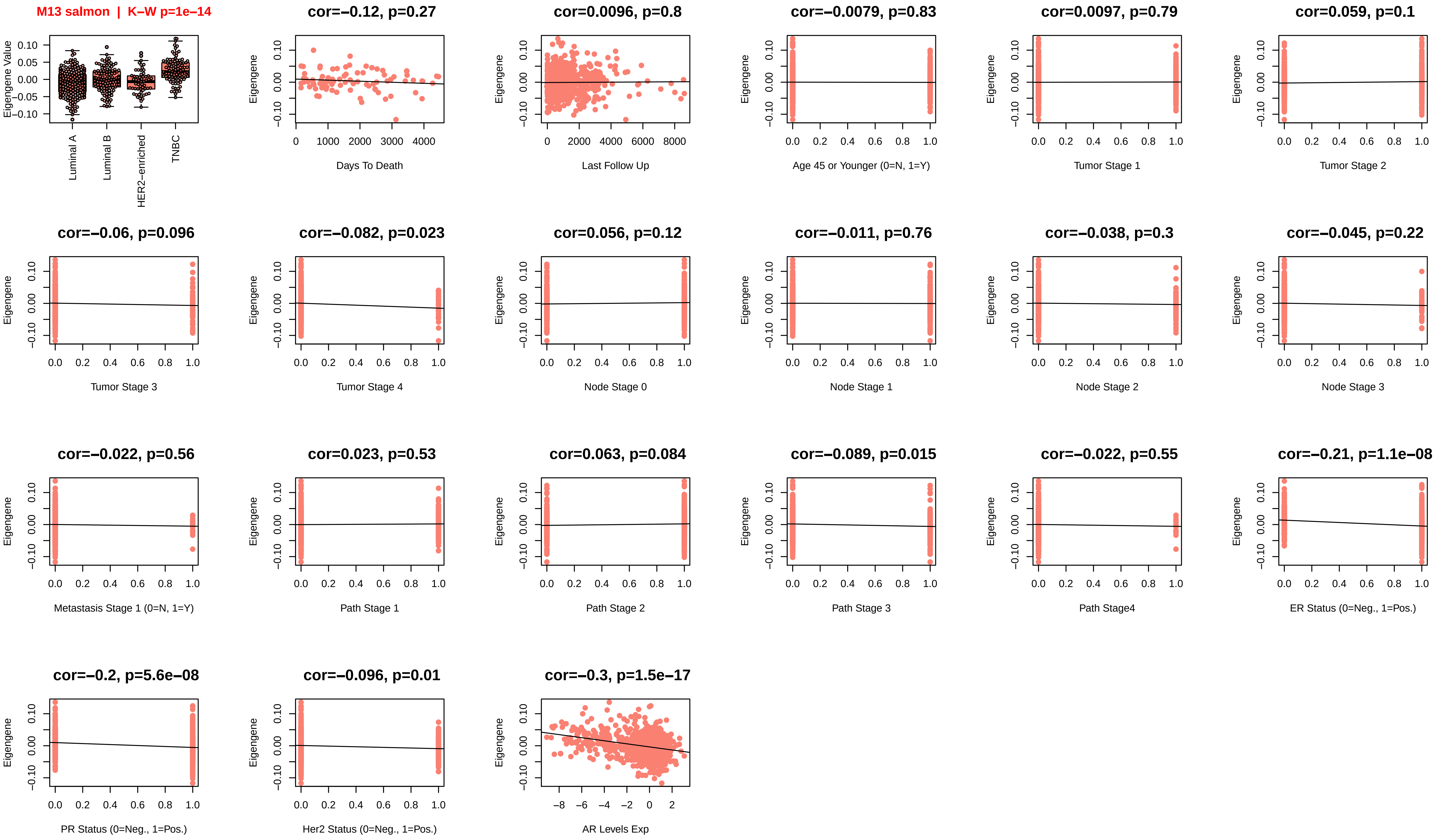
M13 salmon | K–W p=1e–14
Eigengene Value
Luminal A
Luminal B
HER2–enriched
TNBC
cor=–0.12, p=0.27
Days To Death
cor=0.0096, p=0.8
Last Follow Up
cor=–0.0079, p=0.83
Age 45 or Younger (0=N, 1=Y)
cor=0.0097, p=0.79
Tumor Stage 1
cor=0.059, p=0.1
Tumor Stage 2
cor=–0.06, p=0.096
Tumor Stage 3
cor=–0.082, p=0.023
Tumor Stage 4
cor=0.056, p=0.12
Node Stage 0
cor=–0.011, p=0.76
Node Stage 1
cor=–0.038, p=0.3
Node Stage 2
cor=–0.045, p=0.22
Node Stage 3
cor=–0.022, p=0.56
Metastasis Stage 1 (0=N, 1=Y)
cor=0.023, p=0.53
Path Stage 1
cor=0.063, p=0.084
Path Stage 2
cor=–0.089, p=0.015
Path Stage 3
cor=–0.022, p=0.55
Path Stage4
cor=–0.21, p=1.1e–08
ER Status (0=Neg., 1=Pos.)
cor=–0.2, p=5.6e–08
PR Status (0=Neg., 1=Pos.)
cor=–0.096, p=0.01
Her2 Status (0=Neg., 1=Pos.)
cor=–0.3, p=1.5e–17
AR Levels Exp
Eigengene

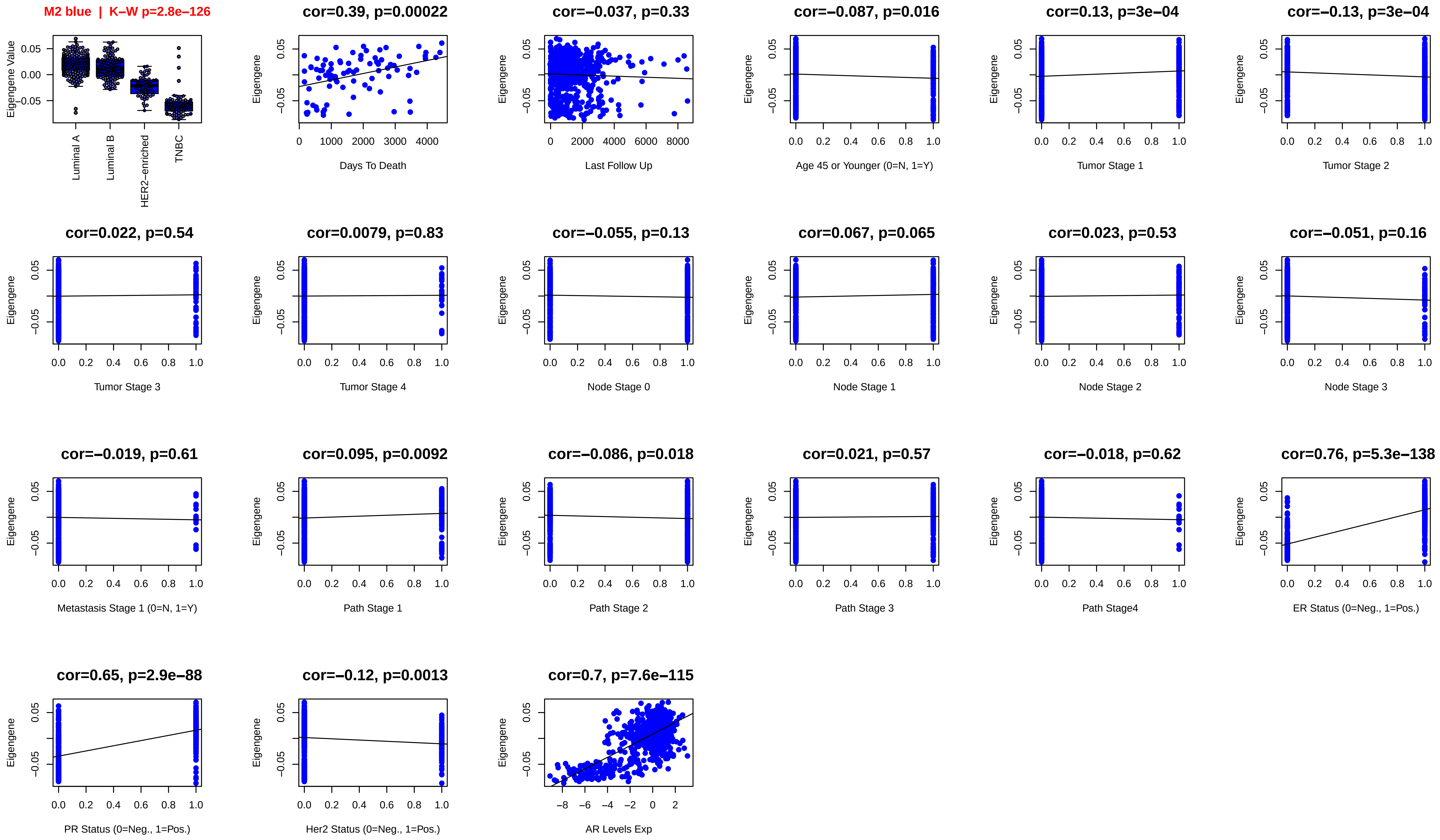

M2 blue | K–W p=2.8e–126
Eigengene Value
Luminal A
Luminal B
HER2–enriched
TNBC
cor=0.39, p=0.00022
Days To Death
cor=–0.037, p=0.33
Last Follow Up
cor=–0.087, p=0.016
Age 45 or Younger (0=N, 1=Y)
cor=0.13, p=3e–04
Tumor Stage 1
cor=–0.13, p=3e–04
Tumor Stage 2
cor=0.022, p=0.54
Tumor Stage 3
cor=0.0079, p=0.83
Tumor Stage 4
cor=–0.055, p=0.13
Node Stage 0
cor=0.067, p=0.065
Node Stage 1
cor=0.023, p=0.53
Node Stage 2
cor=–0.051, p=0.16
Node Stage 3
cor=–0.019, p=0.61
Metastasis Stage 1 (0=N, 1=Y)
cor=0.095, p=0.0092
Path Stage 1
cor=–0.086, p=0.018
Path Stage 2
cor=0.021, p=0.57
Path Stage 3
cor=–0.018, p=0.62
Path Stage4
cor=0.76, p=5.3e–138
ER Status (0=Neg., 1=Pos.)
cor=0.65, p=2.9e–88
PR Status (0=Neg., 1=Pos.)
cor=–0.12, p=0.0013
Her2 Status (0=Neg., 1=Pos.)
cor=0.7, p=7.6e–115
AR Levels Exp
Eigengene

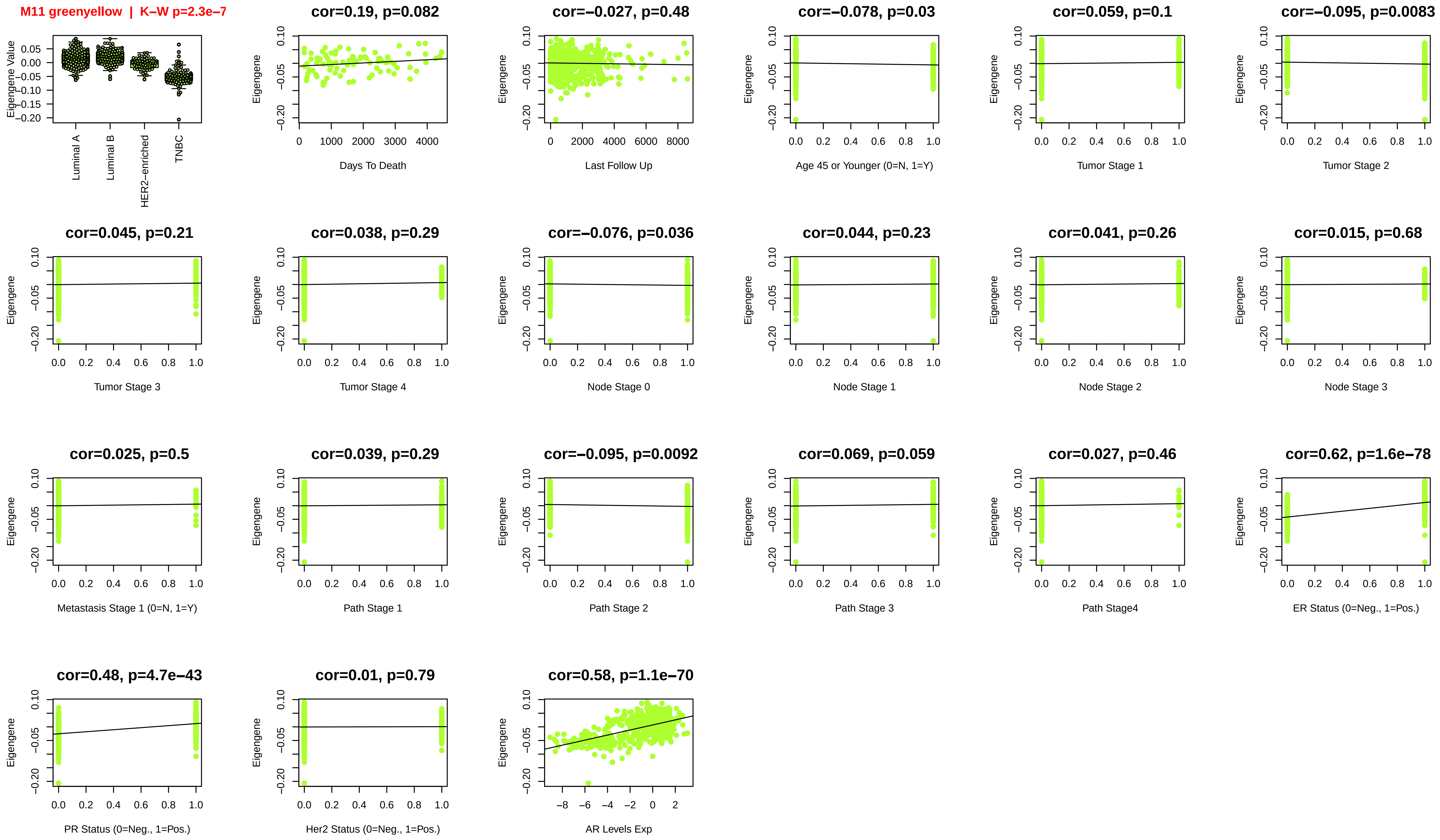
M11 greenyellow | K–W p=2.3e–7
Eigengene Value
Luminal A
Luminal B
HER2–enriched
TNBC
cor=0.19, p=0.082
Days To Death
cor=–0.027, p=0.48
Last Follow Up
cor=–0.078, p=0.03
Age 45 or Younger (0=N, 1=Y)
cor=0.059, p=0.1
Tumor Stage 1
cor=–0.095, p=0.0083
Tumor Stage 2
cor=0.045, p=0.21
Tumor Stage 3
cor=0.038, p=0.29
Tumor Stage 4
cor=–0.076, p=0.036
Node Stage 0
cor=0.044, p=0.23
Node Stage 1
cor=0.041, p=0.26
Node Stage 2
cor=0.015, p=0.68
Node Stage 3
cor=0.025, p=0.5
Metastasis Stage 1 (0=N, 1=Y)
cor=0.039, p=0.29
Path Stage 1
cor=–0.095, p=0.0092
Path Stage 2
cor=0.069, p=0.059
Path Stage 3
cor=0.027, p=0.46
Path Stage4
cor=0.62, p=1.6e–78
ER Status (0=Neg., 1=Pos.)
cor=0.48, p=4.7e–43
PR Status (0=Neg., 1=Pos.)
cor=0.01, p=0.79
Her2 Status (0=Neg., 1=Pos.)
cor=0.58, p=1.1e–70
AR Levels Exp
Eigengene

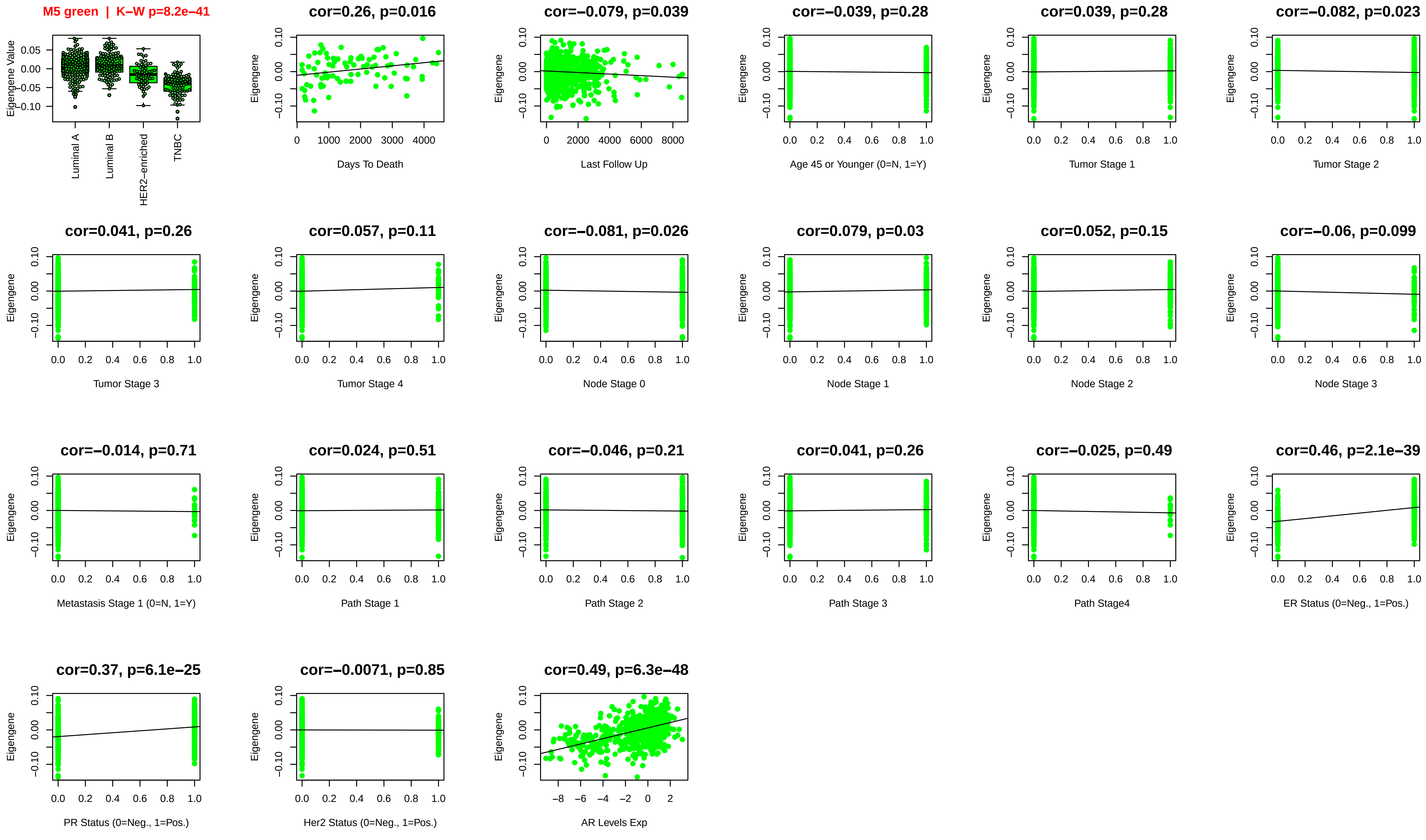
M5 green | K–W p=8.2e–41
Eigengene Value
Luminal A
Luminal B
HER2–enriched
TNBC
cor=0.26, p=0.016
Days To Death
cor=–0.079, p=0.039
Last Follow Up
cor=–0.039, p=0.28
Age 45 or Younger (0=N, 1=Y)
cor=0.039, p=0.28
Tumor Stage 1
cor=–0.082, p=0.023
Tumor Stage 2
cor=0.041, p=0.26
Tumor Stage 3
cor=0.057, p=0.11
Tumor Stage 4
cor=–0.081, p=0.026
Node Stage 0
cor=0.079, p=0.03
Node Stage 1
cor=0.052, p=0.15
Node Stage 2
cor=–0.06, p=0.099
Node Stage 3
cor=–0.014, p=0.71
Metastasis Stage 1 (0=N, 1=Y)
cor=0.024, p=0.51
Path Stage 1
cor=–0.046, p=0.21
Path Stage 2
cor=0.041, p=0.26
Path Stage 3
cor=–0.025, p=0.49
Path Stage4
cor=0.46, p=2.1e–39
ER Status (0=Neg., 1=Pos.)
cor=0.37, p=6.1e–25
PR Status (0=Neg., 1=Pos.)
cor=–0.0071, p=0.85
Her2 Status (0=Neg., 1=Pos.)
cor=0.49, p=6.3e–48
AR Levels Exp
Eigengene

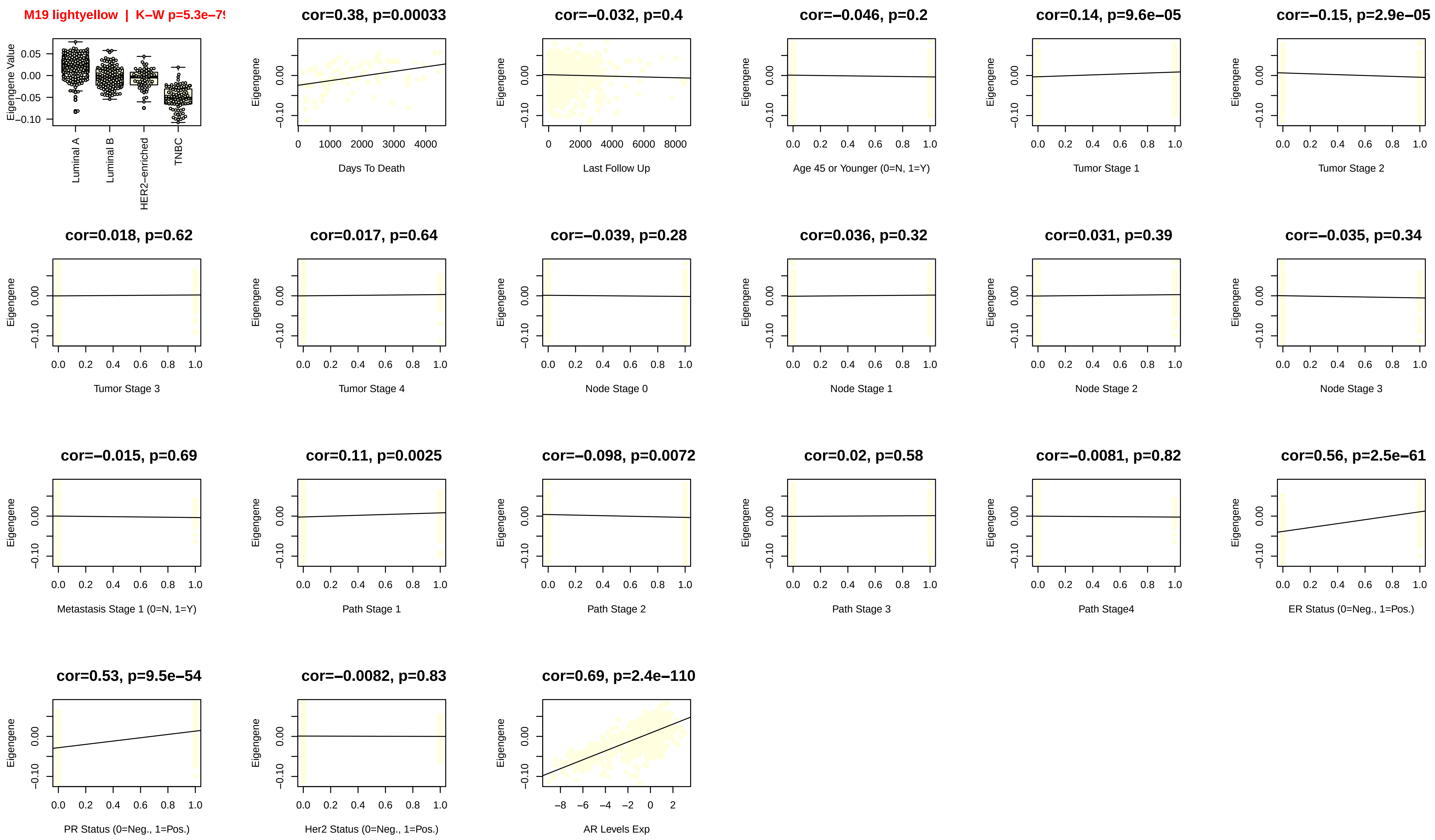

M19 lightyellow | K–W p=5.3e–7
Eigengene Value
Luminal A
Luminal B
HER2–enriched
TNBC
cor=0.38, p=0.00033
Days To Death
cor=–0.032, p=0.4
Last Follow Up
cor=–0.046, p=0.2
Age 45 or Younger (0=N, 1=Y)
cor=0.14, p=9.6e–05
Tumor Stage 1
cor=–0.15, p=2.9e–05
Tumor Stage 2
cor=0.018, p=0.62
Tumor Stage 3
cor=0.017, p=0.64
Tumor Stage 4
cor=–0.039, p=0.28
Node Stage 0
cor=0.036, p=0.32
Node Stage 1
cor=0.031, p=0.39
Node Stage 2
cor=–0.035, p=0.34
Node Stage 3
cor=–0.015, p=0.69
Metastasis Stage 1 (0=N, 1=Y)
cor=0.11, p=0.0025
Path Stage 1
cor=–0.098, p=0.0072
Path Stage 2
cor=0.02, p=0.58
Path Stage 3
cor=–0.0081, p=0.82
Path Stage4
cor=0.56, p=2.5e–61
ER Status (0=Neg., 1=Pos.)
cor=0.53, p=9.5e–54
PR Status (0=Neg., 1=Pos.)
cor=–0.0082, p=0.83
Her2 Status (0=Neg., 1=Pos.)
cor=0.69, p=2.4e–110
AR Levels Exp
Eigengene
