# Supplementary material for: A network approach reveals driver genes associated with survival of patients with triple-negative breast cancer
Source: iScience. 2021 Apr 19;24(5):102451. doi: 10.1016/j.isci.2021.102451 (PMC8111681; doi:10.1016/j.isci.2021.102451)
Supplement: Data S2. Global plots of BrCa coexpression network identify modules correlated to TNBC subtype-relevant traits with regression of age and race, Figure 2, 3, and 4 — The compilation of figures presented in Supplemental Dataset 2 is also the full print out of results from WGCNA analysis. Age and race were regressed with bootstrap nonparametric regression, and identical module membership to that found in the unregressed data was used for calculation of eigengenes. See also Figures 2 and 4. See also Tables S4 and S5. [file mmc15.zip › Supplemental Dataset2.pdf]

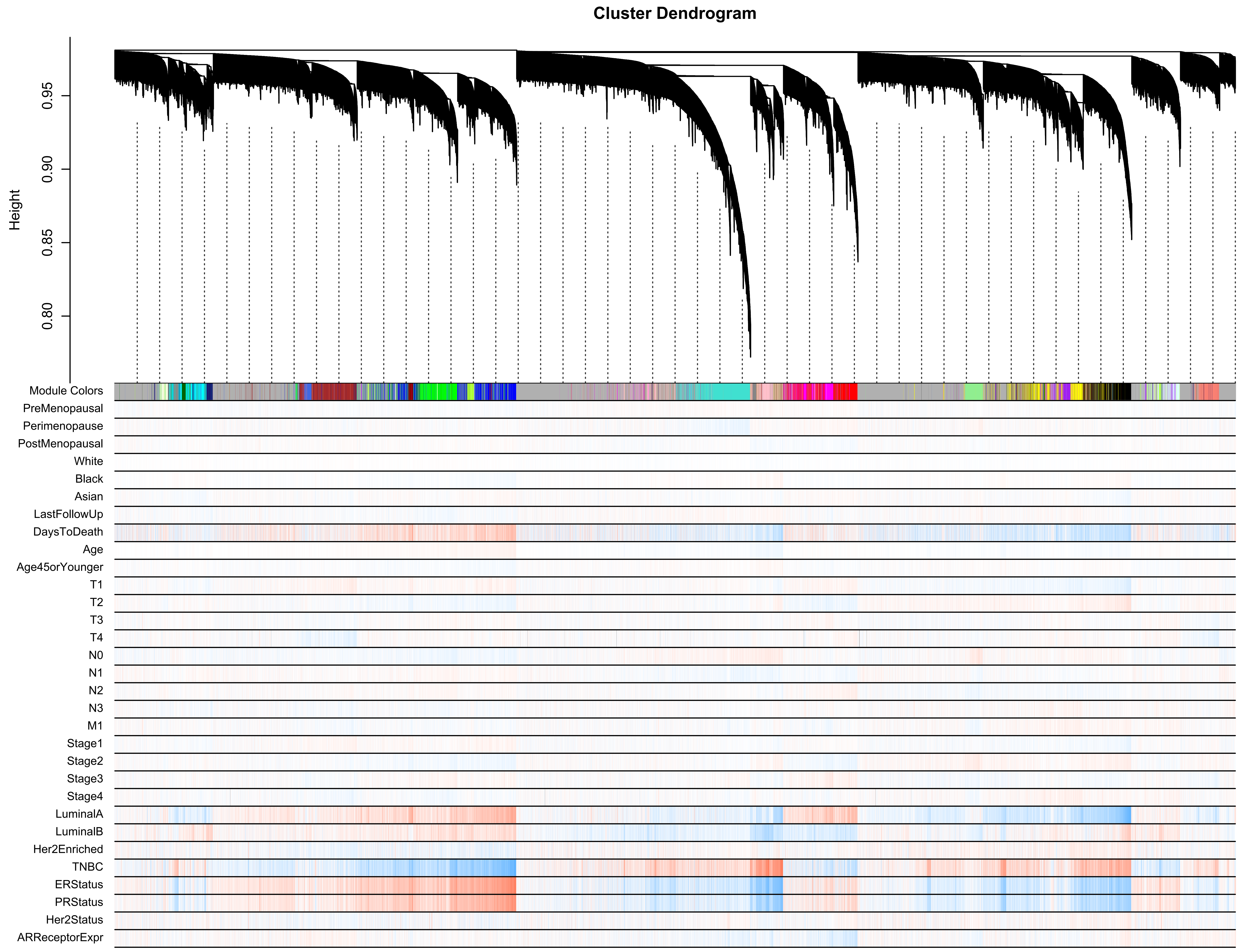

Cluster Dendrogram
Height
0.80
0.85
0.90
0.95
Module Colors
PreMenopausal
Perimenopause
PostMenopausal
White
Black
Asian
LastFollowUp
DaysToDeath
Age
Age45orYounger
T1
T2
T3
T4
N0
N1
N2
N3
M1
Stage1
Stage2
Stage3
Stage4
LuminalA
LuminalB
Her2Enriched
TNBC
ERStatus
PRStatus
Her2Status
ARReceptorExpr

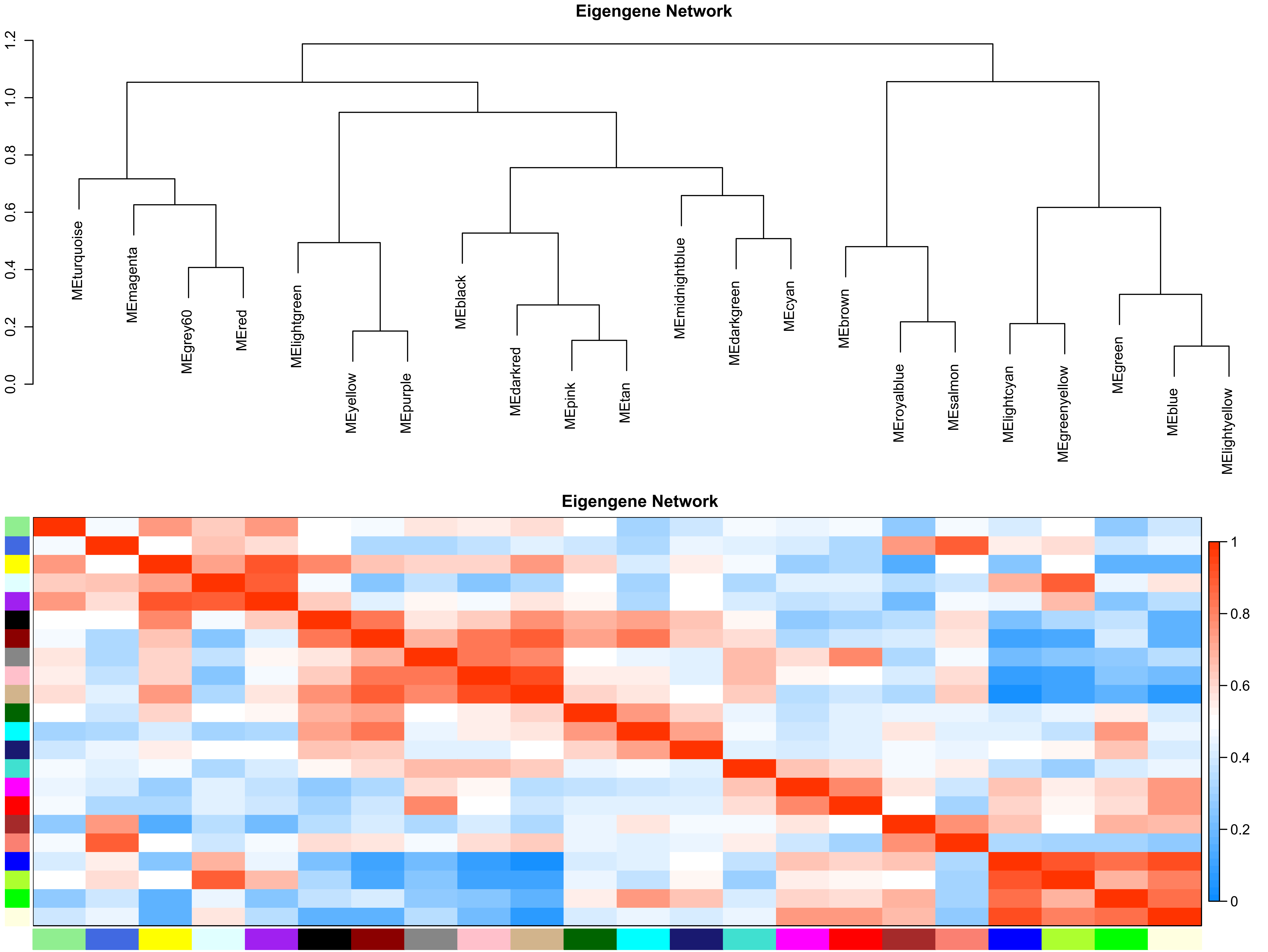
Eigengene Network
0.0
0.2
0.4
0.6
0.8
1.0
1.2
MEturquoise
MEmagenta
MEgrey60
MEred
MElightgreen
MEyellow
MEpurple
MEblack
MEdarkred
MEpink
MEtan
MEmidnightblue
MEdarkgreen
MEcyan
MEbrown
MEroyalblue
MEsalmon
MElightcyan
MEgreenyellow
MEgreen
MEblue
MElightyellow
Eigengene Network
1
0.8
0.6
0.4
0.2
0

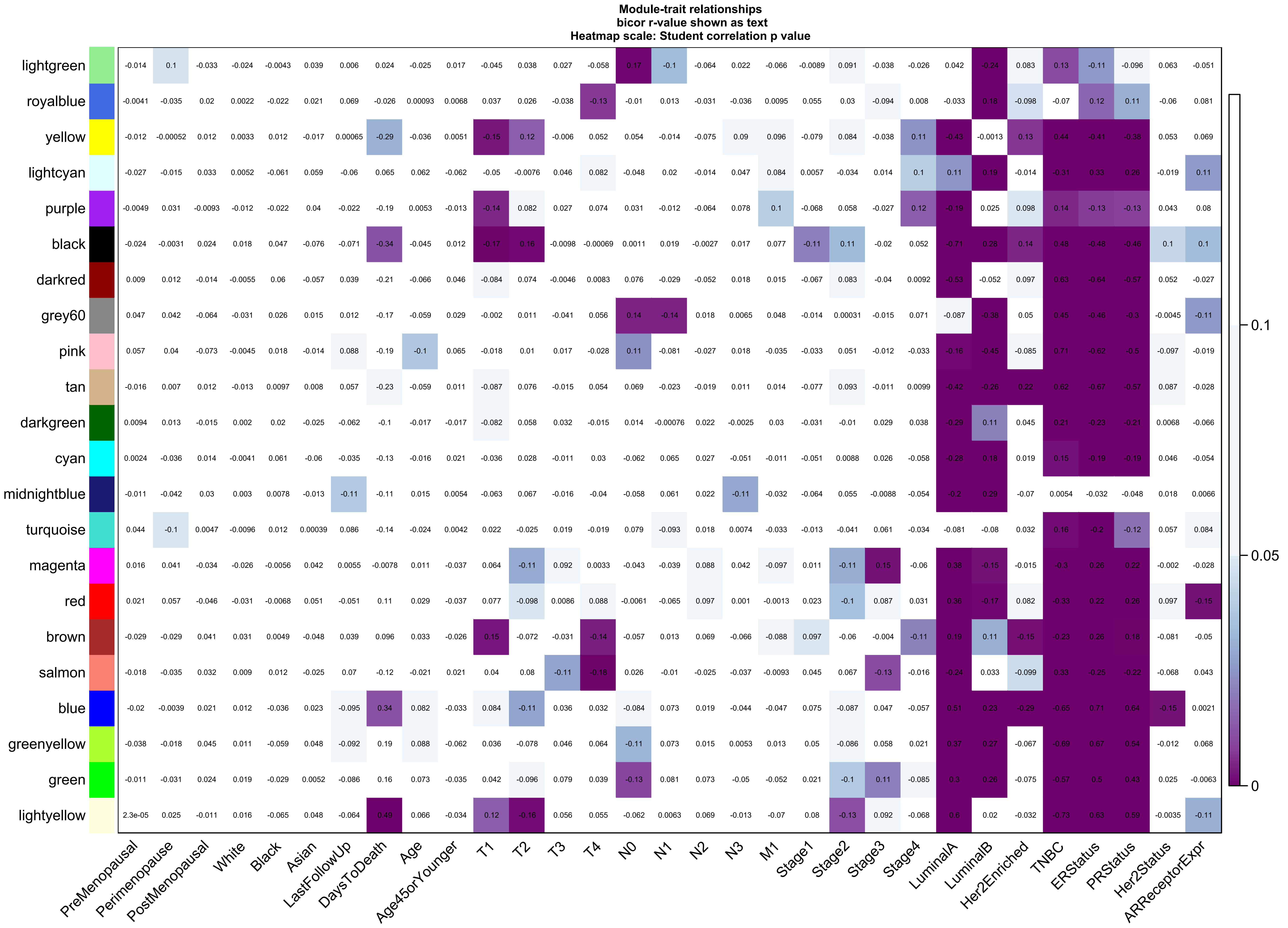
Module-trait relationships
bicor r-value shown as text
Heatmap scale: Student correlation p value
lightgreen -0.014 0.1 -0.033 -0.024 -0.0043 0.039 0.006 0.024 -0.025 0.017 -0.045 0.038 0.027 -0.058 0.17 -0.1 -0.064 0.022 -0.066 -0.0089 0.091 -0.038 -0.026 0.042 -0.24 0.083 0.13 -0.11 -0.096 0.063 -0.051
royalblue -0.0041 -0.035 0.02 0.0022 -0.022 0.021 0.069 -0.026 0.00093 0.0068 0.037 0.026 -0.038 -0.13 -0.01 0.013 -0.031 -0.036 0.0095 0.055 0.03 -0.094 0.008 -0.033 0.18 -0.098 -0.07 0.12 0.11 -0.06 0.081
yellow -0.012 -0.00052 0.012 0.0033 0.012 -0.017 0.00065 -0.29 -0.036 0.0051 -0.15 0.12 -0.006 0.052 0.054 -0.014 -0.075 0.09 0.096 -0.079 0.084 -0.038 0.11 -0.43 -0.0013 0.13 0.44 -0.41 -0.38 0.053 0.069
lightcyan -0.027 -0.015 0.033 0.0052 -0.061 0.059 -0.06 0.065 0.062 -0.062 -0.05 -0.0076 0.046 0.082 -0.048 0.02 -0.014 0.047 0.084 0.0057 -0.034 0.014 0.1 0.11 0.19 -0.014 -0.31 0.33 0.26 -0.019 0.11
purple -0.0049 0.031 -0.0093 -0.012 -0.022 0.04 -0.022 -0.19 0.0053 -0.013 -0.14 0.082 0.027 0.074 0.031 -0.012 -0.064 0.078 0.1 -0.068 0.058 -0.027 0.12 -0.19 0.025 0.098 0.14 -0.13 -0.13 0.043 0.08
black -0.024 -0.0031 0.024 0.018 0.047 -0.076 -0.071 -0.34 -0.045 0.012 -0.17 0.16 -0.0098 -0.00069 0.0011 0.019 -0.0027 0.017 0.077 -0.11 0.11 -0.02 0.052 -0.71 0.28 0.14 0.48 -0.48 -0.46 0.1 0.1
darkred 0.009 0.012 -0.014 -0.0055 0.06 -0.057 0.039 -0.21 -0.066 0.046 -0.084 0.074 -0.0046 0.0083 0.076 -0.029 -0.052 0.018 0.015 -0.067 0.083 -0.04 0.0092 -0.53 -0.052 0.097 0.63 -0.64 -0.57 0.052 -0.027
grey60 0.047 0.042 -0.064 -0.031 0.026 0.015 0.012 -0.17 -0.059 0.029 -0.002 0.011 -0.041 0.056 0.14 -0.14 0.018 0.0065 0.048 -0.014 0.00031 -0.015 0.071 -0.087 -0.38 0.05 0.45 -0.46 -0.3 -0.0045 -0.11
pink 0.057 0.04 -0.073 -0.0045 0.018 -0.014 0.088 -0.19 -0.1 0.065 -0.018 0.01 0.017 -0.028 0.11 -0.081 -0.027 0.018 -0.035 -0.033 0.051 -0.012 -0.033 -0.16 -0.45 -0.085 0.71 -0.62 -0.5 -0.097 -0.019
tan -0.016 0.007 0.012 -0.013 0.0097 0.008 0.057 -0.23 -0.059 0.011 -0.087 0.076 -0.015 0.054 0.069 -0.023 -0.019 0.011 0.014 -0.077 0.093 -0.011 0.0099 -0.42 -0.26 0.22 0.62 -0.67 -0.57 0.087 -0.028
darkgreen 0.0094 0.013 -0.015 0.002 0.02 -0.025 -0.062 -0.1 -0.017 -0.017 -0.082 0.058 0.032 -0.015 0.014 -0.00076 0.022 -0.0025 0.03 -0.031 -0.01 0.029 0.038 -0.29 0.11 0.045 0.21 -0.23 -0.21 0.0068 -0.066
cyan 0.0024 -0.036 0.014 -0.0041 0.061 -0.06 -0.035 -0.13 -0.016 0.021 -0.036 0.028 -0.011 0.03 -0.062 0.065 0.027 -0.051 -0.011 -0.051 0.0088 0.026 -0.058 -0.28 0.18 0.019 0.15 -0.19 -0.19 0.046 -0.054
midnightblue -0.011 -0.042 0.03 0.003 0.0078 -0.013 -0.11 -0.11 0.015 0.0054 -0.063 0.067 -0.016 -0.04 -0.058 0.061 0.022 -0.11 -0.032 -0.064 0.055 -0.0088 -0.054 -0.2 0.29 -0.07 0.0054 -0.032 -0.048 0.018 0.0066
turquoise 0.044 -0.1 0.0047 -0.0096 0.012 0.00039 0.086 -0.14 -0.024 0.0042 0.022 -0.025 0.019 -0.019 0.079 -0.093 0.018 0.0074 -0.033 -0.013 -0.041 0.061 -0.034 -0.081 -0.08 0.032 0.16 -0.2 -0.12 0.057 0.084
magenta 0.016 0.041 -0.034 -0.026 -0.0056 0.042 0.0055 -0.0078 0.011 -0.037 0.064 -0.11 0.092 0.0033 -0.043 -0.039 0.088 0.042 -0.097 0.011 -0.11 0.15 -0.06 0.38 -0.15 -0.015 -0.3 0.26 0.22 -0.002 -0.028
red 0.021 0.057 -0.046 -0.031 -0.0068 0.051 -0.051 0.11 0.029 -0.037 0.077 -0.098 0.0086 0.088 -0.0061 -0.065 0.097 0.001 -0.0013 0.023 -0.1 0.087 0.031 0.36 -0.17 0.082 -0.33 0.22 0.26 0.097 -0.15
brown -0.029 -0.029 0.041 0.031 0.0049 -0.048 0.039 0.096 0.033 -0.026 0.15 -0.072 -0.031 -0.14 -0.057 0.013 0.069 -0.066 -0.088 0.097 -0.06 -0.004 -0.11 0.19 0.11 -0.15 -0.23 0.26 0.18 -0.081 -0.05
salmon -0.018 -0.035 0.032 0.009 0.012 -0.025 0.07 -0.12 -0.021 0.021 0.04 0.08 -0.11 -0.18 0.026 -0.01 -0.025 -0.037 -0.0093 0.045 0.067 -0.13 -0.016 -0.24 0.033 -0.099 0.33 -0.25 -0.22 -0.068 0.043
blue -0.02 -0.0039 0.021 0.012 -0.036 0.023 -0.095 0.34 0.082 -0.033 0.084 -0.11 0.036 0.032 -0.084 0.073 0.019 -0.044 -0.047 0.075 -0.087 0.047 -0.057 0.51 0.23 -0.29 -0.65 0.71 0.64 -0.15 0.0021
greenyellow -0.038 -0.018 0.045 0.011 -0.059 0.048 -0.092 0.19 0.088 -0.062 0.036 -0.078 0.046 0.064 -0.11 0.073 0.015 0.0053 0.013 0.05 -0.086 0.058 0.021 0.37 0.27 -0.067 -0.69 0.67 0.54 -0.012 0.068
green -0.011 -0.031 0.024 0.019 -0.029 0.0052 -0.086 0.16 0.073 -0.035 0.042 -0.096 0.079 0.039 -0.13 0.081 0.073 -0.05 -0.052 0.021 -0.1 0.11 -0.085 0.3 0.26 -0.075 -0.57 0.5 0.43 0.025 -0.0063
lightyellow 2.3e-05 0.025 -0.011 0.016 -0.065 0.048 -0.064 0.49 0.066 -0.034 0.12 -0.16 0.056 0.055 -0.062 0.0063 0.069 -0.013 -0.07 0.08 -0.13 0.092 -0.068 0.6 0.02 -0.032 -0.73 0.63 0.59 -0.0035 -0.11
PreMenopausal Perimenopause PostMenopausal White Black Asian LastFollowUp DaysToDeath Age Age45orYounger T1 T2 T3 T4 N0 N1 N2 N3 M1 Stage1 Stage2 Stage3 Stage4 LuminalA LuminalB Her2Enriched TNBC ERStatus PRStatus Her2Status ARReceptorExpr
0.1
0.05
0

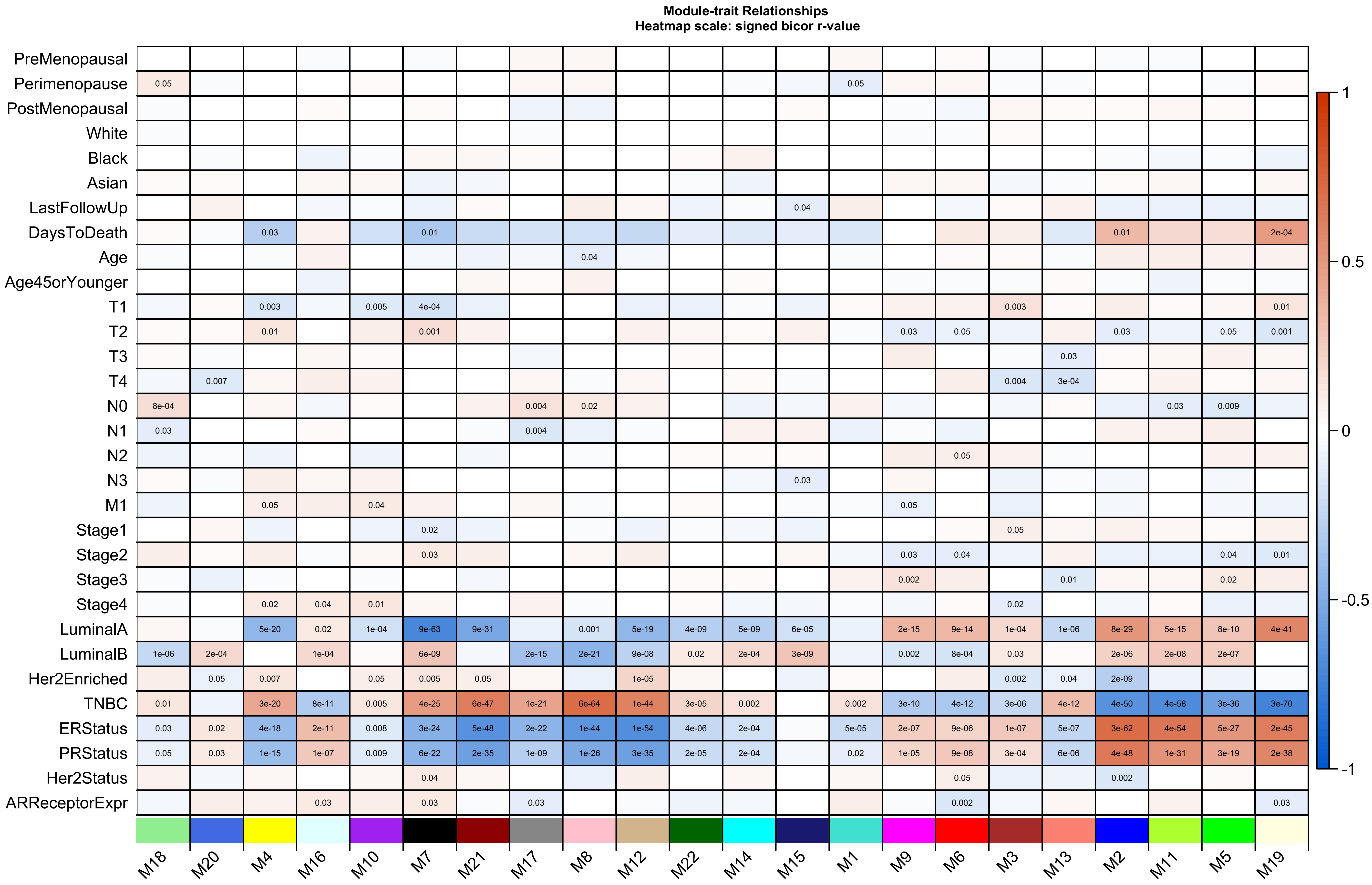
Module-trait Relationships
Heatmap scale: signed bicor r-value
PreMenopausal
Perimenopause
PostMenopausal
White
Black
Asian
LastFollowUp
DaysToDeath
Age
Age45orYounger
T1
T2
T3
T4
N0
N1
N2
N3
M1
Stage1
Stage2
Stage3
Stage4
LuminalA
LuminalB
Her2Enriched
TNBC
ERStatus
PRStatus
Her2Status
ARReceptorExpr
M18
M20
M4
M16
M10
M7
M21
M17
M8
M12
M22
M14
M15
M1
M9
M6
M3
M13
M2
M11
M5
M19
1
0.5
0
-0.5
-1

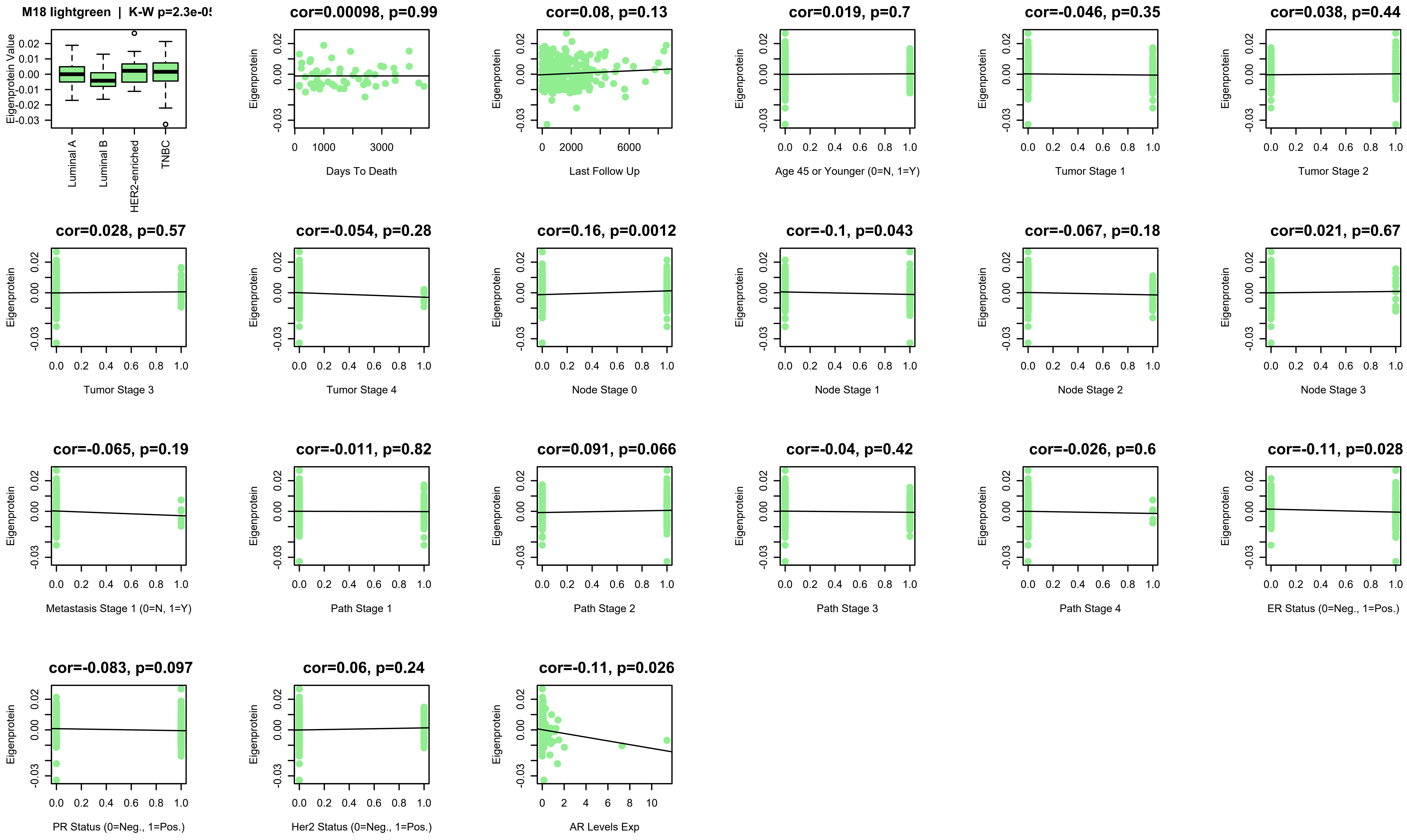
M18 lightgreen | K-W p=2.3e-05
Eigenprotein Value
Luminal A
Luminal B
HER2-enriched
TNBC
cor=0.00098, p=0.99
Days To Death
cor=0.08, p=0.13
Last Follow Up
cor=0.019, p=0.7
Age 45 or Younger (0=N, 1=Y)
cor=-0.046, p=0.35
Tumor Stage 1
cor=0.038, p=0.44
Tumor Stage 2
cor=0.028, p=0.57
Tumor Stage 3
cor=-0.054, p=0.28
Tumor Stage 4
cor=0.16, p=0.0012
Node Stage 0
cor=-0.1, p=0.043
Node Stage 1
cor=-0.067, p=0.18
Node Stage 2
cor=0.021, p=0.67
Node Stage 3
cor=-0.065, p=0.19
Metastasis Stage 1 (0=N, 1=Y)
cor=-0.011, p=0.82
Path Stage 1
cor=0.091, p=0.066
Path Stage 2
cor=-0.04, p=0.42
Path Stage 3
cor=-0.026, p=0.6
Path Stage 4
cor=-0.11, p=0.028
ER Status (0=Neg., 1=Pos.)
cor=-0.083, p=0.097
PR Status (0=Neg., 1=Pos.)
cor=0.06, p=0.24
Her2 Status (0=Neg., 1=Pos.)
cor=-0.11, p=0.026
AR Levels Exp
Eigenprotein

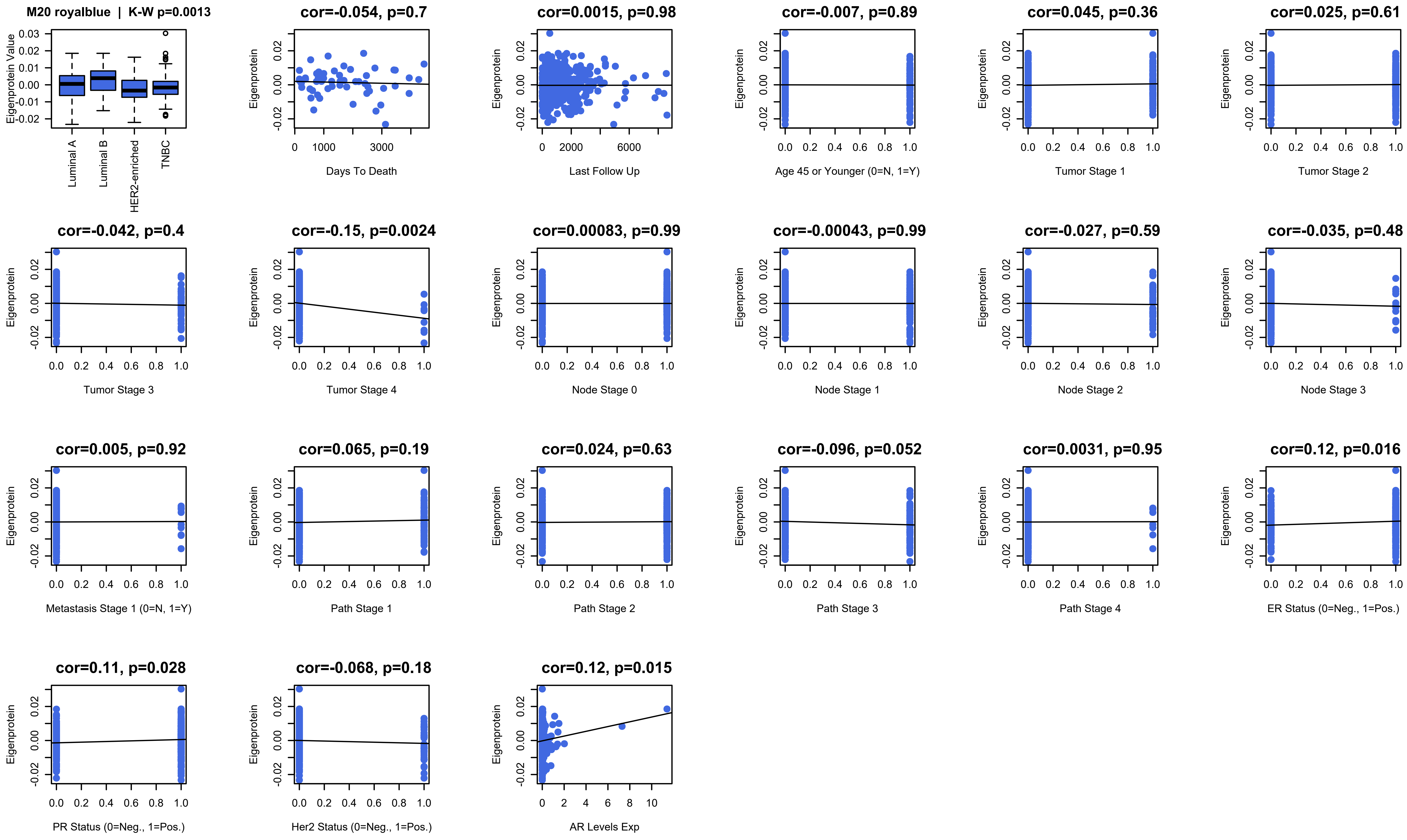
M20 royalblue | K-W p=0.0013
Eigenprotein Value
Luminal A
Luminal B
HER2-enriched
TNBC
cor=-0.054, p=0.7
Days To Death
cor=0.0015, p=0.98
Last Follow Up
cor=-0.007, p=0.89
Age 45 or Younger (0=N, 1=Y)
cor=0.045, p=0.36
Tumor Stage 1
cor=0.025, p=0.61
Tumor Stage 2
cor=-0.042, p=0.4
Tumor Stage 3
cor=-0.15, p=0.0024
Tumor Stage 4
cor=0.00083, p=0.99
Node Stage 0
cor=-0.00043, p=0.99
Node Stage 1
cor=-0.027, p=0.59
Node Stage 2
cor=-0.035, p=0.48
Node Stage 3
cor=0.005, p=0.92
Metastasis Stage 1 (0=N, 1=Y)
cor=0.065, p=0.19
Path Stage 1
cor=0.024, p=0.63
Path Stage 2
cor=-0.096, p=0.052
Path Stage 3
cor=0.0031, p=0.95
Path Stage 4
cor=0.12, p=0.016
ER Status (0=Neg., 1=Pos.)
cor=0.11, p=0.028
PR Status (0=Neg., 1=Pos.)
cor=-0.068, p=0.18
Her2 Status (0=Neg., 1=Pos.)
cor=0.12, p=0.015
AR Levels Exp
Eigenprotein

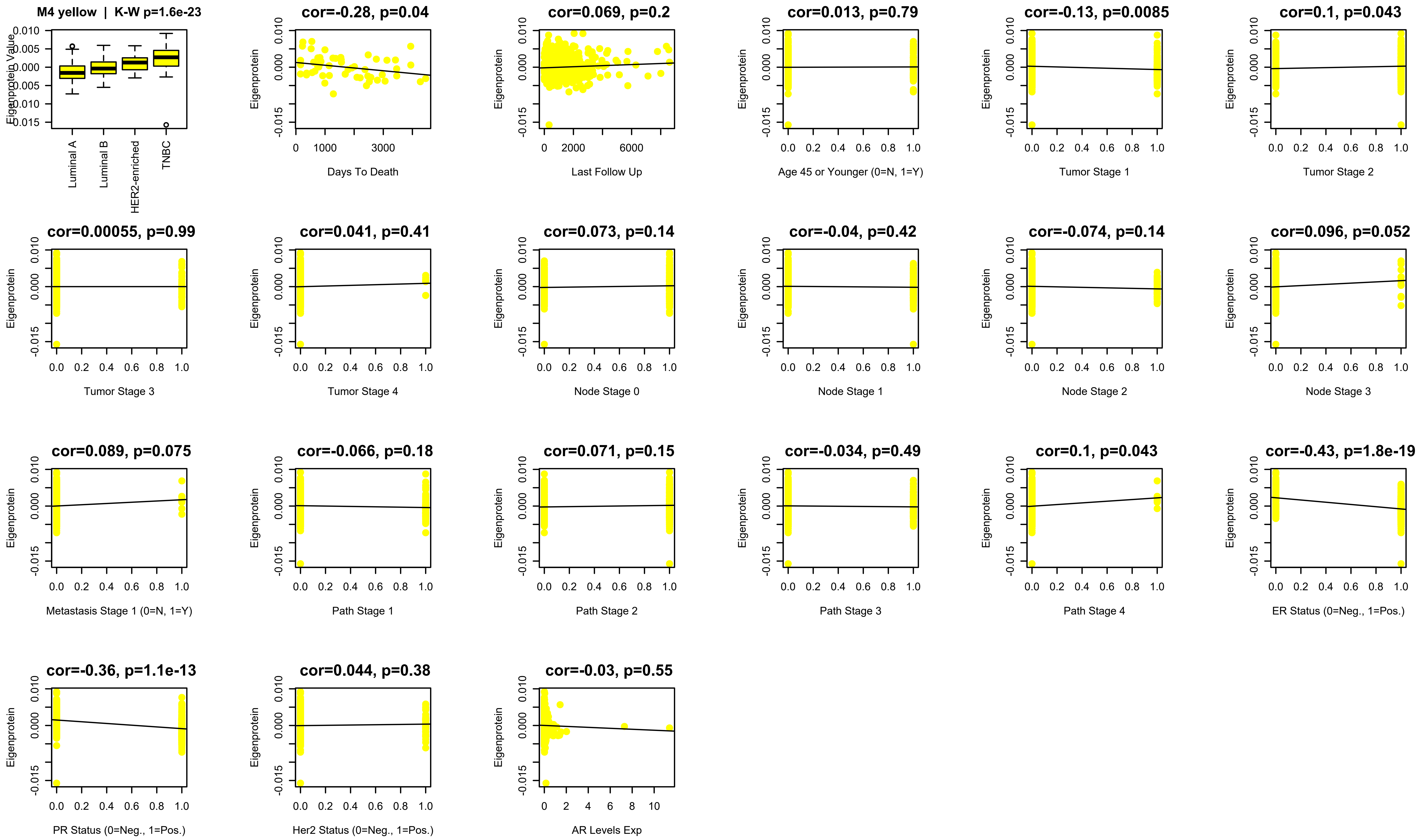

M4 yellow | K-W p=1.6e-23
Eigenprotein Value
Luminal A
Luminal B
HER2-enriched
TNBC
cor=-0.28, p=0.04
Days To Death
cor=0.069, p=0.2
Last Follow Up
cor=0.013, p=0.79
Age 45 or Younger (0=N, 1=Y)
cor=-0.13, p=0.0085
Tumor Stage 1
cor=0.1, p=0.043
Tumor Stage 2
cor=0.00055, p=0.99
Tumor Stage 3
cor=0.041, p=0.41
Tumor Stage 4
cor=0.073, p=0.14
Node Stage 0
cor=-0.04, p=0.42
Node Stage 1
cor=-0.074, p=0.14
Node Stage 2
cor=0.096, p=0.052
Node Stage 3
cor=0.089, p=0.075
Metastasis Stage 1 (0=N, 1=Y)
cor=-0.066, p=0.18
Path Stage 1
cor=0.071, p=0.15
Path Stage 2
cor=-0.034, p=0.49
Path Stage 3
cor=0.1, p=0.043
Path Stage 4
cor=-0.43, p=1.8e-19
ER Status (0=Neg., 1=Pos.)
cor=-0.36, p=1.1e-13
PR Status (0=Neg., 1=Pos.)
cor=0.044, p=0.38
Her2 Status (0=Neg., 1=Pos.)
cor=-0.03, p=0.55
AR Levels Exp
Eigenprotein

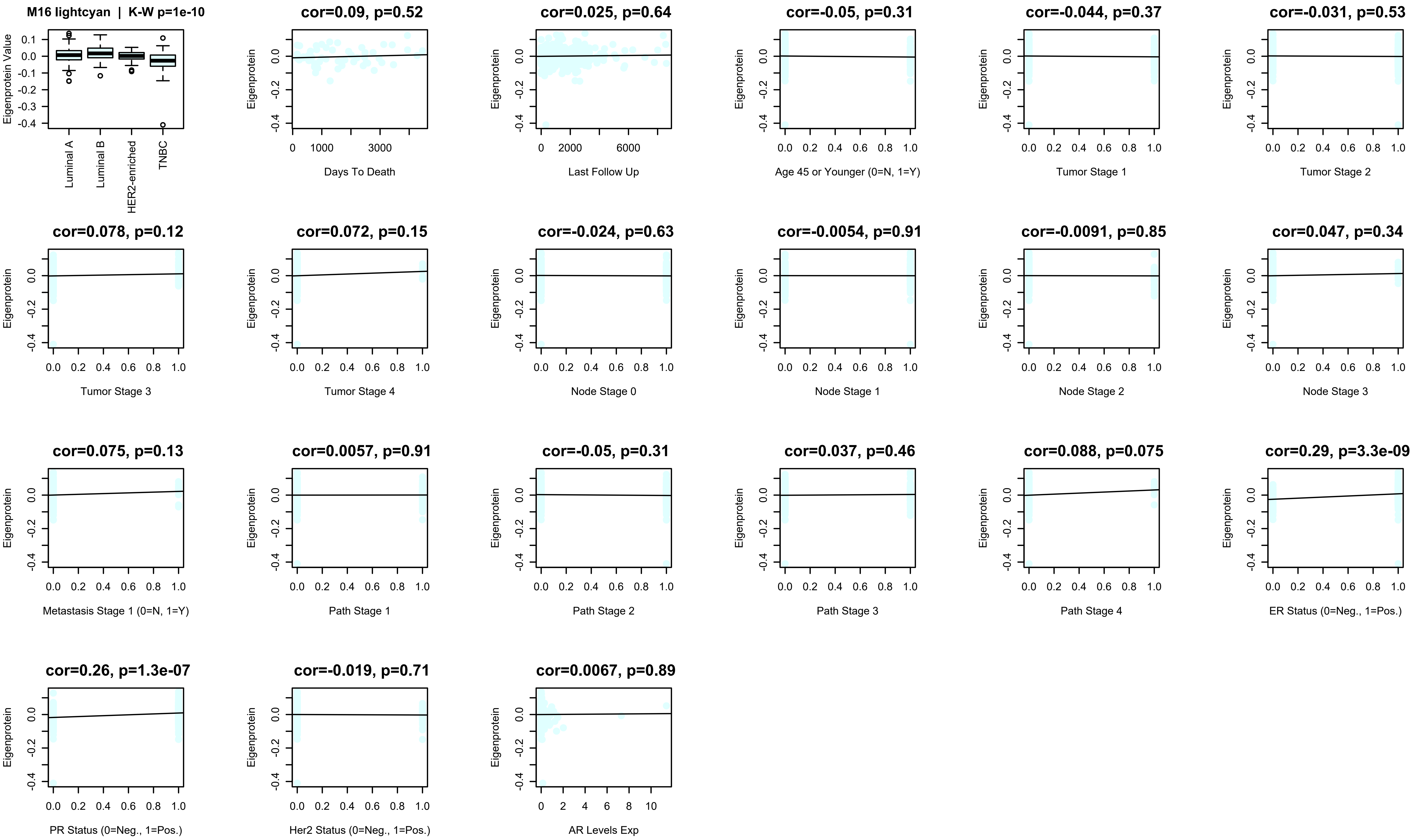
M16 lightcyan | K-W p=1e-10
Eigenprotein Value
Luminal A
Luminal B
HER2-enriched
TNBC
cor=0.09, p=0.52
Days To Death
cor=0.025, p=0.64
Last Follow Up
cor=-0.05, p=0.31
Age 45 or Younger (0=N, 1=Y)
cor=-0.044, p=0.37
Tumor Stage 1
cor=-0.031, p=0.53
Tumor Stage 2
cor=0.078, p=0.12
Tumor Stage 3
cor=0.072, p=0.15
Tumor Stage 4
cor=-0.024, p=0.63
Node Stage 0
cor=-0.0054, p=0.91
Node Stage 1
cor=-0.0091, p=0.85
Node Stage 2
cor=0.047, p=0.34
Node Stage 3
cor=0.075, p=0.13
Metastasis Stage 1 (0=N, 1=Y)
cor=0.0057, p=0.91
Path Stage 1
cor=-0.05, p=0.31
Path Stage 2
cor=0.037, p=0.46
Path Stage 3
cor=0.088, p=0.075
Path Stage 4
cor=0.29, p=3.3e-09
ER Status (0=Neg., 1=Pos.)
cor=0.26, p=1.3e-07
PR Status (0=Neg., 1=Pos.)
cor=-0.019, p=0.71
Her2 Status (0=Neg., 1=Pos.)
cor=0.0067, p=0.89
AR Levels Exp
Eigenprotein

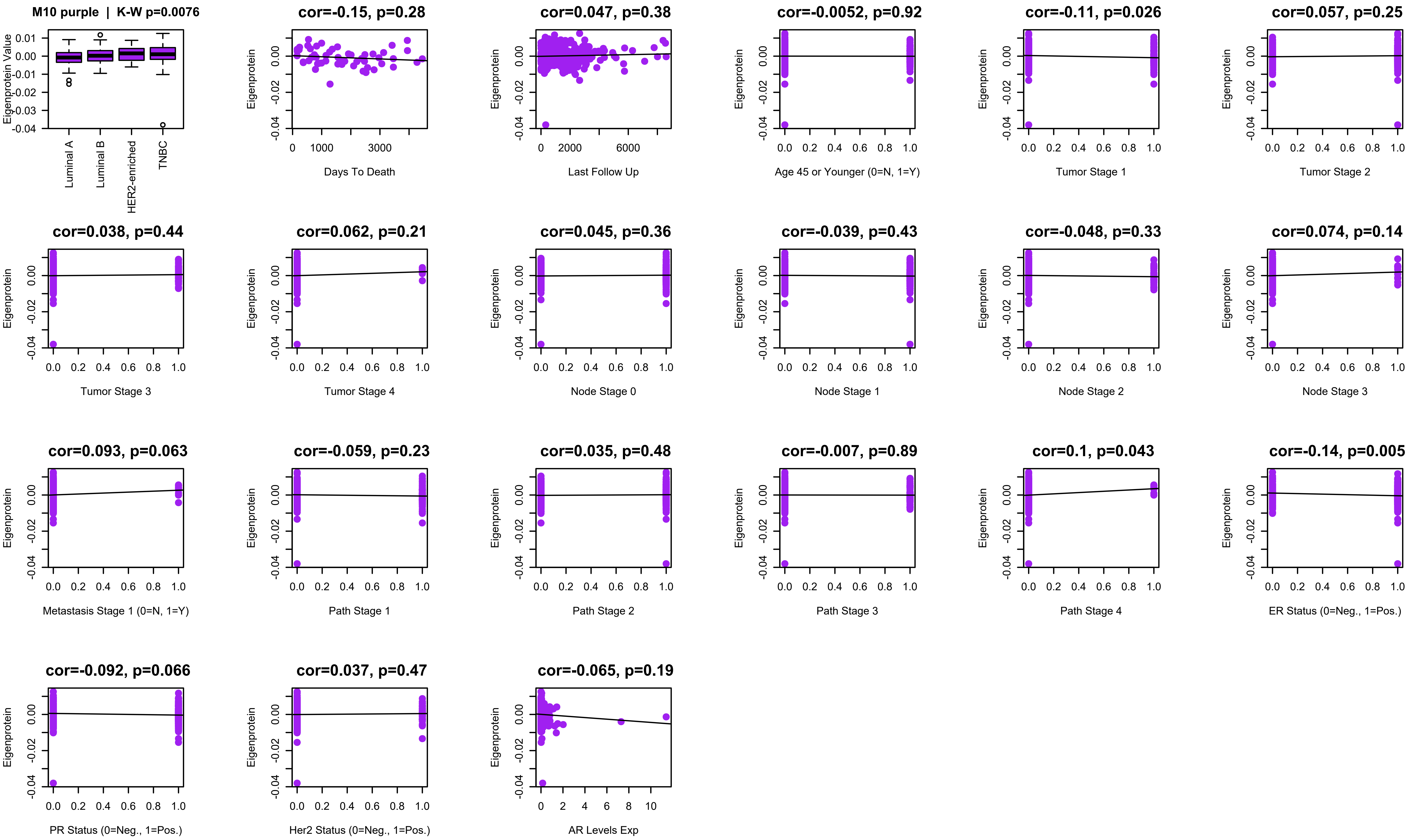
M10 purple | K-W p=0.0076
Eigenprotein Value
Luminal A
Luminal B
HER2-enriched
TNBC
cor=-0.15, p=0.28
Days To Death
cor=0.047, p=0.38
Last Follow Up
cor=-0.0052, p=0.92
Age 45 or Younger (0=N, 1=Y)
cor=-0.11, p=0.026
Tumor Stage 1
cor=0.057, p=0.25
Tumor Stage 2
cor=0.038, p=0.44
Tumor Stage 3
cor=0.062, p=0.21
Tumor Stage 4
cor=0.045, p=0.36
Node Stage 0
cor=-0.039, p=0.43
Node Stage 1
cor=-0.048, p=0.33
Node Stage 2
cor=0.074, p=0.14
Node Stage 3
cor=0.093, p=0.063
Metastasis Stage 1 (0=N, 1=Y)
cor=-0.059, p=0.23
Path Stage 1
cor=0.035, p=0.48
Path Stage 2
cor=-0.007, p=0.89
Path Stage 3
cor=0.1, p=0.043
Path Stage 4
cor=-0.14, p=0.005
ER Status (0=Neg., 1=Pos.)
cor=-0.092, p=0.066
PR Status (0=Neg., 1=Pos.)
cor=0.037, p=0.47
Her2 Status (0=Neg., 1=Pos.)
cor=-0.065, p=0.19
AR Levels Exp
Eigenprotein

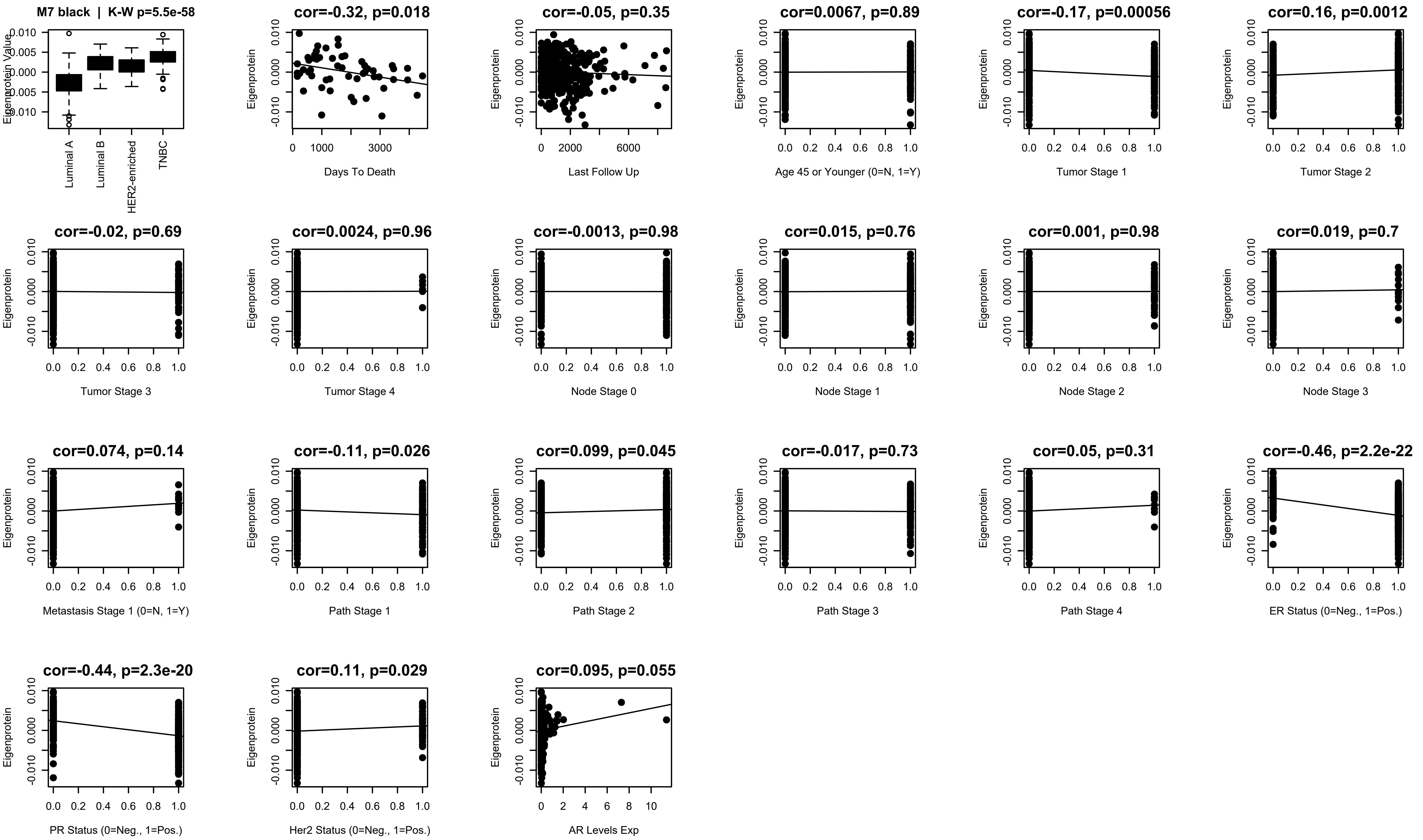
M7 black | K-W p=5.5e-58
Eigenprotein Value
Luminal A
Luminal B
HER2-enriched
TNBC
cor=-0.32, p=0.018
Days To Death
cor=-0.05, p=0.35
Last Follow Up
cor=0.0067, p=0.89
Age 45 or Younger (0=N, 1=Y)
cor=-0.17, p=0.00056
Tumor Stage 1
cor=0.16, p=0.0012
Tumor Stage 2
cor=-0.02, p=0.69
Tumor Stage 3
cor=0.0024, p=0.96
Tumor Stage 4
cor=-0.0013, p=0.98
Node Stage 0
cor=0.015, p=0.76
Node Stage 1
cor=0.001, p=0.98
Node Stage 2
cor=0.019, p=0.7
Node Stage 3
cor=0.074, p=0.14
Metastasis Stage 1 (0=N, 1=Y)
cor=-0.11, p=0.026
Path Stage 1
cor=0.099, p=0.045
Path Stage 2
cor=-0.017, p=0.73
Path Stage 3
cor=0.05, p=0.31
Path Stage 4
cor=-0.46, p=2.2e-22
ER Status (0=Neg., 1=Pos.)
cor=-0.44, p=2.3e-20
PR Status (0=Neg., 1=Pos.)
cor=0.11, p=0.029
Her2 Status (0=Neg., 1=Pos.)
cor=0.095, p=0.055
AR Levels Exp
Eigenprotein

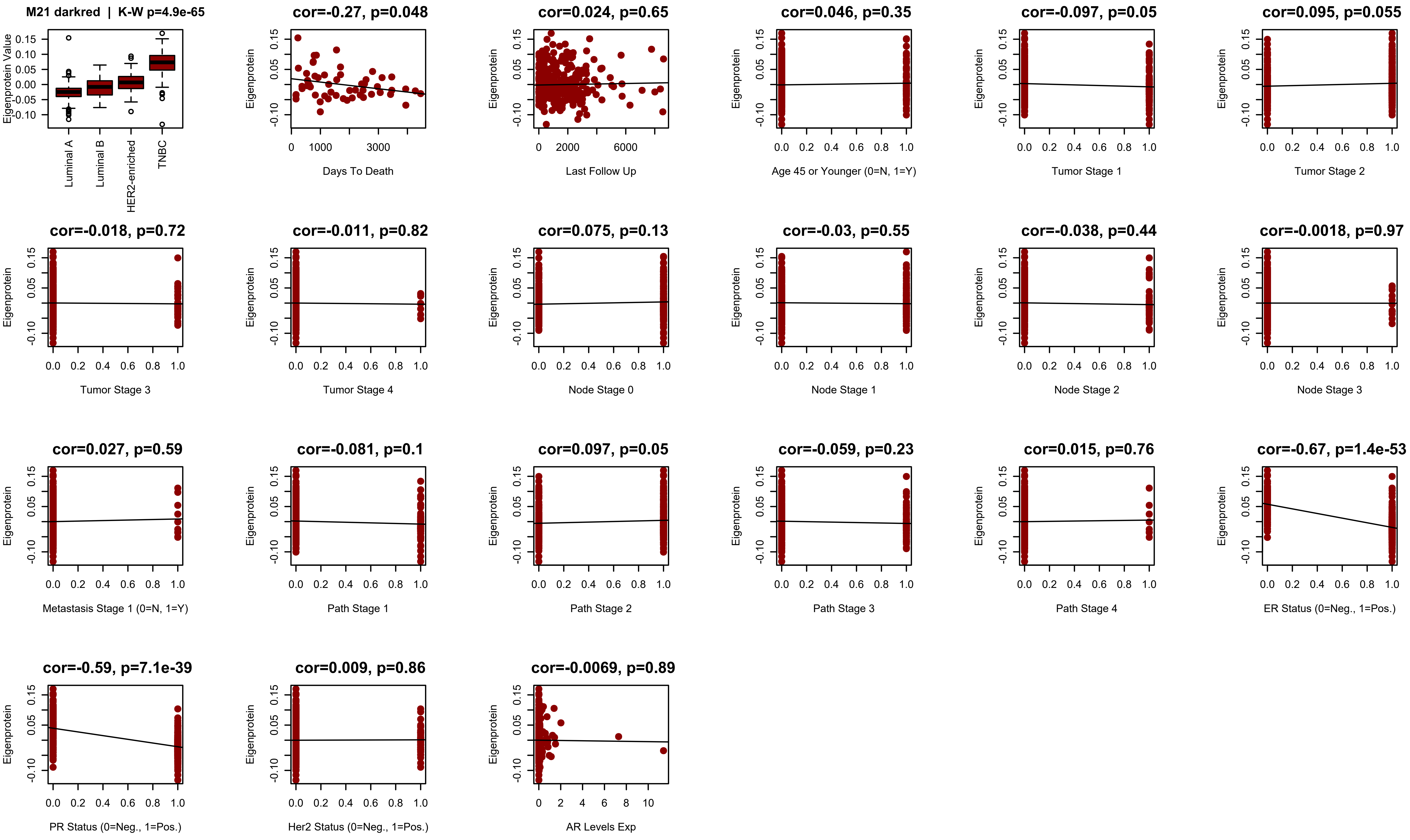
M21 darkred | K-W p=4.9e-65
Eigenprotein Value
Luminal A
Luminal B
HER2-enriched
TNBC
cor=-0.27, p=0.048
Days To Death
cor=0.024, p=0.65
Last Follow Up
cor=0.046, p=0.35
Age 45 or Younger (0=N, 1=Y)
cor=-0.097, p=0.05
Tumor Stage 1
cor=0.095, p=0.055
Tumor Stage 2
cor=-0.018, p=0.72
Tumor Stage 3
cor=-0.011, p=0.82
Tumor Stage 4
cor=0.075, p=0.13
Node Stage 0
cor=-0.03, p=0.55
Node Stage 1
cor=-0.038, p=0.44
Node Stage 2
cor=-0.0018, p=0.97
Node Stage 3
cor=0.027, p=0.59
Metastasis Stage 1 (0=N, 1=Y)
cor=-0.081, p=0.1
Path Stage 1
cor=0.097, p=0.05
Path Stage 2
cor=-0.059, p=0.23
Path Stage 3
cor=0.015, p=0.76
Path Stage 4
cor=-0.67, p=1.4e-53
ER Status (0=Neg., 1=Pos.)
cor=-0.59, p=7.1e-39
PR Status (0=Neg., 1=Pos.)
cor=0.009, p=0.86
Her2 Status (0=Neg., 1=Pos.)
cor=-0.0069, p=0.89
AR Levels Exp
Eigenprotein

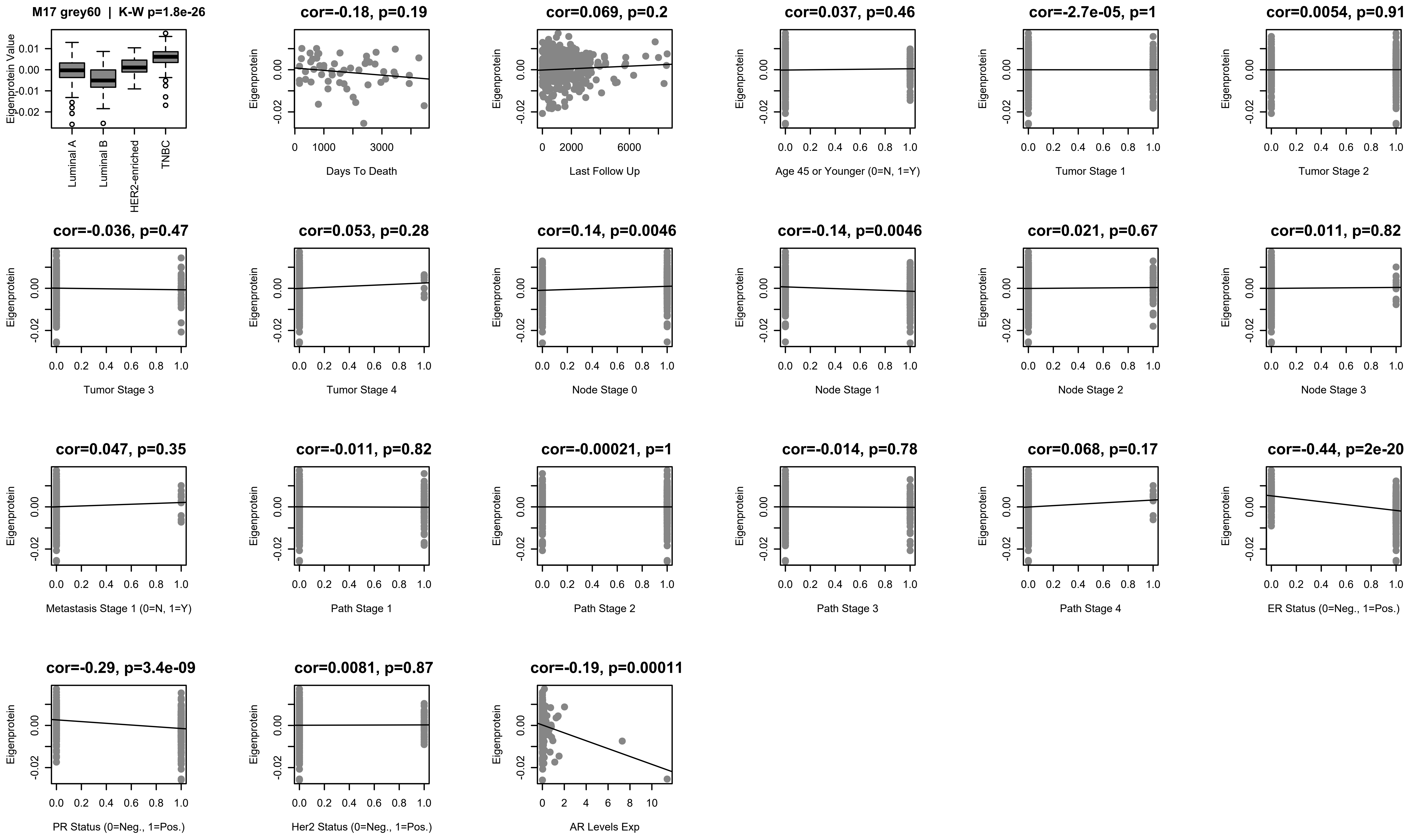
M17 grey60 | K-W p=1.8e-26
Eigenprotein Value
Luminal A
Luminal B
HER2-enriched
TNBC
cor=-0.18, p=0.19
Days To Death
cor=0.069, p=0.2
Last Follow Up
cor=0.037, p=0.46
Age 45 or Younger (0=N, 1=Y)
cor=-2.7e-05, p=1
Tumor Stage 1
cor=0.0054, p=0.91
Tumor Stage 2
cor=-0.036, p=0.47
Tumor Stage 3
cor=0.053, p=0.28
Tumor Stage 4
cor=0.14, p=0.0046
Node Stage 0
cor=-0.14, p=0.0046
Node Stage 1
cor=0.021, p=0.67
Node Stage 2
cor=0.011, p=0.82
Node Stage 3
cor=0.047, p=0.35
Metastasis Stage 1 (0=N, 1=Y)
cor=-0.011, p=0.82
Path Stage 1
cor=-0.00021, p=1
Path Stage 2
cor=-0.014, p=0.78
Path Stage 3
cor=0.068, p=0.17
Path Stage 4
cor=-0.44, p=2e-20
ER Status (0=Neg., 1=Pos.)
cor=-0.29, p=3.4e-09
PR Status (0=Neg., 1=Pos.)
cor=0.0081, p=0.87
Her2 Status (0=Neg., 1=Pos.)
cor=-0.19, p=0.00011
AR Levels Exp
Eigenprotein

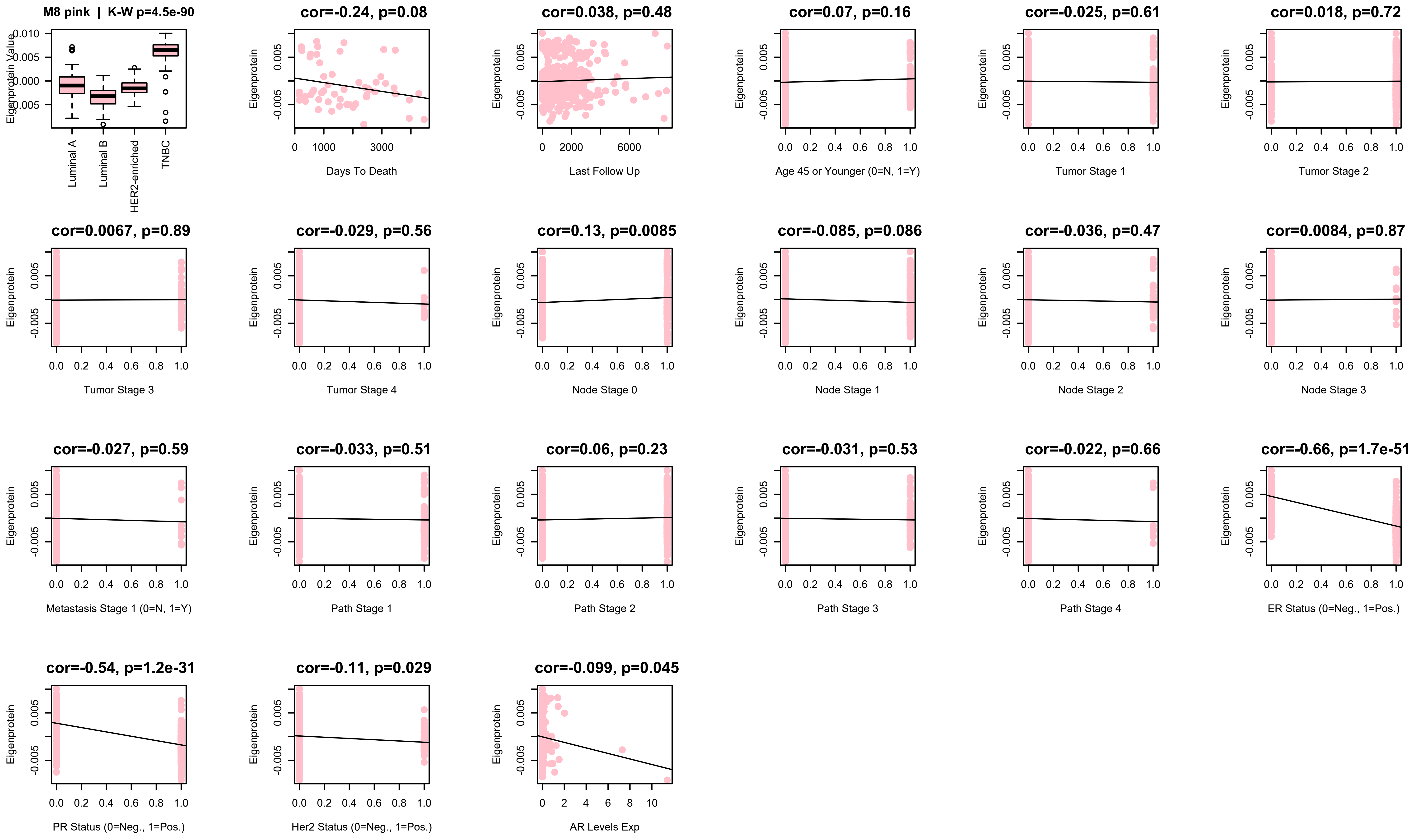

M8 pink | K-W p=4.5e-90
Eigenprotein Value
Luminal A
Luminal B
HER2-enriched
TNBC
cor=-0.24, p=0.08
Days To Death
cor=0.038, p=0.48
Last Follow Up
cor=0.07, p=0.16
Age 45 or Younger (0=N, 1=Y)
cor=-0.025, p=0.61
Tumor Stage 1
cor=0.018, p=0.72
Tumor Stage 2
cor=0.0067, p=0.89
Tumor Stage 3
cor=-0.029, p=0.56
Tumor Stage 4
cor=0.13, p=0.0085
Node Stage 0
cor=-0.085, p=0.086
Node Stage 1
cor=-0.036, p=0.47
Node Stage 2
cor=0.0084, p=0.87
Node Stage 3
cor=-0.027, p=0.59
Metastasis Stage 1 (0=N, 1=Y)
cor=-0.033, p=0.51
Path Stage 1
cor=0.06, p=0.23
Path Stage 2
cor=-0.031, p=0.53
Path Stage 3
cor=-0.022, p=0.66
Path Stage 4
cor=-0.66, p=1.7e-51
ER Status (0=Neg., 1=Pos.)
cor=-0.54, p=1.2e-31
PR Status (0=Neg., 1=Pos.)
cor=-0.11, p=0.029
Her2 Status (0=Neg., 1=Pos.)
cor=-0.099, p=0.045
AR Levels Exp
Eigenprotein

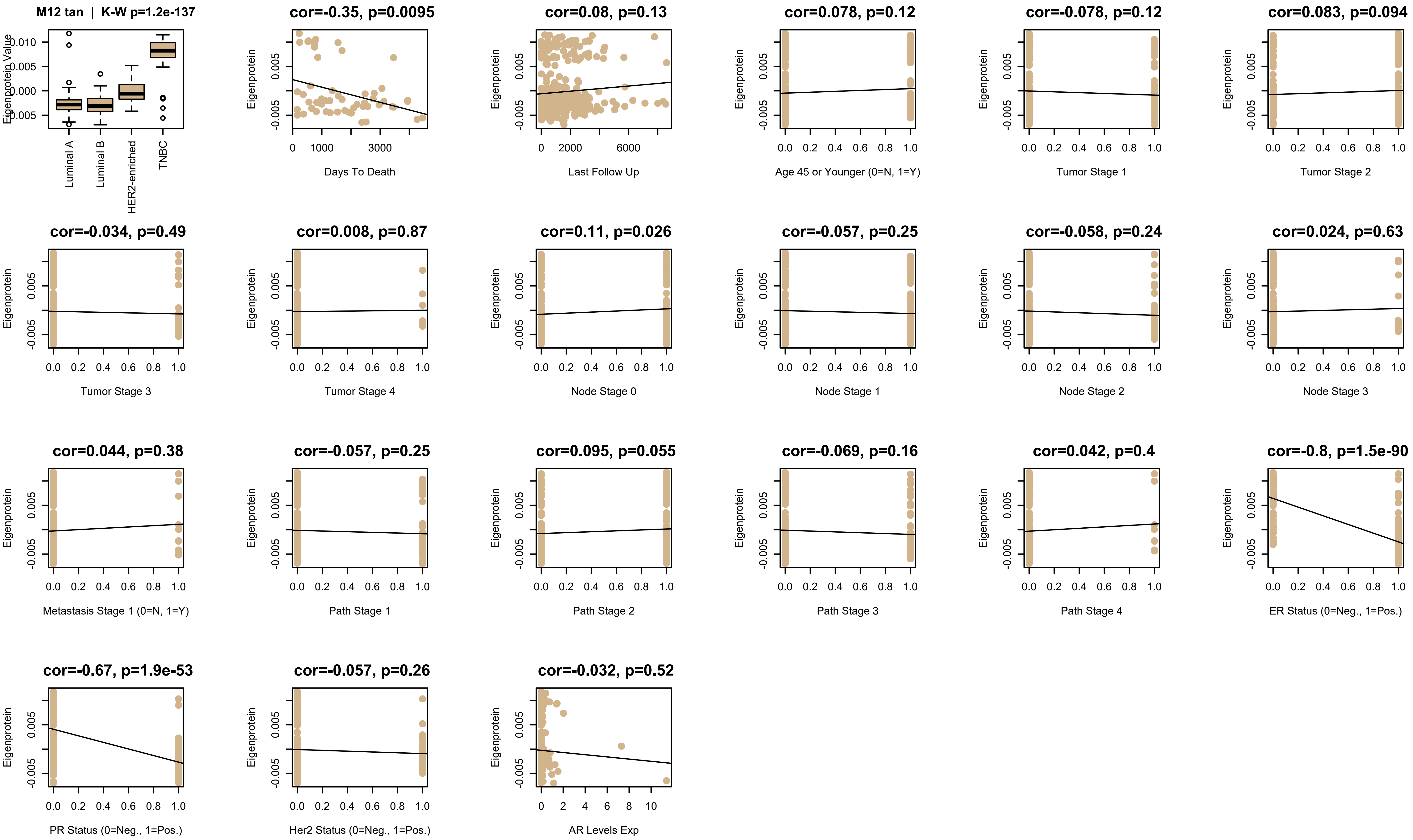
M12 tan | K-W p=1.2e-137
Eigenprotein Value
Luminal A
Luminal B
HER2-enriched
TNBC
cor=-0.35, p=0.0095
Days To Death
cor=0.08, p=0.13
Last Follow Up
cor=0.078, p=0.12
Age 45 or Younger (0=N, 1=Y)
cor=-0.078, p=0.12
Tumor Stage 1
cor=0.083, p=0.094
Tumor Stage 2
cor=-0.034, p=0.49
Tumor Stage 3
cor=0.008, p=0.87
Tumor Stage 4
cor=0.11, p=0.026
Node Stage 0
cor=-0.057, p=0.25
Node Stage 1
cor=-0.058, p=0.24
Node Stage 2
cor=0.024, p=0.63
Node Stage 3
cor=0.044, p=0.38
Metastasis Stage 1 (0=N, 1=Y)
cor=-0.057, p=0.25
Path Stage 1
cor=0.095, p=0.055
Path Stage 2
cor=-0.069, p=0.16
Path Stage 3
cor=0.042, p=0.4
Path Stage 4
cor=-0.8, p=1.5e-90
ER Status (0=Neg., 1=Pos.)
cor=-0.67, p=1.9e-53
PR Status (0=Neg., 1=Pos.)
cor=-0.057, p=0.26
Her2 Status (0=Neg., 1=Pos.)
cor=-0.032, p=0.52
AR Levels Exp
Eigenprotein

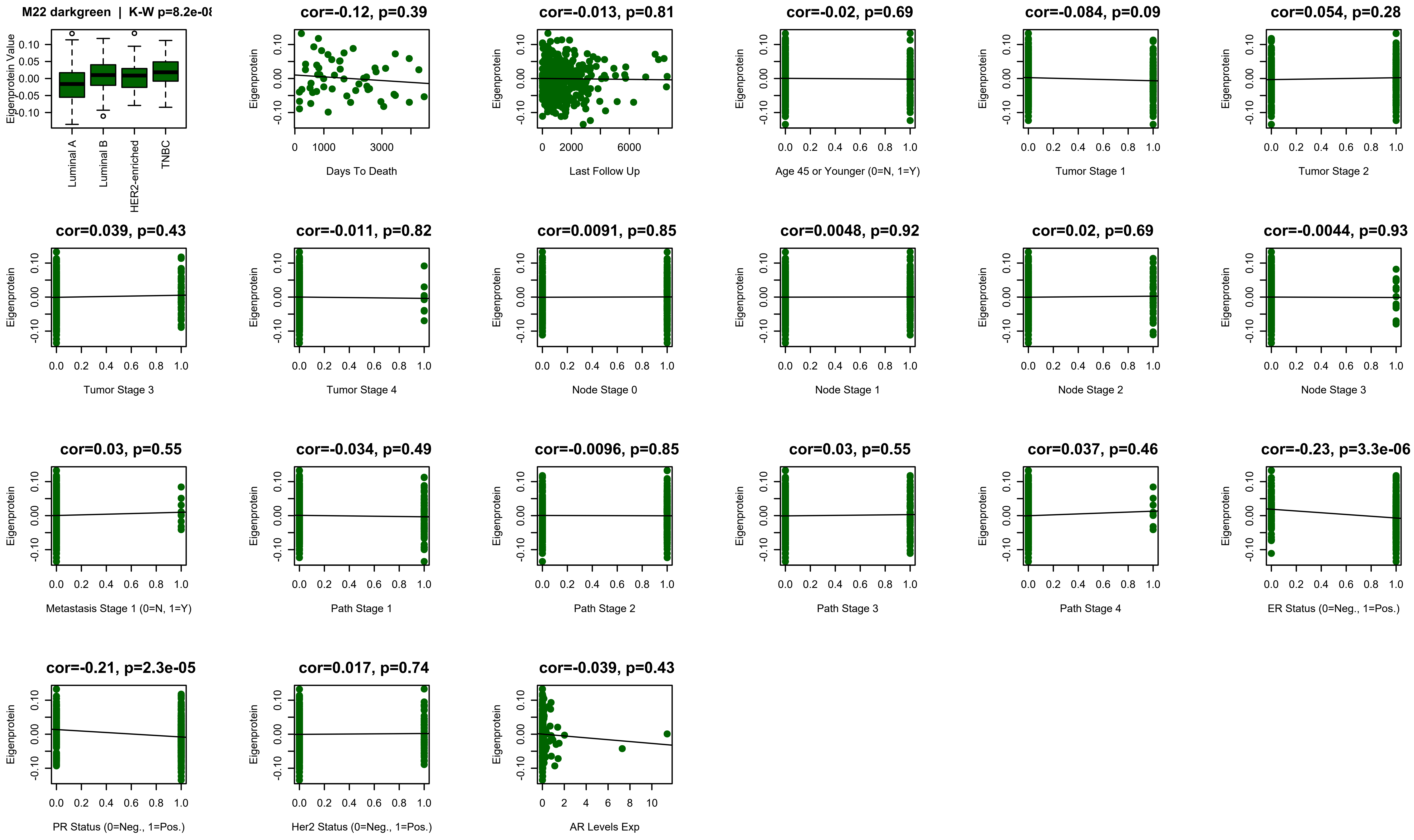
M22 darkgreen | K-W p=8.2e-08
Eigenprotein Value
Luminal A
Luminal B
HER2-enriched
TNBC
cor=-0.12, p=0.39
Days To Death
cor=-0.013, p=0.81
Last Follow Up
cor=-0.02, p=0.69
Age 45 or Younger (0=N, 1=Y)
cor=-0.084, p=0.09
Tumor Stage 1
cor=0.054, p=0.28
Tumor Stage 2
cor=0.039, p=0.43
Tumor Stage 3
cor=-0.011, p=0.82
Tumor Stage 4
cor=0.0091, p=0.85
Node Stage 0
cor=0.0048, p=0.92
Node Stage 1
cor=0.02, p=0.69
Node Stage 2
cor=-0.0044, p=0.93
Node Stage 3
cor=0.03, p=0.55
Metastasis Stage 1 (0=N, 1=Y)
cor=-0.034, p=0.49
Path Stage 1
cor=-0.0096, p=0.85
Path Stage 2
cor=0.03, p=0.55
Path Stage 3
cor=0.037, p=0.46
Path Stage 4
cor=-0.23, p=3.3e-06
ER Status (0=Neg., 1=Pos.)
cor=-0.21, p=2.3e-05
PR Status (0=Neg., 1=Pos.)
cor=0.017, p=0.74
Her2 Status (0=Neg., 1=Pos.)
cor=-0.039, p=0.43
AR Levels Exp
Eigenprotein

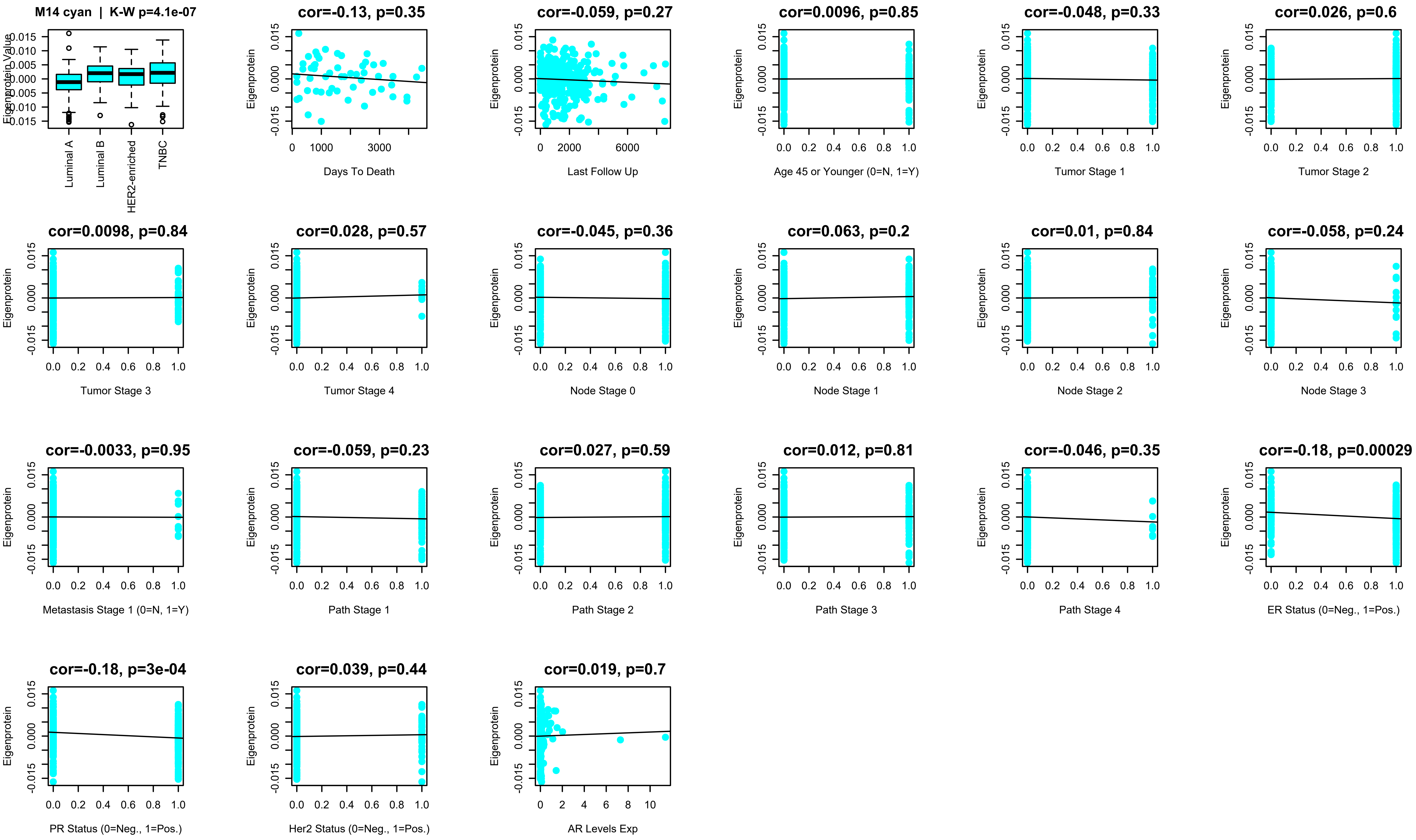
M14 cyan | K-W p=4.1e-07
Eigenprotein Value
Luminal A
Luminal B
HER2-enriched
TNBC
cor=-0.13, p=0.35
Days To Death
cor=-0.059, p=0.27
Last Follow Up
cor=0.0096, p=0.85
Age 45 or Younger (0=N, 1=Y)
cor=-0.048, p=0.33
Tumor Stage 1
cor=0.026, p=0.6
Tumor Stage 2
cor=0.0098, p=0.84
Tumor Stage 3
cor=0.028, p=0.57
Tumor Stage 4
cor=-0.045, p=0.36
Node Stage 0
cor=0.063, p=0.2
Node Stage 1
cor=0.01, p=0.84
Node Stage 2
cor=-0.058, p=0.24
Node Stage 3
cor=-0.0033, p=0.95
Metastasis Stage 1 (0=N, 1=Y)
cor=-0.059, p=0.23
Path Stage 1
cor=0.027, p=0.59
Path Stage 2
cor=0.012, p=0.81
Path Stage 3
cor=-0.046, p=0.35
Path Stage 4
cor=-0.18, p=0.00029
ER Status (0=Neg., 1=Pos.)
cor=-0.18, p=3e-04
PR Status (0=Neg., 1=Pos.)
cor=0.039, p=0.44
Her2 Status (0=Neg., 1=Pos.)
cor=0.019, p=0.7
AR Levels Exp
Eigenprotein

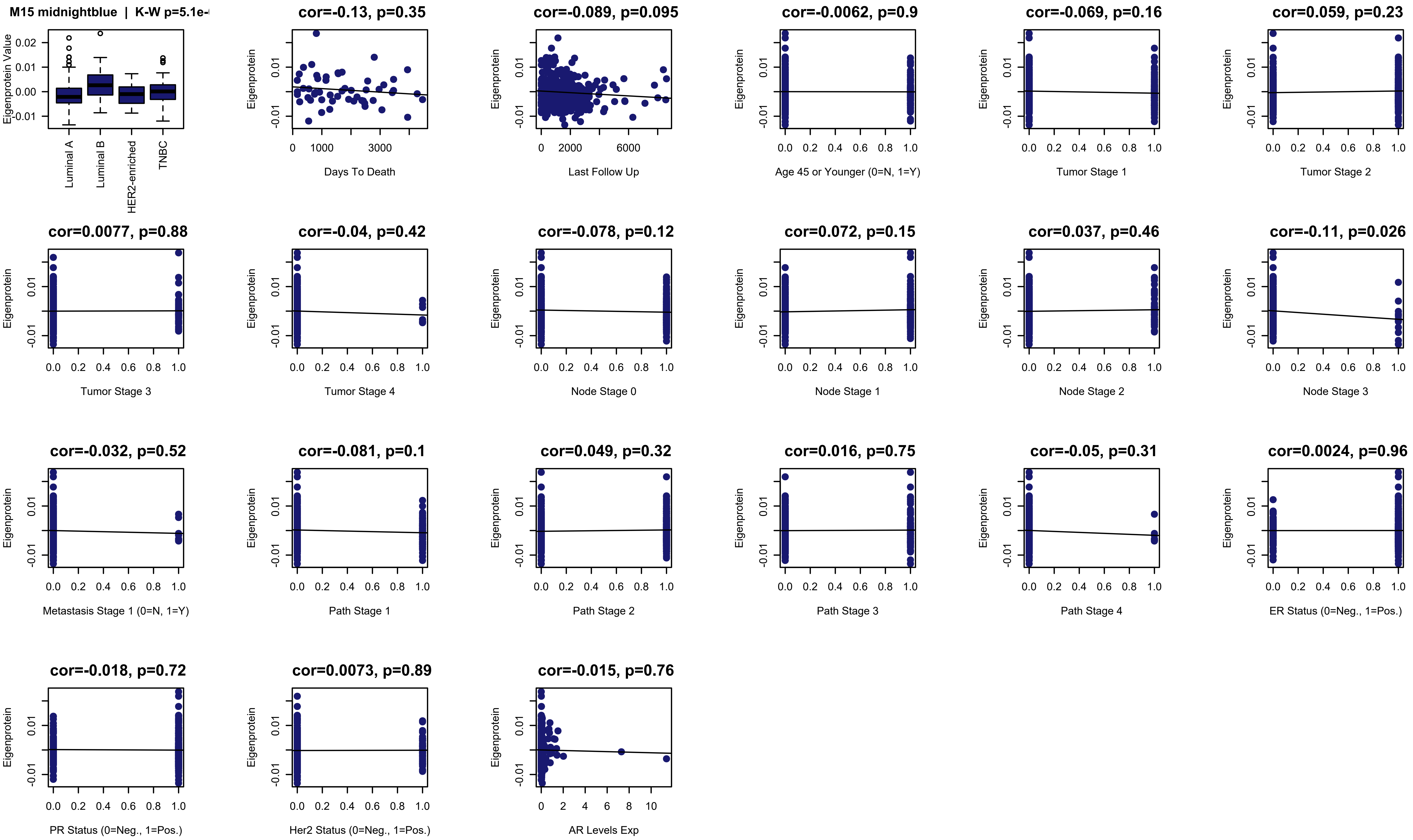
M15 midnightblue | K-W p=5.1e-
Eigenprotein Value
Luminal A
Luminal B
HER2-enriched
TNBC
cor=-0.13, p=0.35
Days To Death
cor=-0.089, p=0.095
Last Follow Up
cor=-0.0062, p=0.9
Age 45 or Younger (0=N, 1=Y)
cor=-0.069, p=0.16
Tumor Stage 1
cor=0.059, p=0.23
Tumor Stage 2
cor=0.0077, p=0.88
Tumor Stage 3
cor=-0.04, p=0.42
Tumor Stage 4
cor=-0.078, p=0.12
Node Stage 0
cor=0.072, p=0.15
Node Stage 1
cor=0.037, p=0.46
Node Stage 2
cor=-0.11, p=0.026
Node Stage 3
cor=-0.032, p=0.52
Metastasis Stage 1 (0=N, 1=Y)
cor=-0.081, p=0.1
Path Stage 1
cor=0.049, p=0.32
Path Stage 2
cor=0.016, p=0.75
Path Stage 3
cor=-0.05, p=0.31
Path Stage 4
cor=0.0024, p=0.96
ER Status (0=Neg., 1=Pos.)
cor=-0.018, p=0.72
PR Status (0=Neg., 1=Pos.)
cor=0.0073, p=0.89
Her2 Status (0=Neg., 1=Pos.)
cor=-0.015, p=0.76
AR Levels Exp
Eigenprotein

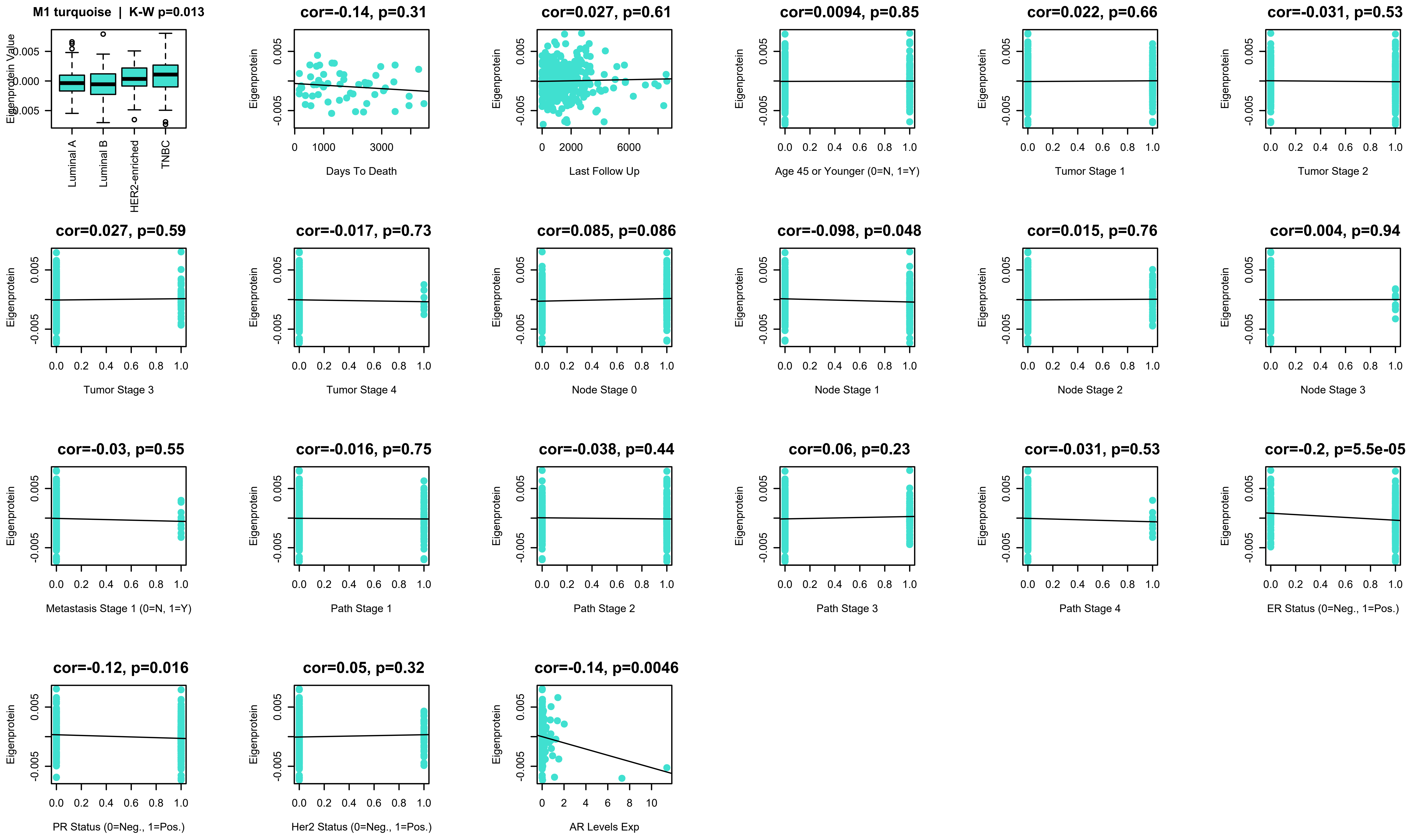
M1 turquoise | K-W p=0.013
Eigenprotein Value
Luminal A
Luminal B
HER2-enriched
TNBC
cor=-0.14, p=0.31
Days To Death
cor=0.027, p=0.61
Last Follow Up
cor=0.0094, p=0.85
Age 45 or Younger (0=N, 1=Y)
cor=0.022, p=0.66
Tumor Stage 1
cor=-0.031, p=0.53
Tumor Stage 2
cor=0.027, p=0.59
Tumor Stage 3
cor=-0.017, p=0.73
Tumor Stage 4
cor=0.085, p=0.086
Node Stage 0
cor=-0.098, p=0.048
Node Stage 1
cor=0.015, p=0.76
Node Stage 2
cor=0.004, p=0.94
Node Stage 3
cor=-0.03, p=0.55
Metastasis Stage 1 (0=N, 1=Y)
cor=-0.016, p=0.75
Path Stage 1
cor=-0.038, p=0.44
Path Stage 2
cor=0.06, p=0.23
Path Stage 3
cor=-0.031, p=0.53
Path Stage 4
cor=-0.2, p=5.5e-05
ER Status (0=Neg., 1=Pos.)
cor=-0.12, p=0.016
PR Status (0=Neg., 1=Pos.)
cor=0.05, p=0.32
Her2 Status (0=Neg., 1=Pos.)
cor=-0.14, p=0.0046
AR Levels Exp
Eigenprotein

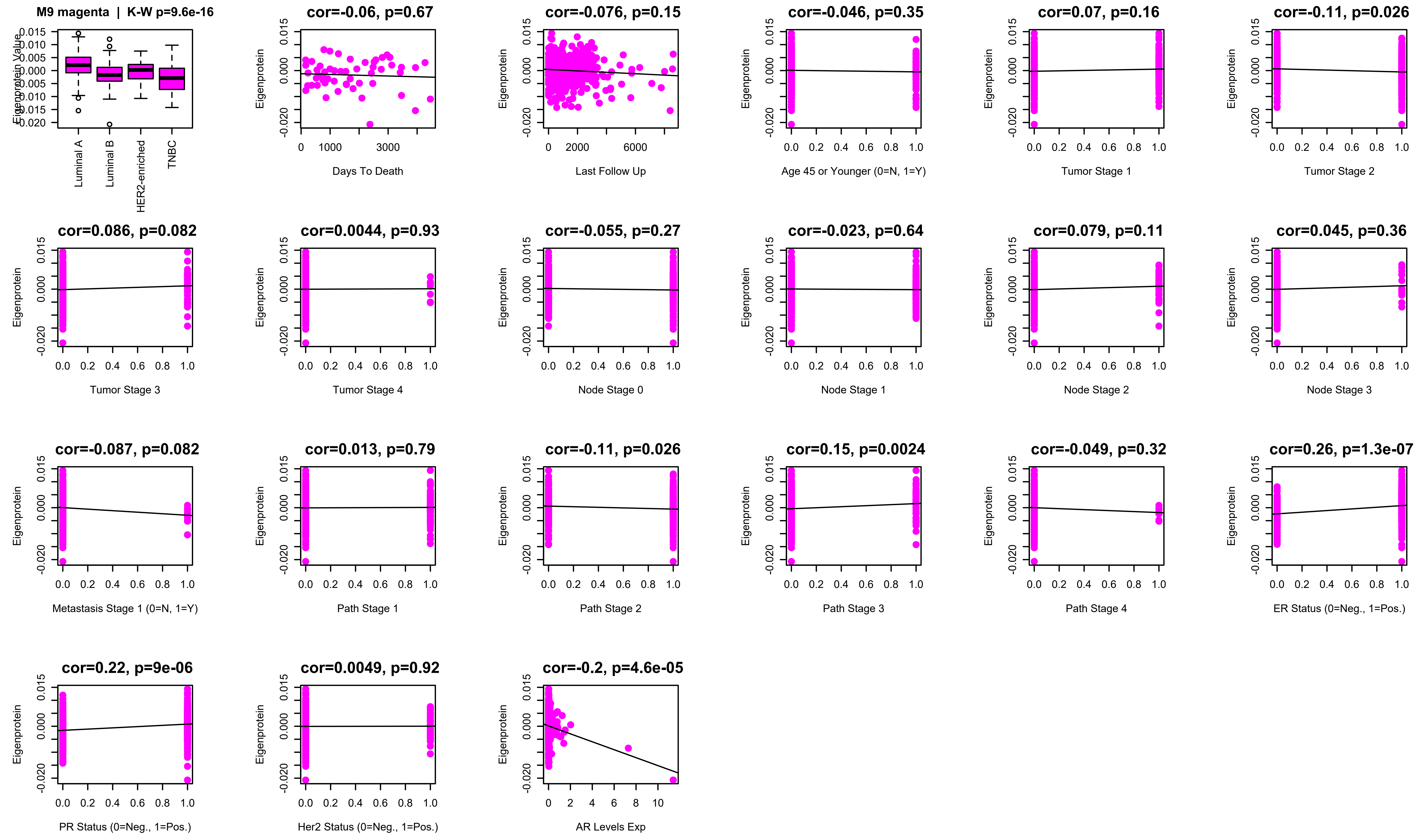
M9 magenta | K-W p=9.6e-16
Eigenprotein Value
Luminal A
Luminal B
HER2-enriched
TNBC
cor=-0.06, p=0.67
Days To Death
cor=-0.076, p=0.15
Last Follow Up
cor=-0.046, p=0.35
Age 45 or Younger (0=N, 1=Y)
cor=0.07, p=0.16
Tumor Stage 1
cor=-0.11, p=0.026
Tumor Stage 2
cor=0.086, p=0.082
Tumor Stage 3
cor=0.0044, p=0.93
Tumor Stage 4
cor=-0.055, p=0.27
Node Stage 0
cor=-0.023, p=0.64
Node Stage 1
cor=0.079, p=0.11
Node Stage 2
cor=0.045, p=0.36
Node Stage 3
cor=-0.087, p=0.082
Metastasis Stage 1 (0=N, 1=Y)
cor=0.013, p=0.79
Path Stage 1
cor=-0.11, p=0.026
Path Stage 2
cor=0.15, p=0.0024
Path Stage 3
cor=-0.049, p=0.32
Path Stage 4
cor=0.26, p=1.3e-07
ER Status (0=Neg., 1=Pos.)
cor=0.22, p=9e-06
PR Status (0=Neg., 1=Pos.)
cor=0.0049, p=0.92
Her2 Status (0=Neg., 1=Pos.)
cor=-0.2, p=4.6e-05
AR Levels Exp
Eigenprotein

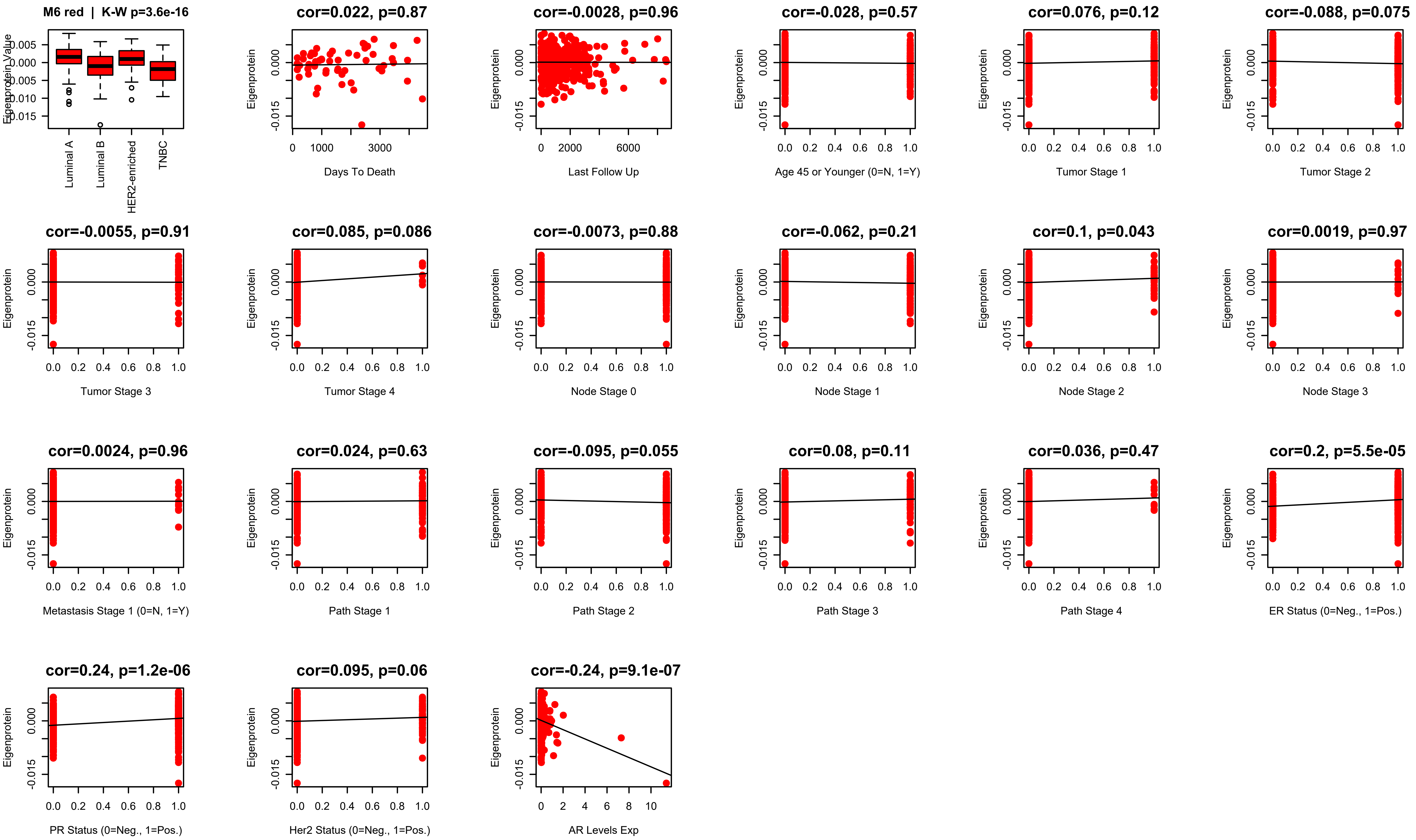
M6 red | K-W p=3.6e-16
Eigenprotein Value
Luminal A
Luminal B
HER2-enriched
TNBC
cor=0.022, p=0.87
Days To Death
cor=-0.0028, p=0.96
Last Follow Up
cor=-0.028, p=0.57
Age 45 or Younger (0=N, 1=Y)
cor=0.076, p=0.12
Tumor Stage 1
cor=-0.088, p=0.075
Tumor Stage 2
cor=-0.0055, p=0.91
Tumor Stage 3
cor=0.085, p=0.086
Tumor Stage 4
cor=-0.0073, p=0.88
Node Stage 0
cor=-0.062, p=0.21
Node Stage 1
cor=0.1, p=0.043
Node Stage 2
cor=0.0019, p=0.97
Node Stage 3
cor=0.0024, p=0.96
Metastasis Stage 1 (0=N, 1=Y)
cor=0.024, p=0.63
Path Stage 1
cor=-0.095, p=0.055
Path Stage 2
cor=0.08, p=0.11
Path Stage 3
cor=0.036, p=0.47
Path Stage 4
cor=0.2, p=5.5e-05
ER Status (0=Neg., 1=Pos.)
cor=0.24, p=1.2e-06
PR Status (0=Neg., 1=Pos.)
cor=0.095, p=0.06
Her2 Status (0=Neg., 1=Pos.)
cor=-0.24, p=9.1e-07
AR Levels Exp
Eigenprotein

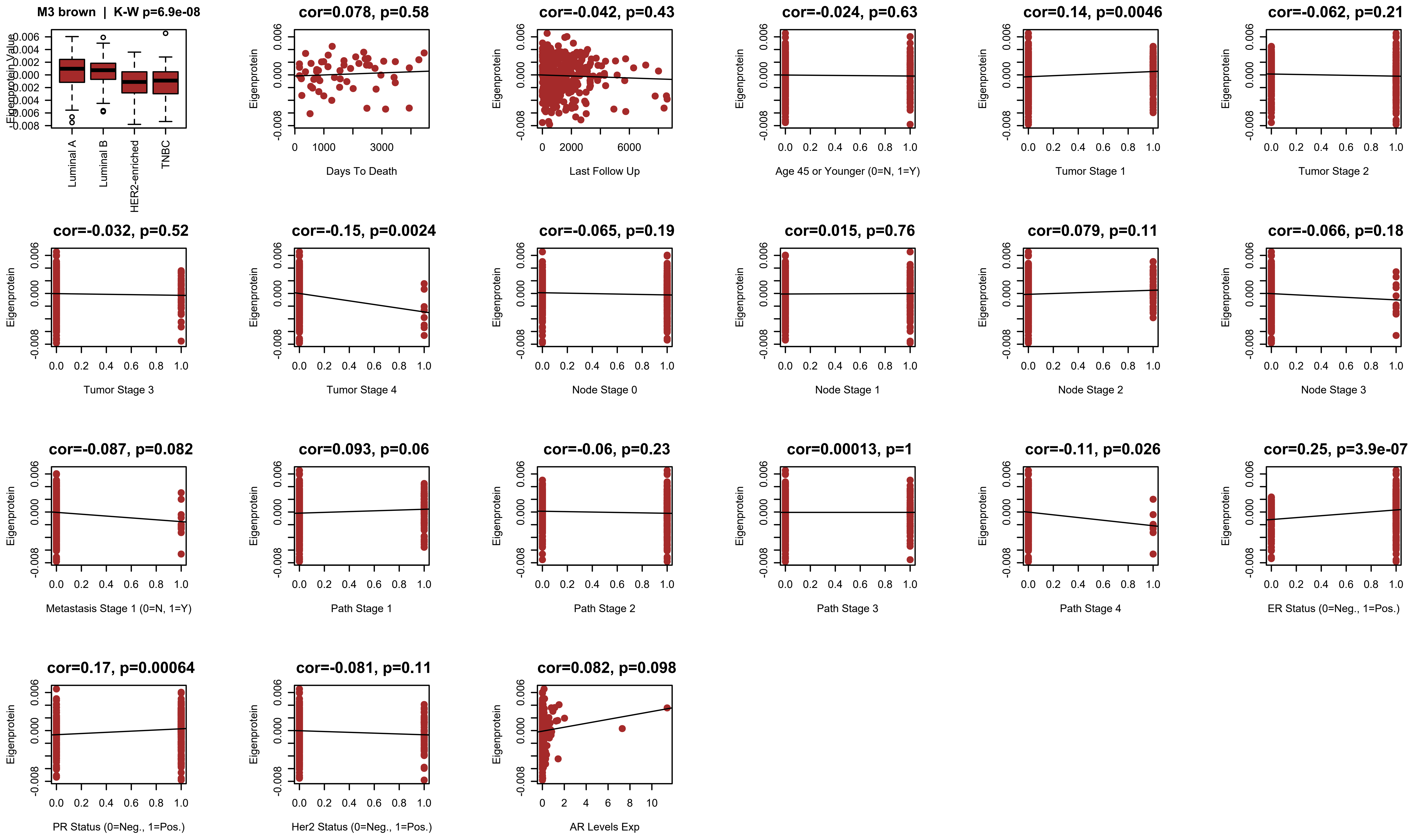
M3 brown | K-W p=6.9e-08
Eigenprotein Value
Luminal A
Luminal B
HER2-enriched
TNBC
cor=0.078, p=0.58
Days To Death
cor=-0.042, p=0.43
Last Follow Up
cor=-0.024, p=0.63
Age 45 or Younger (0=N, 1=Y)
cor=0.14, p=0.0046
Tumor Stage 1
cor=-0.062, p=0.21
Tumor Stage 2
cor=-0.032, p=0.52
Tumor Stage 3
cor=-0.15, p=0.0024
Tumor Stage 4
cor=-0.065, p=0.19
Node Stage 0
cor=0.015, p=0.76
Node Stage 1
cor=0.079, p=0.11
Node Stage 2
cor=-0.066, p=0.18
Node Stage 3
cor=-0.087, p=0.082
Metastasis Stage 1 (0=N, 1=Y)
cor=0.093, p=0.06
Path Stage 1
cor=-0.06, p=0.23
Path Stage 2
cor=0.00013, p=1
Path Stage 3
cor=-0.11, p=0.026
Path Stage 4
cor=0.25, p=3.9e-07
ER Status (0=Neg., 1=Pos.)
cor=0.17, p=0.00064
PR Status (0=Neg., 1=Pos.)
cor=-0.081, p=0.11
Her2 Status (0=Neg., 1=Pos.)
cor=0.082, p=0.098
AR Levels Exp
Eigenprotein

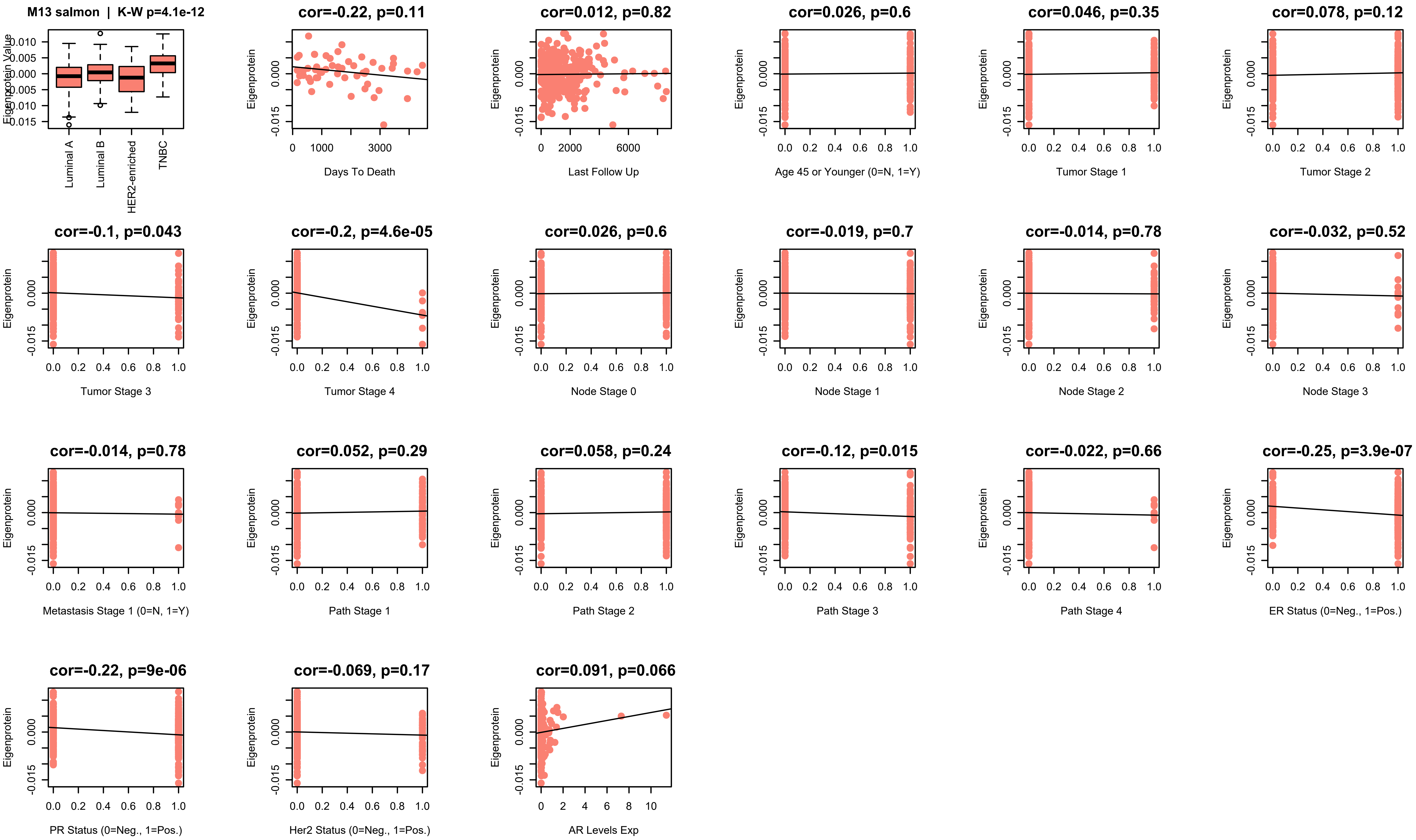
M13 salmon | K-W p=4.1e-12
Eigenprotein Value
Luminal A
Luminal B
HER2-enriched
TNBC
cor=-0.22, p=0.11
Days To Death
cor=0.012, p=0.82
Last Follow Up
cor=0.026, p=0.6
Age 45 or Younger (0=N, 1=Y)
cor=0.046, p=0.35
Tumor Stage 1
cor=0.078, p=0.12
Tumor Stage 2
cor=-0.1, p=0.043
Tumor Stage 3
cor=-0.2, p=4.6e-05
Tumor Stage 4
cor=0.026, p=0.6
Node Stage 0
cor=-0.019, p=0.7
Node Stage 1
cor=-0.014, p=0.78
Node Stage 2
cor=-0.032, p=0.52
Node Stage 3
cor=-0.014, p=0.78
Metastasis Stage 1 (0=N, 1=Y)
cor=0.052, p=0.29
Path Stage 1
cor=0.058, p=0.24
Path Stage 2
cor=-0.12, p=0.015
Path Stage 3
cor=-0.022, p=0.66
Path Stage 4
cor=-0.25, p=3.9e-07
ER Status (0=Neg., 1=Pos.)
cor=-0.22, p=9e-06
PR Status (0=Neg., 1=Pos.)
cor=-0.069, p=0.17
Her2 Status (0=Neg., 1=Pos.)
cor=0.091, p=0.066
AR Levels Exp
Eigenprotein

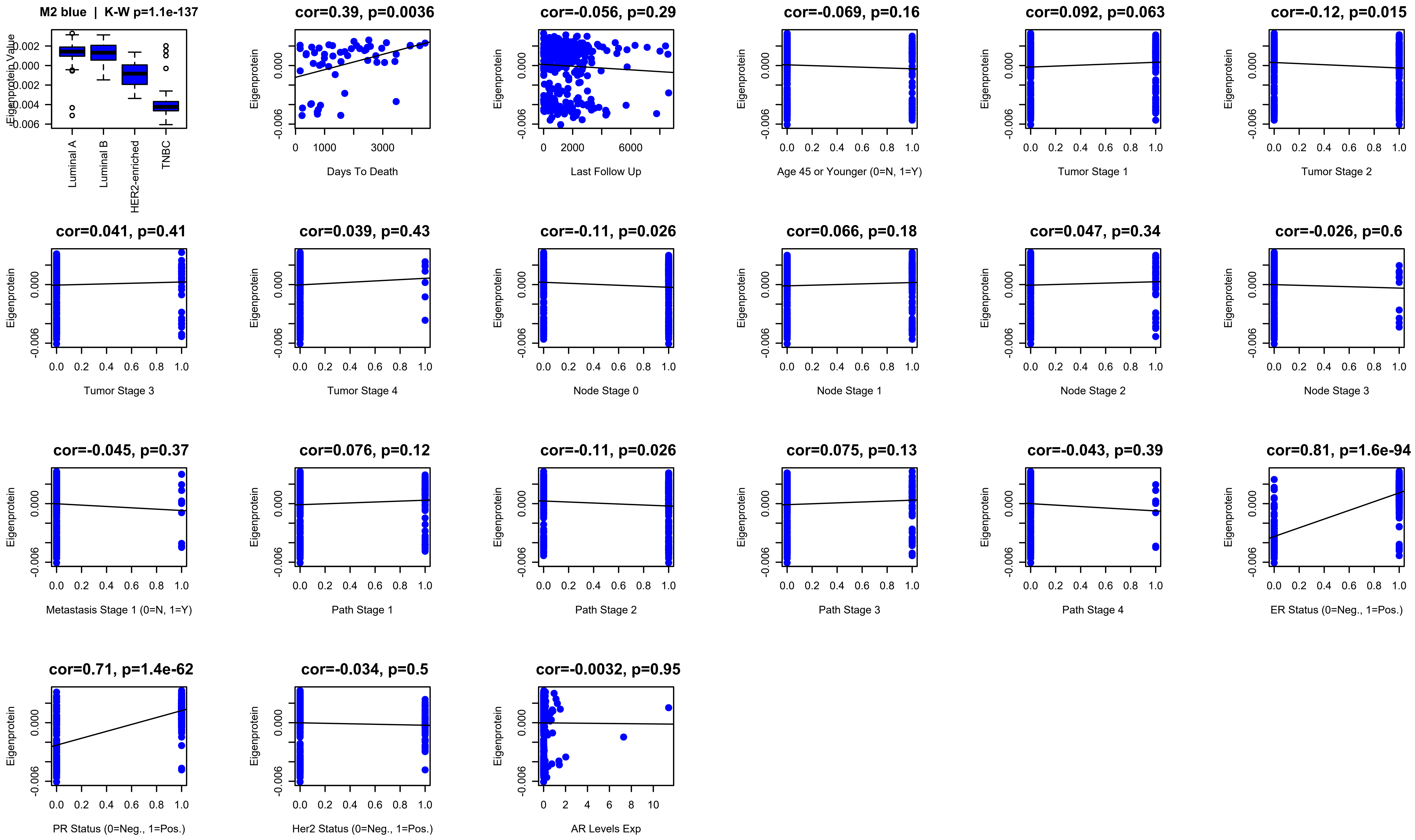
M2 blue | K-W p=1.1e-137
Eigenprotein Value
Luminal A
Luminal B
HER2-enriched
TNBC
cor=0.39, p=0.0036
Days To Death
cor=-0.056, p=0.29
Last Follow Up
cor=-0.069, p=0.16
Age 45 or Younger (0=N, 1=Y)
cor=0.092, p=0.063
Tumor Stage 1
cor=-0.12, p=0.015
Tumor Stage 2
cor=0.041, p=0.41
Tumor Stage 3
cor=0.039, p=0.43
Tumor Stage 4
cor=-0.11, p=0.026
Node Stage 0
cor=0.066, p=0.18
Node Stage 1
cor=0.047, p=0.34
Node Stage 2
cor=-0.026, p=0.6
Node Stage 3
cor=-0.045, p=0.37
Metastasis Stage 1 (0=N, 1=Y)
cor=0.076, p=0.12
Path Stage 1
cor=-0.11, p=0.026
Path Stage 2
cor=0.075, p=0.13
Path Stage 3
cor=-0.043, p=0.39
Path Stage 4
cor=0.81, p=1.6e-94
ER Status (0=Neg., 1=Pos.)
cor=0.71, p=1.4e-62
PR Status (0=Neg., 1=Pos.)
cor=-0.034, p=0.5
Her2 Status (0=Neg., 1=Pos.)
cor=-0.0032, p=0.95
AR Levels Exp
Eigenprotein

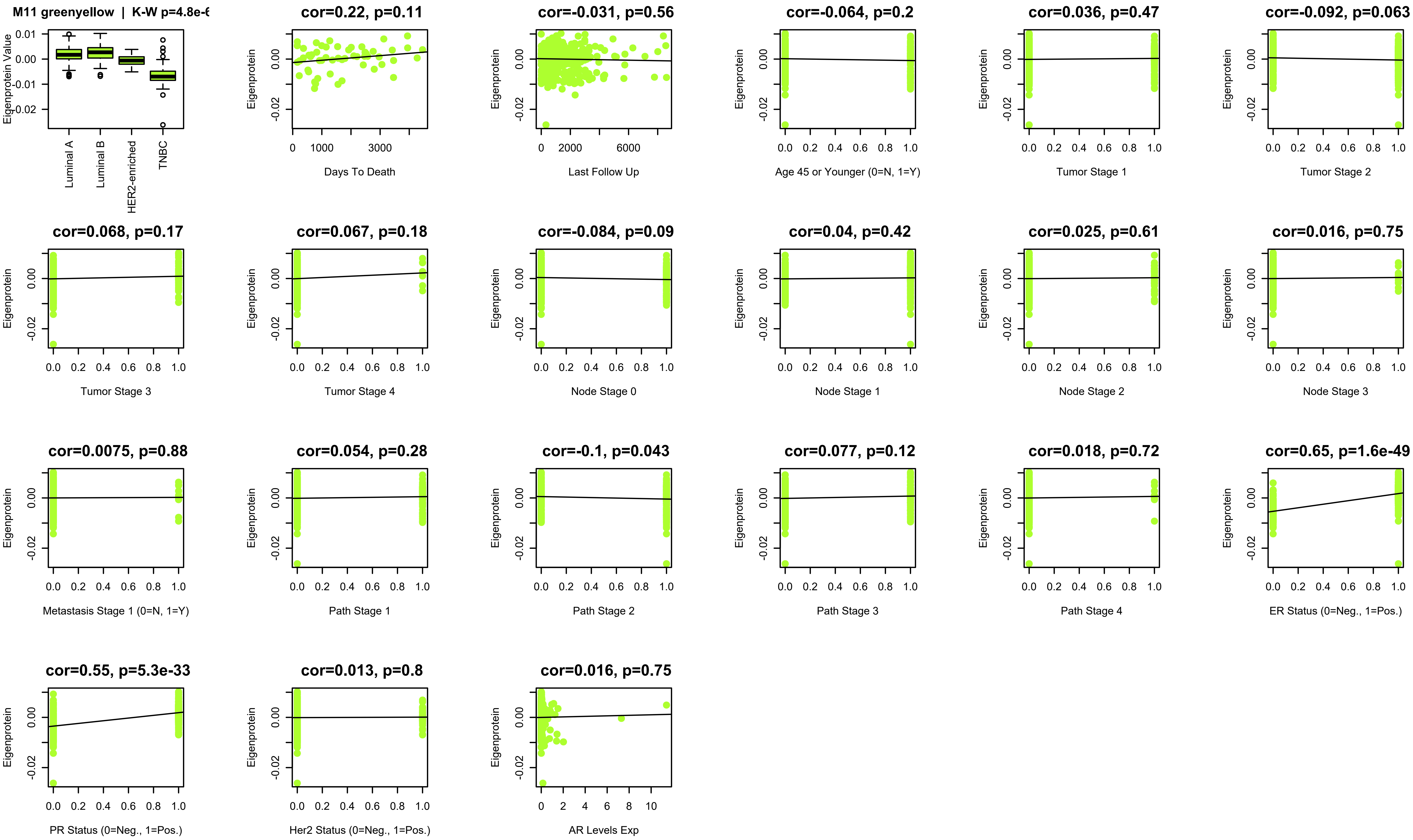
M11 greenyellow | K-W p=4.8e-6
Eigenprotein Value
Luminal A
Luminal B
HER2-enriched
TNBC
Eigenprotein
cor=0.22, p=0.11
Days To Death
cor=-0.031, p=0.56
Last Follow Up
cor=-0.064, p=0.2
Age 45 or Younger (0=N, 1=Y)
cor=0.036, p=0.47
Tumor Stage 1
cor=-0.092, p=0.063
Tumor Stage 2
cor=0.068, p=0.17
Tumor Stage 3
cor=0.067, p=0.18
Tumor Stage 4
cor=-0.084, p=0.09
Node Stage 0
cor=0.04, p=0.42
Node Stage 1
cor=0.025, p=0.61
Node Stage 2
cor=0.016, p=0.75
Node Stage 3
cor=0.0075, p=0.88
Metastasis Stage 1 (0=N, 1=Y)
cor=0.054, p=0.28
Path Stage 1
cor=-0.1, p=0.043
Path Stage 2
cor=0.077, p=0.12
Path Stage 3
cor=0.018, p=0.72
Path Stage 4
cor=0.65, p=1.6e-49
ER Status (0=Neg., 1=Pos.)
cor=0.55, p=5.3e-33
PR Status (0=Neg., 1=Pos.)
cor=0.013, p=0.8
Her2 Status (0=Neg., 1=Pos.)
cor=0.016, p=0.75
AR Levels Exp

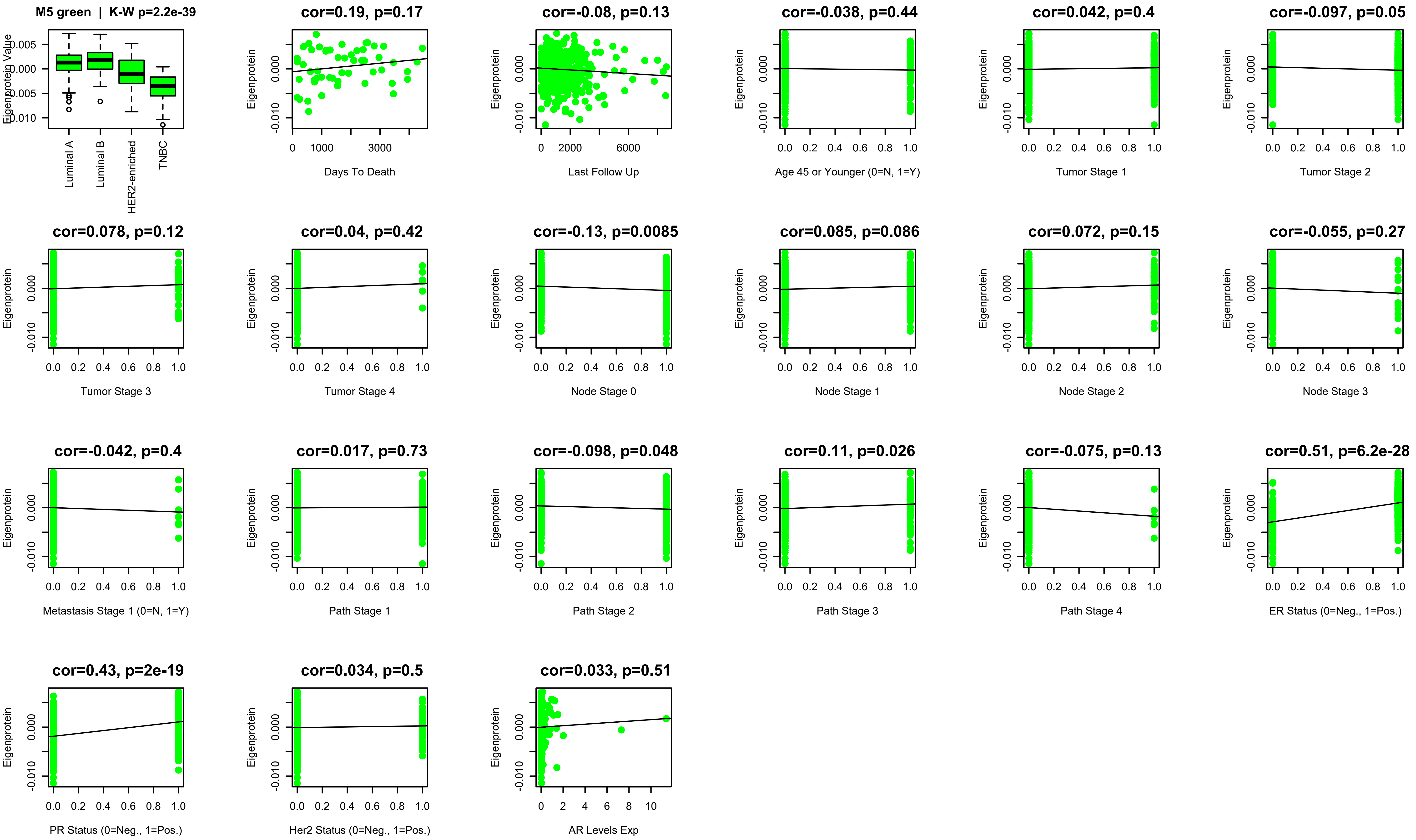
M5 green | K-W p=2.2e-39
Eigenprotein Value
Luminal A
Luminal B
HER2-enriched
TNBC
cor=0.19, p=0.17
Days To Death
cor=-0.08, p=0.13
Last Follow Up
cor=-0.038, p=0.44
Age 45 or Younger (0=N, 1=Y)
cor=0.042, p=0.4
Tumor Stage 1
cor=-0.097, p=0.05
Tumor Stage 2
cor=0.078, p=0.12
Tumor Stage 3
cor=0.04, p=0.42
Tumor Stage 4
cor=-0.13, p=0.0085
Node Stage 0
cor=0.085, p=0.086
Node Stage 1
cor=0.072, p=0.15
Node Stage 2
cor=-0.055, p=0.27
Node Stage 3
cor=-0.042, p=0.4
Metastasis Stage 1 (0=N, 1=Y)
cor=0.017, p=0.73
Path Stage 1
cor=-0.098, p=0.048
Path Stage 2
cor=0.11, p=0.026
Path Stage 3
cor=-0.075, p=0.13
Path Stage 4
cor=0.51, p=6.2e-28
ER Status (0=Neg., 1=Pos.)
cor=0.43, p=2e-19
PR Status (0=Neg., 1=Pos.)
cor=0.034, p=0.5
Her2 Status (0=Neg., 1=Pos.)
cor=0.033, p=0.51
AR Levels Exp
Eigenprotein

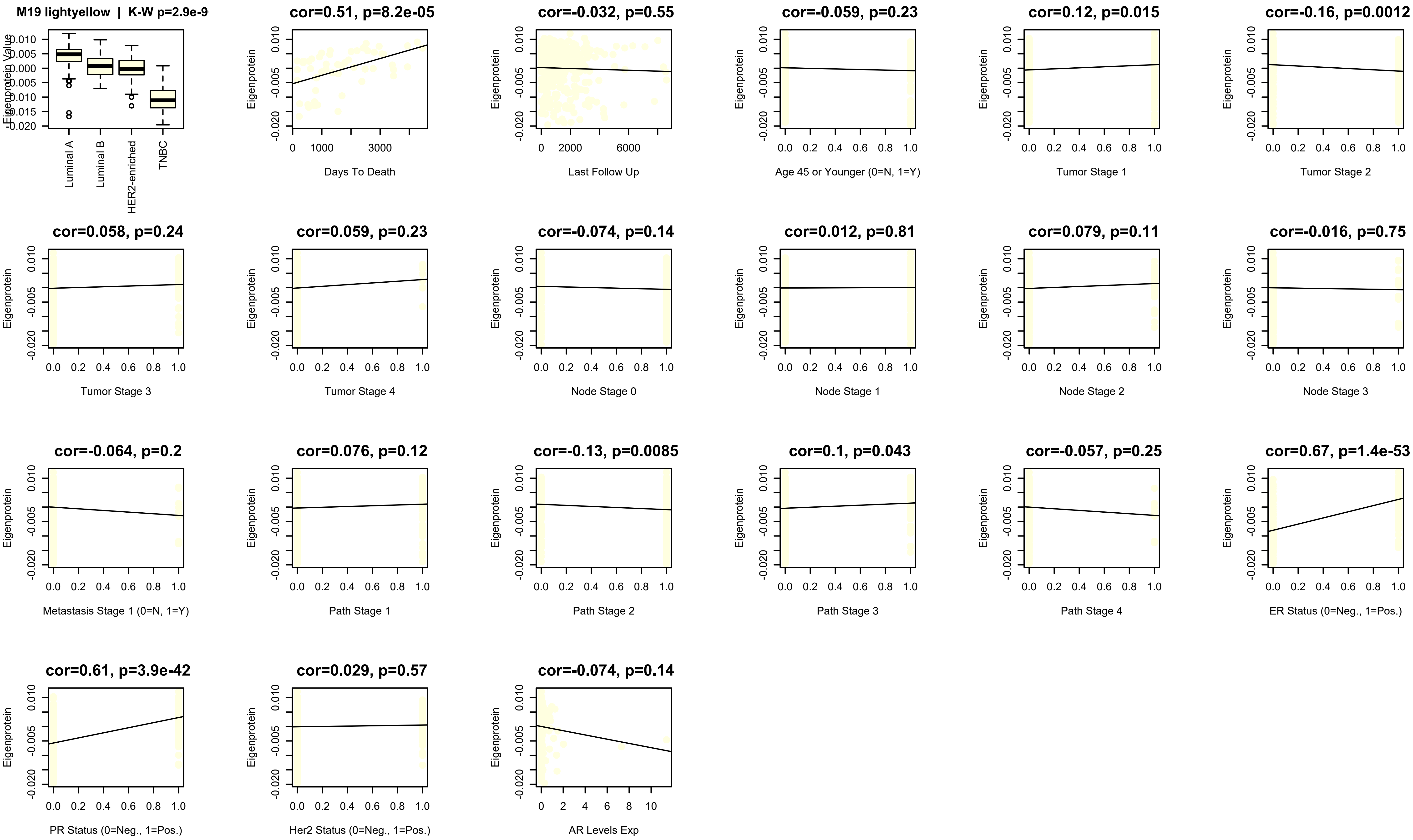

M19 lightyellow | K-W p=2.9e-9
Eigenprotein Value
Luminal A
Luminal B
HER2-enriched
TNBC
cor=0.51, p=8.2e-05
Days To Death
cor=-0.032, p=0.55
Last Follow Up
cor=-0.059, p=0.23
Age 45 or Younger (0=N, 1=Y)
cor=0.12, p=0.015
Tumor Stage 1
cor=-0.16, p=0.0012
Tumor Stage 2
cor=0.058, p=0.24
Tumor Stage 3
cor=0.059, p=0.23
Tumor Stage 4
cor=-0.074, p=0.14
Node Stage 0
cor=0.012, p=0.81
Node Stage 1
cor=0.079, p=0.11
Node Stage 2
cor=-0.016, p=0.75
Node Stage 3
cor=-0.064, p=0.2
Metastasis Stage 1 (0=N, 1=Y)
cor=0.076, p=0.12
Path Stage 1
cor=-0.13, p=0.0085
Path Stage 2
cor=0.1, p=0.043
Path Stage 3
cor=-0.057, p=0.25
Path Stage 4
cor=0.67, p=1.4e-53
ER Status (0=Neg., 1=Pos.)
cor=0.61, p=3.9e-42
PR Status (0=Neg., 1=Pos.)
cor=0.029, p=0.57
Her2 Status (0=Neg., 1=Pos.)
cor=-0.074, p=0.14
AR Levels Exp
Eigenprotein
